# Deciphering interferon functions in avian influenza using receptor knockout models in the natural host

**DOI:** 10.7554/eLife.107855

**Published:** 2026-06-26

**Authors:** Mohanned Naif Alhussien, Hanna Kaisa Vikkula, Romina Klinger, Christian Zenner, Simon P Früh, Rashi Negi, Theresa von Heyl, Sabrina Schleibinger, Milena Brunner, Tom VL Berghof, Leora Avolio, Arne Reich, Benjamin Schade, Bassel Aboukhadra, Silke Rautenschlein, Rudolf Preisinger, Hicham Sid, Benjamin Schusser

**Affiliations:** 1 TUM School of Life Sciences, Weihenstephan, Department of Molecular Life Sciences, Reproductive Biotechnology, Technical University of Munich Freising Germany; 2 Department of Veterinary Sciences, Ludwig-Maximilians-Universität München Munich Germany; 3 Institute of Virology, Freie Universität Berlin Berlin Germany; 4 Bavarian Animal Health Service, Department of Pathology Poing Germany; 5 Clinic for Poultry, University of Veterinary Medicine Hannover Hannover Germany; 6 EW GROUP GmbH Visbek Germany; 7 Center for Infection Prevention (ZIP), Technical University of Munich Freising Germany; The University of Texas Southwestern Medical Center United States; The University of Texas Southwestern Medical Center United States

**Keywords:** chicken, interferons, genetic modification, CRISPR/Cas9, avian influenza, Chicken

## Abstract

The rapid cross-species transmission of highly pathogenic avian influenza presents a significant zoonotic threat. Elucidating the avian interferon (IFN) system, the primary antiviral defense in chickens, is critical for controlling the virus at its source and preventing its spillover into humans and other species. We engineered type I (IFN-α/β) and type III (IFN-λ) IFN receptor knockout chickens to dissect the role of IFNs in viral infections. Results revealed that type I IFN predominantly modulates innate immune cell populations, T cell subsets, and their contribution to antibody production following immunization under physiological conditions. In ovo and in vivo challenge experiments utilizing diverse influenza A virus strains demonstrated strain-specific roles of both IFN-α/β and IFN-λ in orchestrating viral pathogenesis, immunological responses, and tissue-tropism effects. Notably, type I IFN was particularly crucial in the initial defense mechanisms against H3N1 avian influenza A virus infection. These novel models offer unprecedented insights into avian IFN biology within the context of avian influenza, which is essential for developing more effective strategies to prevent and control this public health challenge.

## Introduction

Chickens are highly susceptible to viral pathogens such as avian influenza A virus (AIV), a zoonotic virus transmissible to humans, which causes significant economic losses in the poultry industry (Peacock et al., 2019; Pinotti et al., 2024; Wong and Yuen, 2006). In 2022, 67 countries across five continents reported outbreaks of H5N1 highly pathogenic avian influenza, losing over 131 million domestic poultry. The disease continued to spread as 14 more countries reported outbreaks, primarily across the Americas (World Health Organization, 2023). The emergence of various zoonotic avian influenza strains has heightened global concerns about the potential for these strains to mutate and sustain human-to-human transmission, possibly leading to a pandemic (Horman et al., 2018; Peacock et al., 2025). Despite substantial advancements in our comprehension of the mammalian host response to pathogenic insults, knowledge of immune mechanisms in avian species, the natural hosts of the virus, remains limited. As a result, strategies to control AIV have heavily relied on biosecurity measures and depopulation of infected flocks, imposing substantial economic burdens on the poultry industry and amounting to billions of dollars in losses (Seeger et al., 2021). The insufficiency of these traditional approaches in managing the growing prevalence of highly pathogenic AIV outbreaks has driven interest in alternative antiviral therapies (Pinotti et al., 2024; Wong and Yuen, 2006). Notably, France and the Netherlands have implemented domestic duck vaccination as a control measure against highly pathogenic avian influenza outbreaks (Guinat et al., 2025).

Interferons (IFNs) were first identified in chicken embryos. These proteins are characterized by their ability to inhibit viral replication, a property that led to their designation (Lindenmann et al., 1957). IFNs are cytokines that induce the expression of a myriad of IFN-stimulated genes (ISGs) that act on innate and adaptive immunity, eliciting an antiviral state against invading viral pathogens (Durbin et al., 2013; Reuter et al., 2014). They are classified based on their molecular structure, receptor specificity, and induction pathways. In chickens, type I IFNs (IFN-α and IFN-β) and type III IFN (IFN-λ) play critical roles, with IFN-α and IFN-λ being the primary focus of immunological studies due to their significant involvement in antiviral responses. To accomplish the primary function of IFNs, these cytokines need to bind to their respective receptors. The type III IFN receptor is a heterodimer consisting of the ubiquitously expressed receptor chain IL-10Rβ and the epithelium-specific receptor chain IFNLR1 (IL-28Rα). In contrast, type I IFN (IFNAR1/IFNAR2) receptors demonstrate widespread expression across diverse tissues and cell types throughout the body (Challagulla et al., 2023; Santhakumar et al., 2017).

The molecular cloning and characterization of chicken IFN-λ were relatively recent, uncovering antiviral properties comparable to those of mammalian IFN-λ (Reuter et al., 2014; Karpala et al., 2008). However, despite this progress, the mechanisms and functional roles of type I and III IFNs in chickens remain largely uncharacterized. Murine IFN-λ is the initial IFN produced during a viral invasion, providing epithelial antiviral protection against low viral loads without eliciting an inflammatory response (Galani et al., 2017). When the infection surpasses the control of IFN-λ, type I IFN is activated, associated with an inflammatory response and immunopathology. Unlike the widespread effects of type I IFN, the similarity of IFNLR1 expression with the tissue tropism of influenza A virus allows mammalian IFN-λ to specifically target cell types at risk of infection. This effectively induces antiviral genes in these cells, aiding in controlling influenza A virus spread without overstimulating the immune system and causing immunopathology (Davidson et al., 2016; Lozhkov et al., 2020). IFNAR1 knockout (KO) mice show increased susceptibility to several viral infections, but not influenza. However, mice lacking IFNAR1 and IFNLR1 cannot control influenza infection (Mordstein et al., 2008; Mordstein et al., 2010). Researchers have shown that mammalian IFN-λ can also have pro-inflammatory effects by disrupting the lung epithelial barrier, hindering lung repair, and impairing epithelial proliferation during influenza infections (Broggi et al., 2020; Major et al., 2020).

Despite the discovery of IFNs in chickens, our knowledge of the avian IFN system remains limited (Lindenmann et al., 1957). Unraveling the complexities of the IFN system is crucial for devising strategies to control avian influenza in its natural host, thereby preventing zoonotic transmission to humans and other species. This limitation is primarily due to the complex and overlapping signaling pathways of type I and type III IFNs, which induce similar sets of ISGs and produce almost indistinguishable biological activities despite signaling through distinct receptors (Durbin et al., 2013). Furthermore, our understanding of ISGs and their antiviral functions in chickens is primarily based on research from humans and other mammals, which may not fully account for the fundamental differences in innate immunity between chickens and mammals (Reuter et al., 2014; Santhakumar et al., 2017; Penski et al., 2011). The lack of appropriate biotechnological tools, such as genetically engineered chickens deficient in IFN receptors, hinders progress in this field. These challenges make it difficult to elucidate the individual antiviral and immunomodulatory roles of type I and type III IFNs in avian research despite the potential value of these cytokines as targets for developing therapeutics against a broader range of viruses (Santhakumar et al., 2017; Schoggins, 2014).

To address this gap, we utilized chicken primordial germ cells (PGCs) to generate IFNAR1 and IFNLR1 KO chicken lines. These genetically modified lines allow for the selective study of type I and type III IFN responses. Through experiments conducted in vitro, in ovo, and in vivo, we investigated the impact of these IFN classes on various physiological functions, antibody responses to immunization, and host-pathogen interactions. Notably, in vivo H3N1 infections revealed distinct and novel mechanisms for both types of chicken IFN in the context of H3N1 defense. These newly developed chicken lines provide critical insights into the roles of IFN-α/β and IFN-λ in immune responses during various viral infections, including influenza and infectious bronchitis virus (IBV). The knowledge gained from these studies is fundamental for developing therapeutic methods and immunomodulatory strategies to control and eradicate the significant public health challenges in avian species.

## Results

### Successful generation of IFN receptor KO chickens

**Figure 1.**
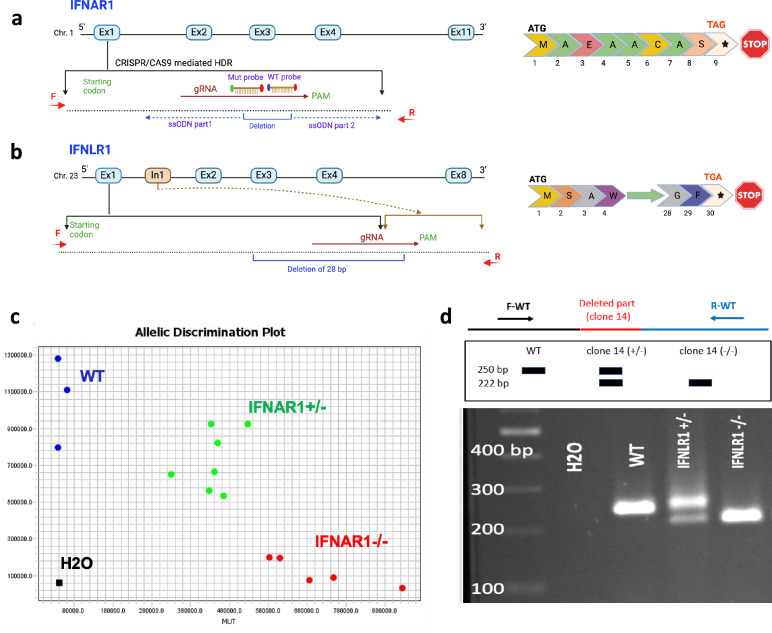
Targeting strategy and genotype for knockout (KO) of chicken IFN-α/β receptor (IFNAR1) and IFN-λ receptor (IFNLR1). (**a**) Guide RNAs designed to target the coding region within exon 1 of the IFNAR1 chain of the IFN-α/β receptor and the epithelium-specific chain (IFNLR1) of the IFN-λ receptor. For IFNAR1, the INDEL was pre-designed using CRISPR-mediated homology-directed repair (HDR) technology. The IFNAR1 KO clone has a 7 bp deletion and produces a stop codon after 21 bp. (**b**) For IFNLR1-positive primordial germ cell (PGC) clones, the clone with the maximum deletion (28 bp) was selected as it is suitable to design IFNLR1 KO-specific PCR genotyping assay and produce a stop codon after 87 bp. (**c**) TaqMan genotyping assay for IFNAR1. (**d**) PCR genotyping assay for IFNLR1. Labeled molecular weight markers are shown in panel d. Figure 1—source data 1.Labeled uncropped gel image for the IFNLR1 PCR genotyping assay shown in Figure 1d, indicating the relevant lanes and expected bands. Figure 1—source data 2.Original uncropped gel image file for the IFNLR1 PCR genotyping assay displayed in Figure 1d.

Knocking out the IFNAR1 or IFNLR1 receptors generates animal models deficient in type I or type III IFN signaling, respectively. This receptor-targeted approach, in contrast to the cytokine-targeted approach, is especially effective in chickens, where multiple IFN-α genes exist, as it blocks the entire signaling pathway, allowing for comprehensive studies of IFN’s roles in immunity and viral responses (Challagulla et al., 2023; Santhakumar et al., 2017; McNab et al., 2015). Chickens lacking either the receptor of IFN-α/β (IFNAR1) or IFN-λ (IFNLR1) were generated by designing guide RNAs to target the coding region within exon 1 of the IFNAR1 chain of the IFN-α/β receptor and the epithelium-specific chain (IFNLR1) of the IFN-λ receptor. PGCs, precursors of sperm and oocytes, were co-transfected with a CRISPR/Cas9 vector expressing a single-guide RNA and Cas9-2A-eGFP targeting the coding region within exon 1 of the respective receptor genes (Figure 1a and b). To generate the IFNAR1 KO, homology-directed repair (HDR) utilizing single-stranded oligodeoxynucleotide (ssODN) templates was used to facilitate the targeted gene edits. Transient expression of eGFP in transfected PGCs allowed for the selection of successfully transfected cells via automated cell sorting. Subsequent Sanger sequencing confirmed several clonal PGC lines with the desired modifications for IFNAR1 and IFNLR1.

**Figure 2.**
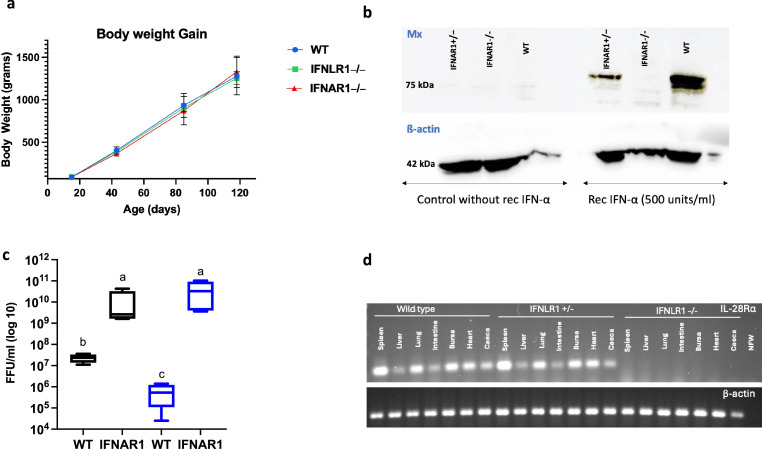
Growth and successful generation of genetically modified chickens. (**a**) Body weight development of WT, IFNLR1^−/−^, and IFNAR1^−/−^ chickens (n=12 per genotype). Data are presented as mean ± SEM. (**b**) Western blot confirms successful knockout of IFNAR1: WT, IFNAR1^+/−^, and IFNAR1^−/−^ chicken embryonic fibroblast cells (n=3) were cultured in a six-well plate and stimulated with recombinant IFN-α (500 U/ml) for 12 hr. For the control groups, chicken embryo fibroblast (CEF) cells were treated with CEF medium without recombinant IFN-α. After stimulation, the control and treatment groups’ CEF cells were trypsinized and collected for an SDS-PAGE and western blot experiment. The results demonstrate functional loss of type I IFN receptor (IFNAR1) in CEF IFNAR1^−/−^ cells treated with recombinant IFN-α, as the Mx protein (75 kDa) was observed solely in the CEF WT and IFNAR1^+/−^ cells treated with recombinant IFN-α. β-Actin was used as loading control and detected at 42 kDa. (**c**) WSN33 viral titration in WT and IFNAR1^−/−^ embryo pretreated with recombinant IFN-α. ED10 chicken embryos (blue columns) were stimulated with 1.5×10^5^ U of recombinant IFN-α in 100 μl PBS 12 hr before and at the time of infection. As controls (black columns) did not receive IFN-α stimulation. All the groups of embryos were infected with 1000 FFU of WSN33, and viral load in the allantoic fluid was analyzed 24 hr post-infection by titration on Madine-Darby canine kidney (MDCK) cells. Number of embryos, for the control groups (WT = 4, IFNAR1^−/−^=4), and treatment groups (WT = 4, IFNAR1^−/−^=4). Significance was calculated by one-way ANOVA. Statistically different groups (p≤0.05) are indicated with different superscript letters (**a, b, c**). (**d**) RT-PCR confirms functional loss of IFNLR1 in homozygous embryos: expression analysis of IFN-λ receptor (IL-28Rα) was studied in different tissues of 18-day-old embryos challenged with H3N1 to ensure induced expression of IL-28Rα. RNA was isolated via the TRIzol method after the genotyping of embryos. Complementary DNA (cDNA) was transcribed via GoScript Transcription Mix and Random Primers. PCR was performed with β-actin primers as control and IL-28Rα primers to check the presence of IL-28Rα receptors in tissues. Nuclease-free water (NFW) was used as a negative control for both. A 1.5% Tris-borate-EDTA (TBE) was used to visualize β-actin amplicon at 300 bp and a 2% TBE gel to visualize the 108 bp IL-28Rα amplicon. Figure 2—source data 1.Labeled uncropped western blot and RT-PCR gel images for Figure 2b and d, indicating the relevant lanes, treatments, bands, and molecular weight markers. Figure 2—source data 2.Original uncropped image files for the western blot and RT-PCR gel analyses displayed in Figure 2b and d.

For IFNLR1, the PGC clone with the largest deletion (28 bp) was selected, leading to a stop codon formation after 87 bp. Similarly, for IFNAR1, the clone with a 7 bp deletion produced a stop codon after 21 bp (Figure 1a and b). These clones were used for the generation of germline chimeras. Selected chimeric roosters demonstrated 4% and 7% germline transmission rates for IFNAR1 and IFNLR1 modifications, respectively, resulting in heterozygous IFNAR1^+/−^ and IFNLR1^+/−^ progeny. The modified alleles segregated in a Mendelian manner, and homozygous IFNAR1^−/−^ and IFNLR1^−/−^ lines were successfully established. TaqMan allelic discrimination assays were employed to genotype the IFNAR1 KO animals (Figure 1c), while a PCR genotyping assay was established for IFNLR1 KO chickens (Figure 1d). Phenotypically, both IFNAR1^−/−^ and IFNLR1^−/−^ chickens exhibited normal body weight gain compared to their WT counterparts (Figure 2a). Since both type I IFN and type III IFN play crucial roles in protecting the female reproductive tract against viral infections and maintaining immune homeostasis in mammals (Caine et al., 2019; Cumming and Bourke, 2019), we evaluated the fertility of IFNAR1^−/−^ and IFNLR1^−/−^ chickens by breeding homozygous birds from each line separately. The resulting F2 generation revealed normal egg production and fertility in healthy, unchallenged KO chickens (data not shown), consistent with studies in mice lacking either IFNAR1^−/−^ or IFNLR1^−/−^ (Ank et al., 2008; Müller et al., 1994).

### KO of IFNAR1 and IFNLR1 impairs IFN signaling and induction of antiviral state

Western blot analysis confirmed the functional loss of the IFNAR1 receptor in the IFNAR1^−/−^ chickens. Treatment with recombinant IFN-α resulted in the induction of IFN-stimulated protein Mx in WT and IFNAR1^+/−^ chicken embryo fibroblasts (CEFs) but not in the CEF derived from IFNAR1^−/−^ embryos (Figure 2b). In addition, treatment of both WT and IFNAR1^−/−^ embryos with recombinant IFN-α before WSN33 (H1N1) challenge resulted in a significant (p≤0.05) reduction of viral titers in the allantoic fluid of WT but not in IFNAR1^−/−^ embryos (Figure 2c). These results demonstrated that the signaling pathway mediated by IFNAR1 is nonfunctional in the absence of this receptor, thus validating the KO model. Additionally, RT-PCR analysis was conducted to assess the expression of IFNLR1 (IL-28Rα) across multiple tissues, including the spleen, liver, lung, intestine, bursa of Fabricius, heart, and cecum in WT, IFNLR1^+/−^, and IFNLR1^−/−^ embryos. The analysis revealed a complete absence of IFNLR1 mRNA in all IFNLR1^−/−^ tissues compared to detectable levels in WT and IFNLR1^+/−^ samples. This absence indicates the successful functional loss of IFNLR1 (Figure 2d).

### KO of IFNAR1 and IFNLR1 modulates the population of immune cells in the blood and spleen

**Figure 3.**
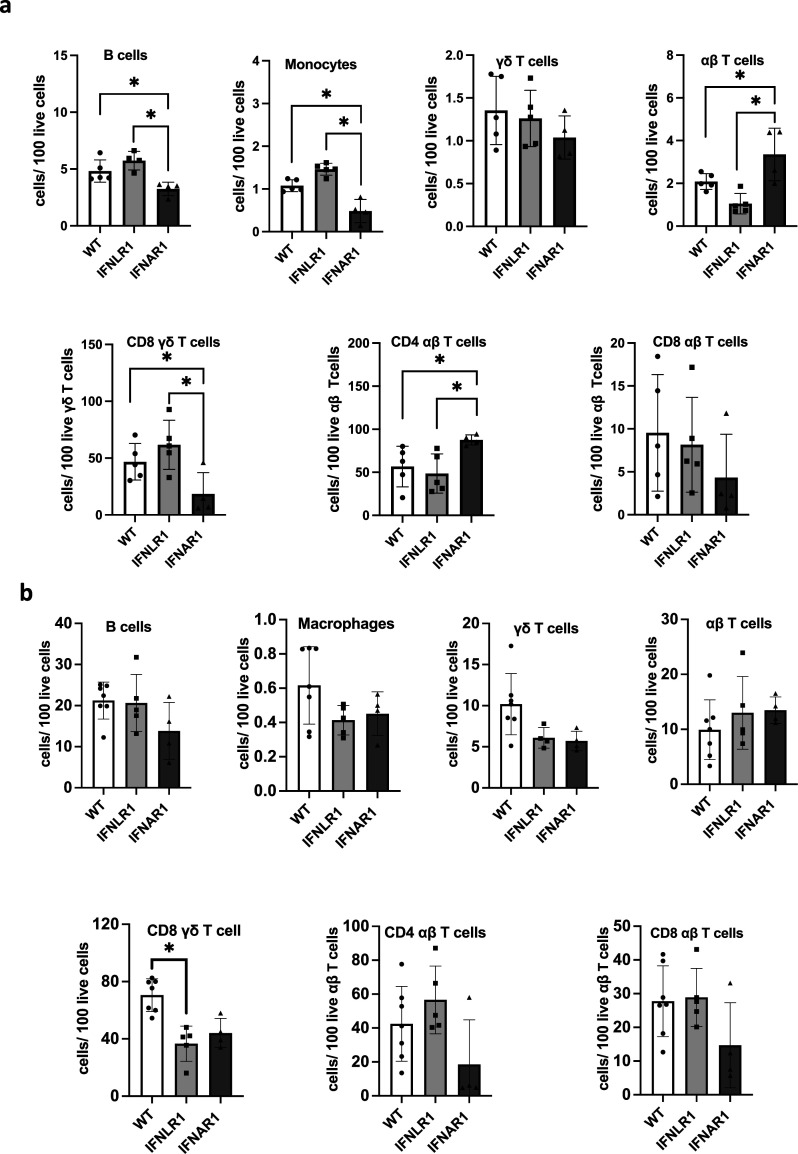
Flow cytometry analysis of peripheral blood mononuclear cells (PBMCs) and splenocytes of WT, IFNLR1^−/−^, and IFNAR1^−/−^ in 1-month-old chickens. (**a**) PBMCs and (**b**) splenocytes were isolated and analyzed for differential immune cell populations, including monocytes/macrophages, B cells, αβ TCR2,3+ or γδ TCR1+T cells, and associated CD4 and CD8 T cell subpopulations. Data are presented as mean ± SEM. One-way ANOVA was used to analyze the difference between the groups (*, p≤0.05). Number of animals for the analysis of PBMCs (WT = 5, IFNLR1^−/−^=5, IFNAR1^−/−^=4), and analysis of splenocytes (WT = 7, IFNLR1^−/−^=5, IFNAR1^−/−^=4).

Flow cytometry analysis demonstrated a significant (p≤0.05) reduction in the blood B cells and monocyte populations and an increase in αβ T cells in 1-month-old IFNAR1^−/−^ chickens compared to WT and IFNLR1^−/−^. Furthermore, there was a marked decrease in CD8^+^ γδ T cells, accompanied by a significant (p≤0.05) increase in CD4^+^ αβ T cells in the IFNAR1^−/−^ group compared to other groups (Figure 3, Figure 3—figure supplement 1). Although flow cytometry analysis did not reveal significant differences in the number of B cells, macrophages, αβ, and γδ T cells in the spleen of WT, IFNAR1^−/−^, and IFNLR1^−/−^, there was a considerable decrease in CD8^+^ γδ T cells in IFNLR1^−/−^ as compared to WT chicks (Figure 3). These findings highlight the differential impact of IFNAR1 and IFNLR1 KO on specific T cell subsets and other immune cell populations, and the important roles of these receptors in immune regulation.

**Figure 3—figure supplement 1.**
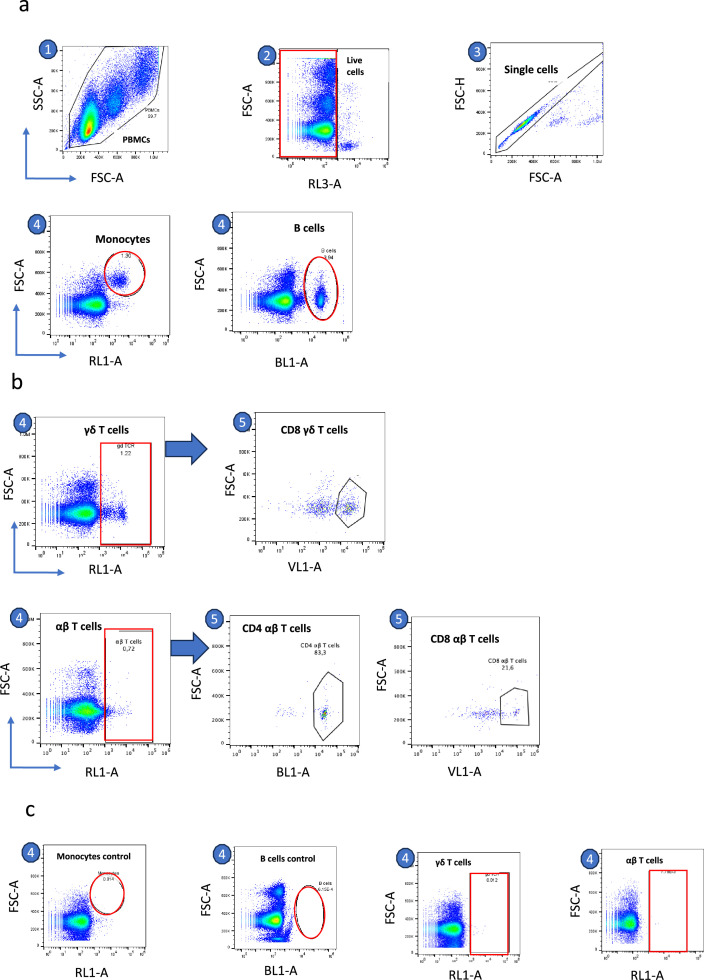
Representation of the gating strategy to quantify populations of peripheral blood mononuclear cells (PBMCs) isolated from 1-month-old chicks. Steps 1, 2, and 3 are common for all. All individual cell analyses were gated from step 3 except for the subpopulation of γδ and αβ T cells, which were gated from step 4. (**a**) Detection of monocytes and B cells. (**b**) Detection of γδ, αβ T cells, and their CD4 and CD8 subpopulations. (**c**) Secondary antibody control used for this experiment.

**Figure 3—figure supplement 2.**
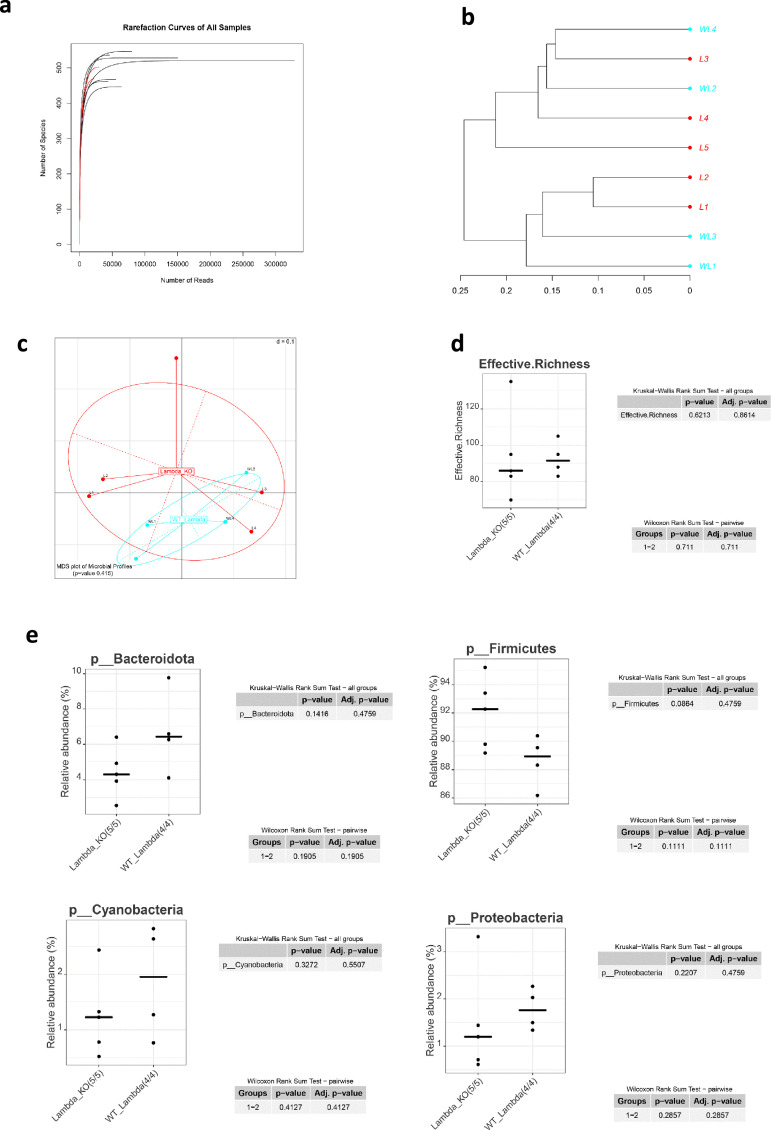
Microbiome analysis of the cecal contents from 1-month-old IFNLR1^−/−^ chicks and their WT siblings. (**a**) Rarefaction curves of all samples show that sequencing depth was sufficient. (**b**) A phylogram based on Ward’s minimum variance method shows the hierarchical clustering of samples. The sample distance is shown on the x-axis. The IFNLR1^−/−^ chicks are abbreviated as L, and their WT siblings as WL. (**c**) Multidimensional scaling plots of microbial profiles. Groups were compared pairwise. The scale bar indicates the distance between samples (d=0.1 describes 10%). (**d**) Richness as an index for α-diversity. Means are displayed as bold bars. (**e**) Taxonomic differences between the groups at the phylum level. Means are indicated as bold bars. The number at the bottom shows the number of observations in the groups.

### KO of IFNLR1 did not modulate the cecum microbiome

Given the high expression of IFNLR1 in epithelium-rich organs such as the intestine and considering the constant microbial exposure in this region, we investigated the impact of IFNLR1 KO on the cecum microbiomes of 1-month-old healthy chicks. While microbial sequencing disclosed distinct multidimensional scaling plots between the IFNLR1^−/−^ and their WT counterparts, subsequent alpha diversity analyses and taxonomic diversity did not demonstrate significant differences between the groups (Figure 3—figure supplement 2). However, it is noteworthy that this difference could be more pronounced during immune challenges when IFN-λ signaling is actively engaged.

### Reduced IgM and IgY production in IFNAR1^−/−^ and IFNLR1^−/−^ chickens following immunization

**Figure 4.**
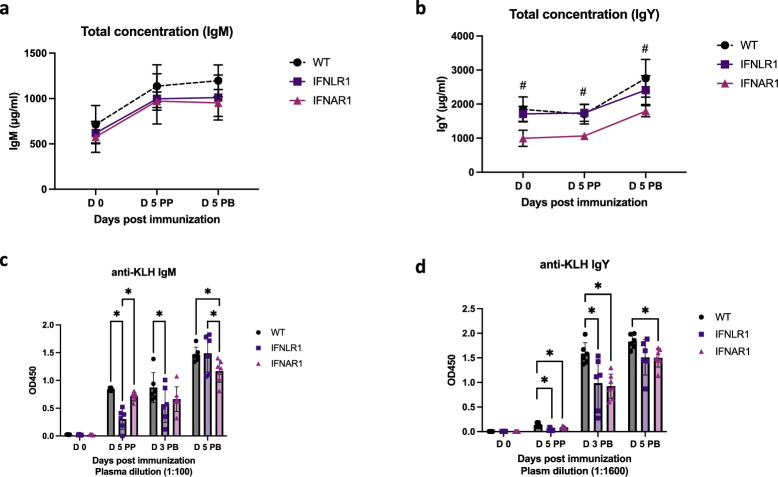
Reduced IFNLR1^−/−^ and IFNAR1^−/−^ chicken ability to produce IgM and IgY in response to immunization. Five-week-old chickens were immunized via intramuscular injection with 300 µg keyhole limpet hemocyanin (KLH), mixed 1:1 with incomplete Freund’s adjuvant. A booster injection of 300 µg KLH, mixed 1:1 with Freund’s incomplete adjuvant, was administered 2 weeks after the initial immunization. Blood plasma samples were collected from all groups before immunization (day 0), on day 5 post-primary immunization (PP), and on days 3 and 5 post-booster immunization (PB). (**a–d**) Total concentration of IgM, IgY, and KLH antigen-specific IgM and IgY levels were assessed using enzyme-linked immunosorbent assay (ELISA). Data are presented as mean ± SEM of at least 6 birds per genotype, with the same animals tracked over time. Statistical differences between groups are indicated by asterisks (*, p≤0.05). # Indicates significant difference (p≤0.05) for IFNAR1^−/−^ vs. WT and IFNLR1^−/−^.

To study the role of type I and type III IFNs in immune response and antibody production, 1-month-old chicks were immunized with keyhole limpet hemocyanin (KLH) (Figure 4—figure supplement 1). Analysis of total IgM and IgY concentrations in the plasma of preimmunized animals (day 0) revealed a significantly (p≤0.05) reduced IgY synthesis and secretion in IFNAR1^−/−^ chickens compared to the WT group. Furthermore, all groups showed significant (p≤0.05) increases in total IgM and IgY concentrations on day 5 after both primary and secondary immunizations compared to day 0. These elevations were solely attributed to immunization with KLH and FIA (Figure 4a and b). Analysis of KLH-specific IgM and IgY levels showed significantly (p≤0.05) lower levels of both antibodies in IFNAR1^−/−^ and IFNLR1^−/−^ chicks compared to the WT group. Notably, although IFNLR1^−/−^ chicks had significantly (p≤0.05) lower IgM and IgY levels than IFNAR1^−/−^ on day 5 post-primary immunization, they were able to reach immunoglobulin levels comparable to WT chicks by day 5 after booster immunization (Figure 4c and d). This indicates that IFN-λ contributes to the initial antibody production following immunization but can be compensated for by type I IFN during booster immunization.

**Figure 4—figure supplement 1.**
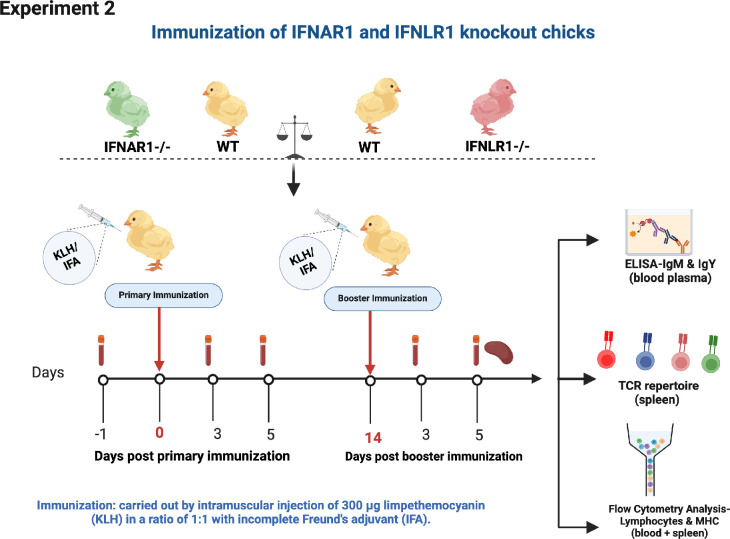
In experiment 2, 5-week-old WT, IFNLR1^−/−^, and IFNAR1^−/−^ chicks were immunized via intramuscular injection with 300 µg keyhole limpet hemocyanin (KLH), mixed 1:1 with incomplete Freund’s adjuvant. A booster injection of 300 µg KLH, mixed 1:1 with Freund’s incomplete adjuvant, was administered 2 weeks after the initial immunization. Plasma samples were collected from all groups before immunization (day 0), on day 5 post-primary immunization (PP), and on days 3 and 5 post-booster immunization (PB). The total concentration of IgM, IgY, and levels of KLH antigen-specific IgM and IgY were assessed using enzyme-linked immunosorbent assay (ELISA). Peripheral blood mononuclear cells (PBMCs) were isolated on day 3 post-PP, and splenocytes were isolated on day 5 PB for fluorescence-activated cell sorting (FACS) analysis of immune and MHC-positive cells. T cells of the spleen were used to study T cell receptor (TCR) repertoire. At least 6 animals per group were included.

**Figure 4—figure supplement 2.**
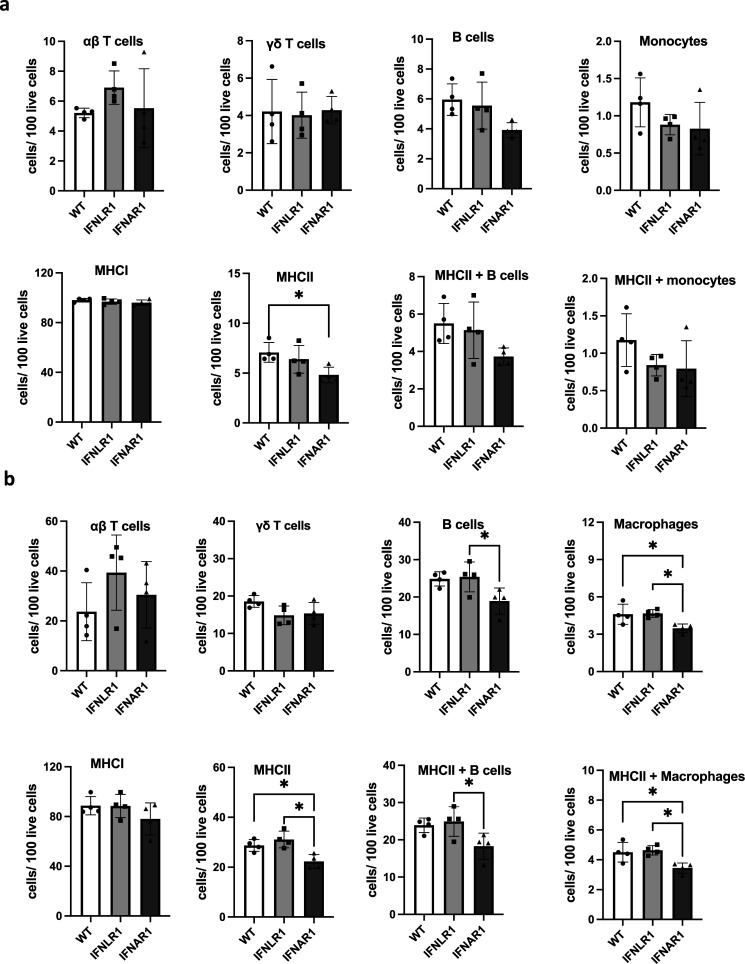
Flow cytometry analysis of immune cells and their MHC-positive subpopulations in the blood and spleen of WT, IFNLR1^−/−^, and IFNAR1^−/−^ chicks immunized with keyhole limpet hemocyanin (KLH). (**a**) Peripheral blood mononuclear cells (PBMCs) were isolated at day 3 post-primary immunization and analyzed for differential immune cell populations, including monocytes, B cells, αβ TCR2,3+ or γδ TCR1+T cells, MHCI, MHCII, MHCII+B cells, and MHCII+monocytes. (**b**) Splenocytes were isolated at day 5 post-booster immunization and analyzed for differential immune cell populations and associated MHC subpopulations. Data are presented as mean ± SEM of 6 birds per genotype. One-way ANOVA was used to analyze the difference between the groups (*, p≤0.05).

**Figure 4—figure supplement 3.**
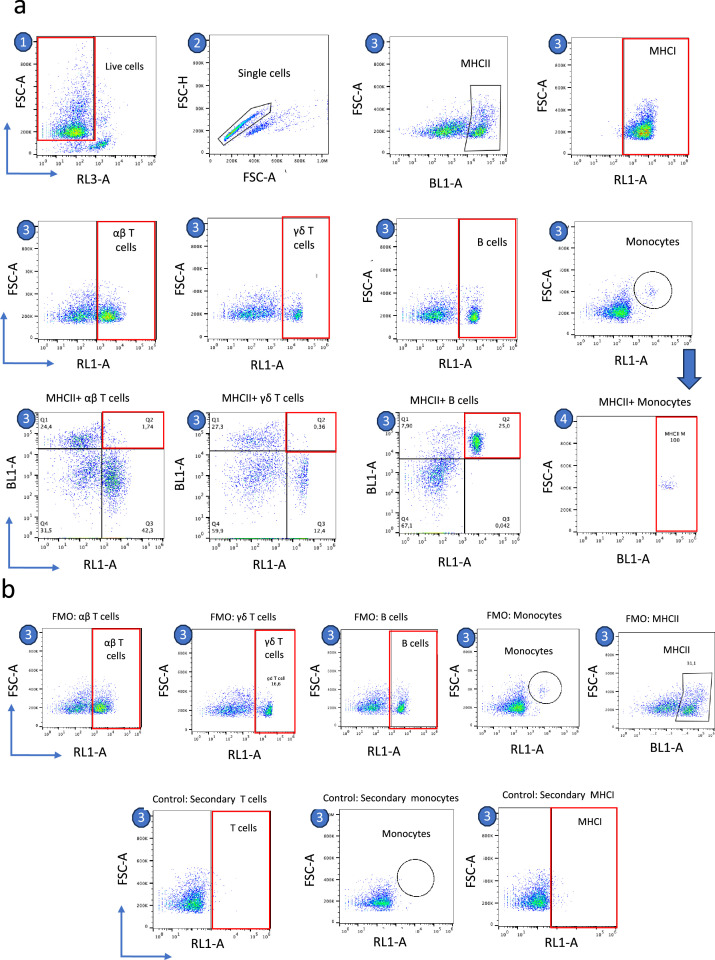
Representation of gating strategy quantifying immune cells and their MHC-positive subpopulations. Steps 1 and 2 are common for all. All individual cell analyses were gated from step 2 except for MHCII+monocytes. (**a**) Immune cells and their subpopulation positive for MHCII. (**b**) Fluorescence minus one (FMO) controls and secondary antibody control were used for this experiment.

### IFNAR1 KO reduces MHCII^+^ cells without altering MHCII expression

Analysis of splenocytes isolated on day 5 post-secondary immunization revealed a significant (p≤0.05) reduction in the frequency of MHCII^+^ cells in IFNAR1^−/−^ chickens compared to other genotypes. This decrease was particularly pronounced in specific immune cell subsets, notably MHCII^+^ B cells and MHCII^+^ macrophages, where the percentages of these cells were markedly lower in IFNAR1^−/−^ chickens (Figure 4—figure supplement 2, Figure 4—figure supplement 3). Interestingly, the mean fluorescence intensity of MHCII, which indicates the density of MHCII molecules on individual cells, remained consistent across all experimental groups (data not shown). This observation suggests that the observed reduction in MHCII^+^ cells is primarily due to a decrease in the overall populations of macrophages and B cells expressing MHCII rather than a diminished expression of MHCII per cell.

### Quantitative reduction in the response of TRBV2-1-expressing T cells in immunized IFNAR1^−/−^ animals

**Figure 5.**
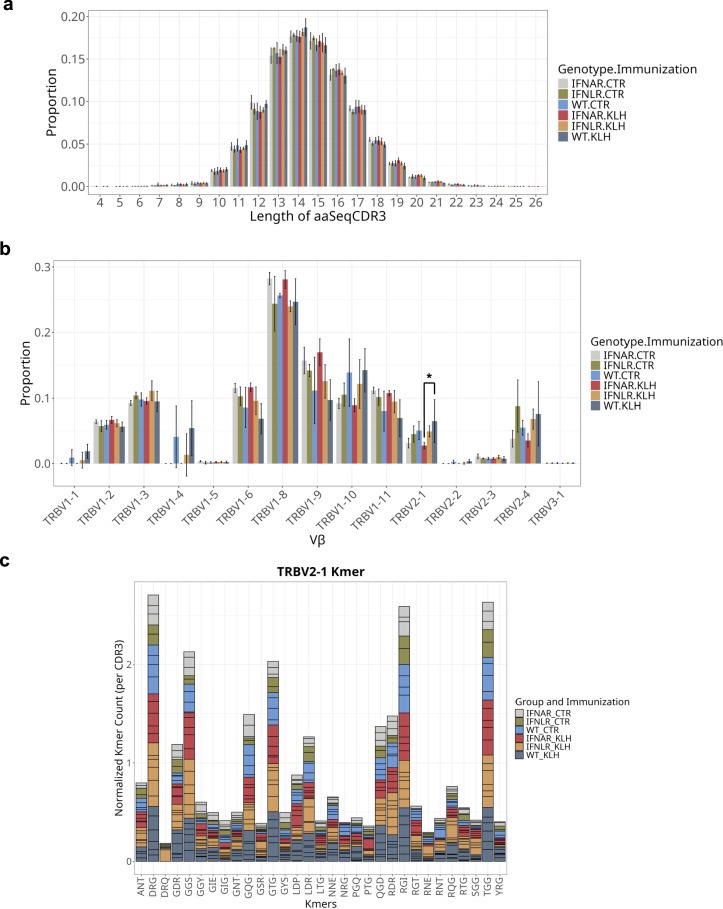
Overall similar splenic TCRβ repertoire across genotypes with reduced TRBV2-1 responses in keyhole limpet hemocyanin (KLH)-immunized IFNAR1^−/−^ chickens. TCRβ CDR3 clonotypes in IFNAR1^−/−^, IFNLR1^−/−^, and WT animals in the steady state (CTR) or at day 5 post-KLH booster immunization. (**a**) CDR3 spectratype; (**b**) Vβ gene usage; (**c**) counts of the 20 most prevalent 3-mers within the top 100 TRBV2-1 clonotypes for each sample, with individual samples indicated by black boxes. Data are presented as mean ± SD.

Next, we characterized the αβ T cell receptor (TCR) repertoire to assess whether T cell responses differ between IFNAR1^−/−^, IFNLR1^−/−^, and WT animals. Additionally, we aimed to determine if antibody responses to KLH are associated with clonal T cell responses, potentially indicating T-dependent antibody responses. Both α and β chains were sequenced from T cells derived from the spleen at day 5 post-booster immunization from all three groups. Unimmunized chickens from each genotype were included to establish a baseline for the expression of TCRs. Interestingly, the TCR repertoires across genotypes and immunization groups were remarkably similar for both α and β chains, as indicated by the pairing of V-J gene segments (Figure 5—figure supplement 1, Figure 5—figure supplement 2). This suggests that the global repertoires are consistent across different genotypes after immunization. Given the greater diversity of β chains compared to α chains, resulting from the recombination of V, D, and J gene segments, we focused on β chains for the subsequent analyses of CDR3 clonotypes. CDR3 spectratypes exhibited a Gaussian-like distribution in both control and immunized chickens across all genotypes, suggesting that KLH does not induce major clonal expansions in the splenic TCR repertoire 5 days after booster immunization (Figure 5a). This was confirmed by the analysis of clonotype frequencies, which predominantly consisted of rare clonotypes with only a few moderately expanded clonotypes observed in both control and immunized chickens (Figure 5—figure supplement 3). Although overall Vβ gene usage was similar in control and immunized chickens, TRBV2-1 was significantly less prevalent in KLH-immunized IFNAR1^−/−^ chickens than in WT immunized animals (Figure 5b). This suggests a reduced response of TRBV2-1-expressing T cells to KLH in IFNAR1-deficient chickens. To investigate whether specific amino acid motifs were enriched in KLH-immunized WT but not in IFNAR1^−/−^ chickens, we analyzed the counts of 3-mer sequence motifs in TRBV2-1 CDR3 clonotypes, normalized by the total number of TRBV2-1 CDR3 sequences in each sample. Overall, the most prevalent 3-mers in immunized WT chickens were also present in immunized IFNAR1^−/−^ chickens, indicating that TRBV2-1 clonotypes in IFNAR1^−/−^ chickens are qualitatively similar to those in WT chickens (Figure 5c). The observed differences are therefore more likely attributed to quantitatively reduced activation rather than distinct clonotype compositions.

**Figure 5—figure supplement 1.**
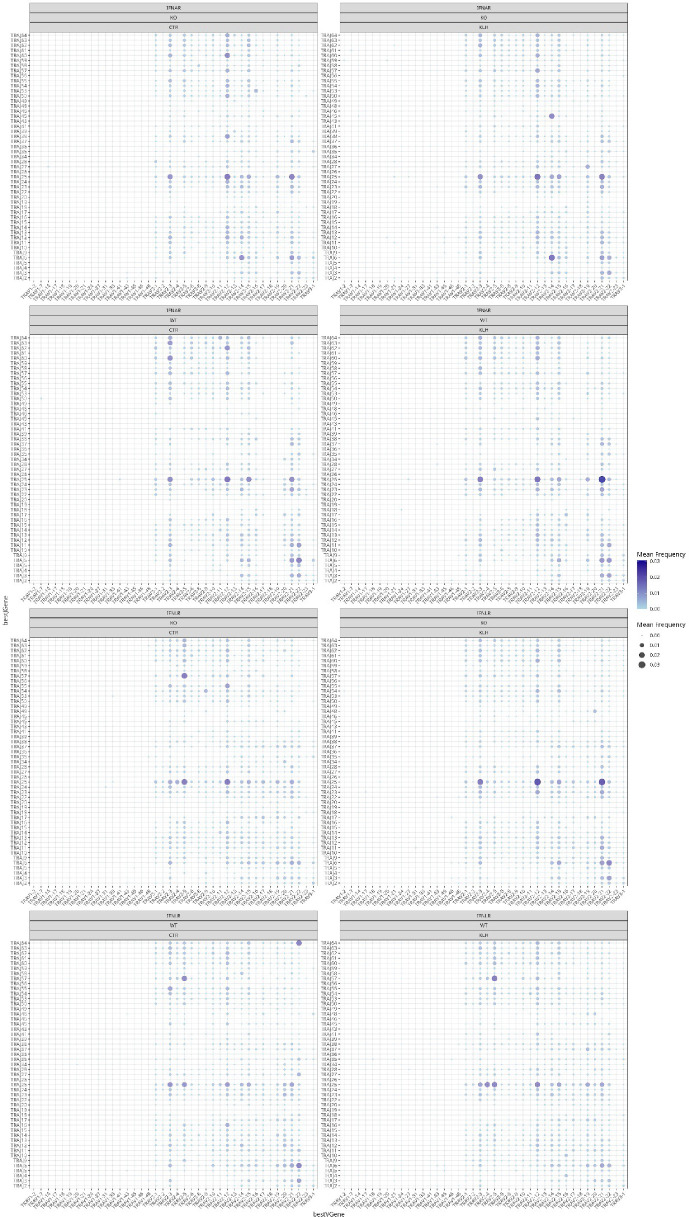
Relative frequency of Vα-Jα genes. Mean V-J pairing in TCRα CDR3s in IFNAR1^−/−^, IFNLR1^−/−^, and WT animals in the steady state (CTR) or at day 5 post-keyhole limpet hemocyanin (KLH) booster immunization. Data are presented as mean values.

**Figure 5—figure supplement 2.**
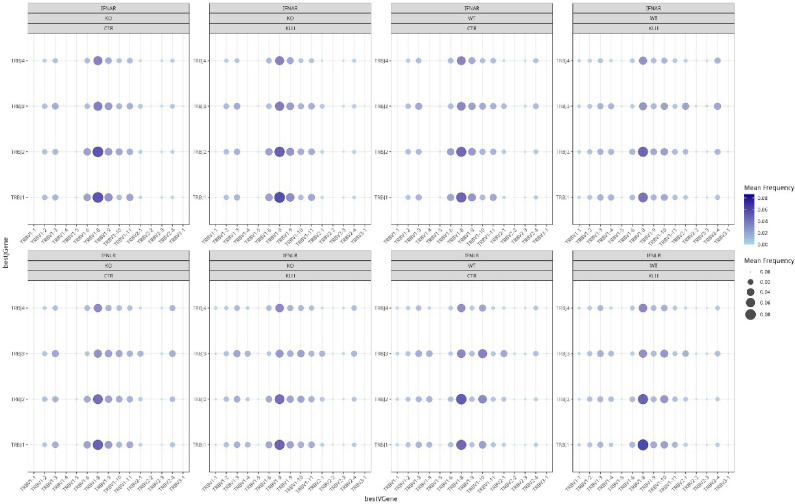
Relative frequency of Vβ-Jβ genes. Mean V-J pairing in TCRβ CDR3s in IFNAR1^−/−^, IFNLR1^−/−^, and WT animals in the steady state (CTR) or at day 5 post-keyhole limpet hemocyanin (KLH) booster immunization. Data are presented as mean values.

**Figure 5—figure supplement 3.**
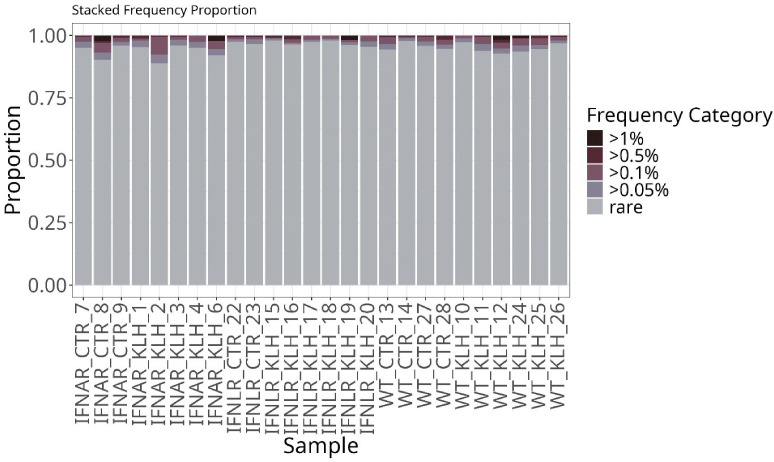
Relative frequency distribution of TCRβ CDR3s. Distribution of CDR3 clonotypes color-coded by their relative frequencies in IFNAR1^−/−^, IFNLR1^−/−^, and WT animals in the steady state (CTR) or at day 5 post-keyhole limpet hemocyanin (KLH) booster immunization.

### Virus species- and strain-specific IFN responses

**Figure 6.**
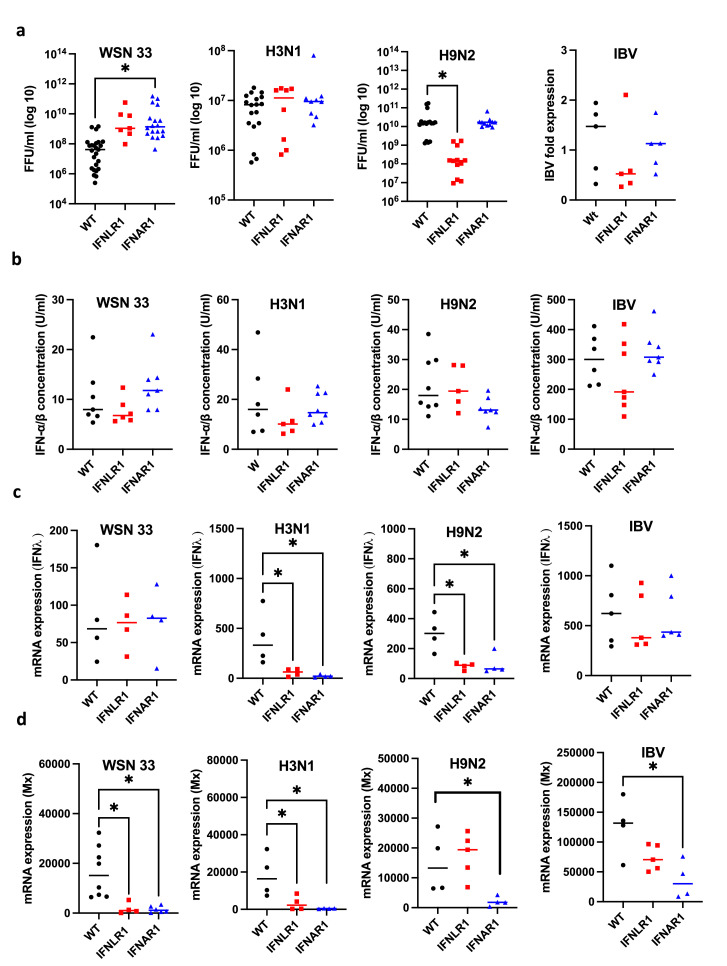
Viral titration, IFN-α/β concentration, IFN-λ, and Mx mRNA expression in WT, IFNLR1^−/−^, and IFNAR1^−/−^ embryos. (**a**) Embryonic day 11 chicken embryos were infected with 1000 FFU of influenza A virus strains (WSN33/H3N1/H9N2) and IBV-Beaudette strain, and allantoic fluid and chorioallantoic membrane (CAM) were sampled 24 hr post-infection. The viral titers of WSN33, H3N1, and H9N2 were quantified by titration on Madine-Darby canine kidney (MDCK) cells. For the IBV, qPCR was used to measure the viral titer in the CAM, and the housekeeping gene 28S was used to normalize target gene expression. (**b**) IFN-α/β concentration was quantified by titration of allantoic fluids on CEC-32 #511 cells. (**c**) Relative mRNA expression of IFN-λ in the CAM. (**d**) Relative mRNA expression of Mx in the CAM. For the qPCR study, RNA was isolated by TRIzol and reverse-transcribed with GoScript Transcription Mix and Random Primers, and qPCR was done via GoTaq. The WT control group was used as a calibrator for Mx and IFN-λ expression, and the housekeeping gene 18S was used to normalize target gene expression. Relative quantification of the target gene was performed using the 2^^ΔΔCT^ method. A no-template control was also included. Data are presented as mean ± SEM. Individual symbols represent individual embryos; the number of embryos varies by genotype and virus group and is shown by the individual symbols in each panel. Statistical differences between groups are indicated by asterisks (*, p≤0.05).

To investigate the role of type I and type III IFNs in the defense mechanisms against viral infections, an in ovo challenge experiment was performed using 11-day-old embryos (Figure 6—figure supplement 1). This experiment utilized four distinct viral strains: a laboratory-adapted human influenza A virus (H1N1), two low pathogenic avian influenza A virus strains (H3N1 and H9N2), and IBV-Beaudette strain, an important avian gamma coronavirus. A significant (p≤0.05) increase in WSN33 (H1N1) viral titers in the allantoic fluid was observed in IFNAR1^−/−^ and IFNLR1^−/−^ embryos compared to WT embryos, indicating a crucial role for both type I and type III IFN signaling in restricting WSN33 replication. Conversely, H9N2 viral titers were significantly (p≤0.05) reduced in IFNLR1^−/−^ embryos compared to IFNAR1^−/−^ and WT embryos, indicating a predominant role for type I IFN signaling in limiting H9N2 replication. However, no significant differences in viral titers were observed for H3N1 and IBV among the genotypes, suggesting potential strain-specific or virus-specific mechanisms governing the antiviral IFN response (Figure 6a).

**Figure 6—figure supplement 1.**
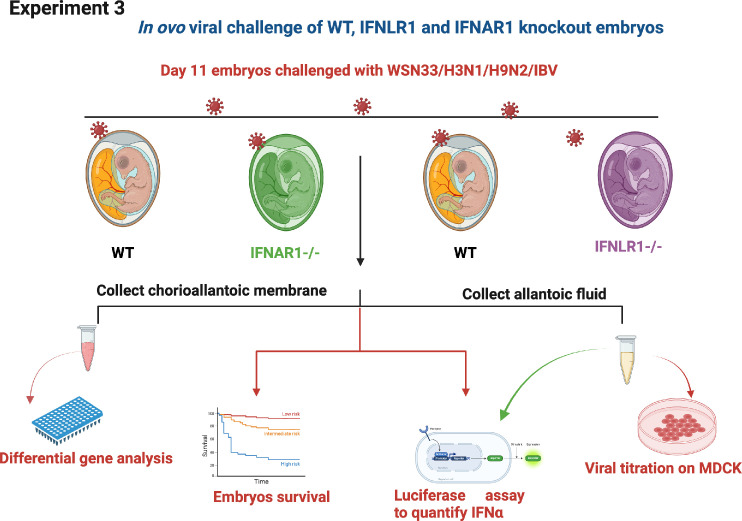
Design of experiment 3, 11-day-old embryos were infected with 1000 FFU of four distinct viral strains: a laboratory-adapted human influenza A virus (H1N1), two low pathogenic avian influenza A virus strains (H3N1 and H9N2), and infectious bronchitis virus (IBV)-Beaudette strain virus. Allantoic fluid and chorioallantoic membrane (CAM) were sampled 24 hr post-infection. The experiment was performed blindly, and the genotype of embryos was determined, and only WT and homozygous embryos were selected for further analysis. The viral titers of WSN33, H3N1, and H9N2 were quantified by titration on Madine-Darby canine kidney (MDCK) cells. For the IBV, qPCR was used to measure the viral titer in the CAM. Relative mRNA expression of Mx and IFN-λ were studied in the CAM using qPCR. IFN-α/β concentration was quantified by titration of allantoic fluids on CEC-32 #511 cells. The probability of survival in the challenged groups was also investigated.

**Figure 6—figure supplement 2.**
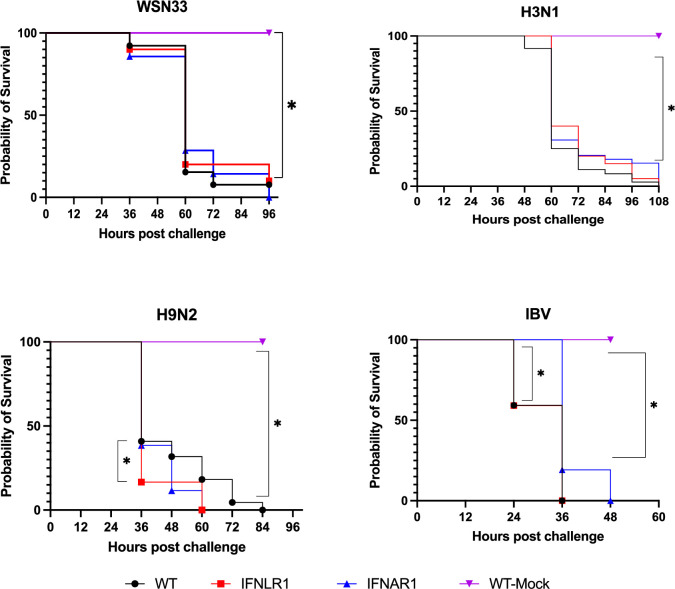
The probability of survival in WT, IFNLR1^−/−^, and IFNAR1^−/−^ embryos. On embryonic day 11, chicken embryos were infected with 1000 FFU of influenza A virus strains (WSN33/H3N1/H9N2) and infectious bronchitis virus (IBV)-Beaudette strain, and the probability of survival in the challenged groups was recorded. Statistical differences between groups are indicated by asterisks (*, p≤0.05).

### KO of IFNAR1 and IFNLR1 did not affect IFN-α/β secretion in ovo

The initial virus-host interaction and IFN secretion occur independently of IFNAR1 and IFNLR1 receptors. However, subsequent IFN production is regulated through a feedback mechanism mediated by these receptors. Indeed, no difference in the concentration of IFN-α/β in the allantoic fluid of WT, IFNAR1^−/^,^−^ and IFNLR1^−/−^ embryos was observed in response to viral infections in ovo (Figure 6b). IFNλ mRNA expression in the chorioallantoic membrane (CAM) showed a significant (p≤0.05) reduction in IFNAR1^−/−^ and IFNLR1^−/−^ compared to WT embryos challenged with low pathogenic avian influenza strains (Figure 6c). Interestingly, embryos infected with IBV-Beaudette strain showed at least 10-fold higher induction of IFN-α/β secretion compared to the influenza strains used in this experiment (Figure 6b).

### A predominant role of type I IFN in inducing the expression of an ISG (Mx)

Mx expression in the CAM was studied to elucidate the contributions of type I and type III IFNs in inducing ISGs during viral infections. The CAM of chicken embryos was used as a model due to its high vascularization and functional similarity to avian respiratory and immunological tissues. CAMs isolated from IFNAR1^−/−^ embryos 24 hr post-infection exhibited a significant decrease (p≤0.05) in Mx expression compared to WT embryos in all the tested viruses. In IFNLR1^−/−^ embryos, Mx levels in CAMs challenged with H9N2 and IBV-Beaudette strain remained comparable to Mx expression levels observed in WT embryos. These findings suggest a predominant role for type I IFN signaling in inducing the expression of ISGs, such as Mx, in response to H9N2 and IBV-Beaudette strain infections (Figure 6d).

### An excessive and unbalanced type I IFN response can harm host fitness

Survival probability experiments revealed that an overly strong IFN-α/β response may negatively impact host fitness more than the viral load. Consistent with this observation, measured IFN-α/β concentrations (Figure 6b) were elevated in these embryos, supporting the link between high type I IFN responses and reduced survival. This was evident in IFNLR1^−/−^ embryos challenged with H9N2, which exhibited poor survival rates compared to WT and IFNAR1^−/−^ embryos despite having low viral titers. Similarly, the heightened type I IFN response likely explains the rapid death of IBV-challenged embryos within 24–36 hr post-infection. In contrast, embryos infected with influenza strains exhibited approximately tenfold lower IFN-α/β concentrations compared to IBV-challenged embryos and survived longer, lasting 4–5 days after infection (Figure 6—figure supplement 2). These results underscore the critical yet potentially dual role of the type I IFN response in controlling viral infection, as it may also contribute to immunopathology.

### Early appearance of symptoms and poor survival of IFNAR1^−/−^ hens challenged with H3N1

**Figure 7.**
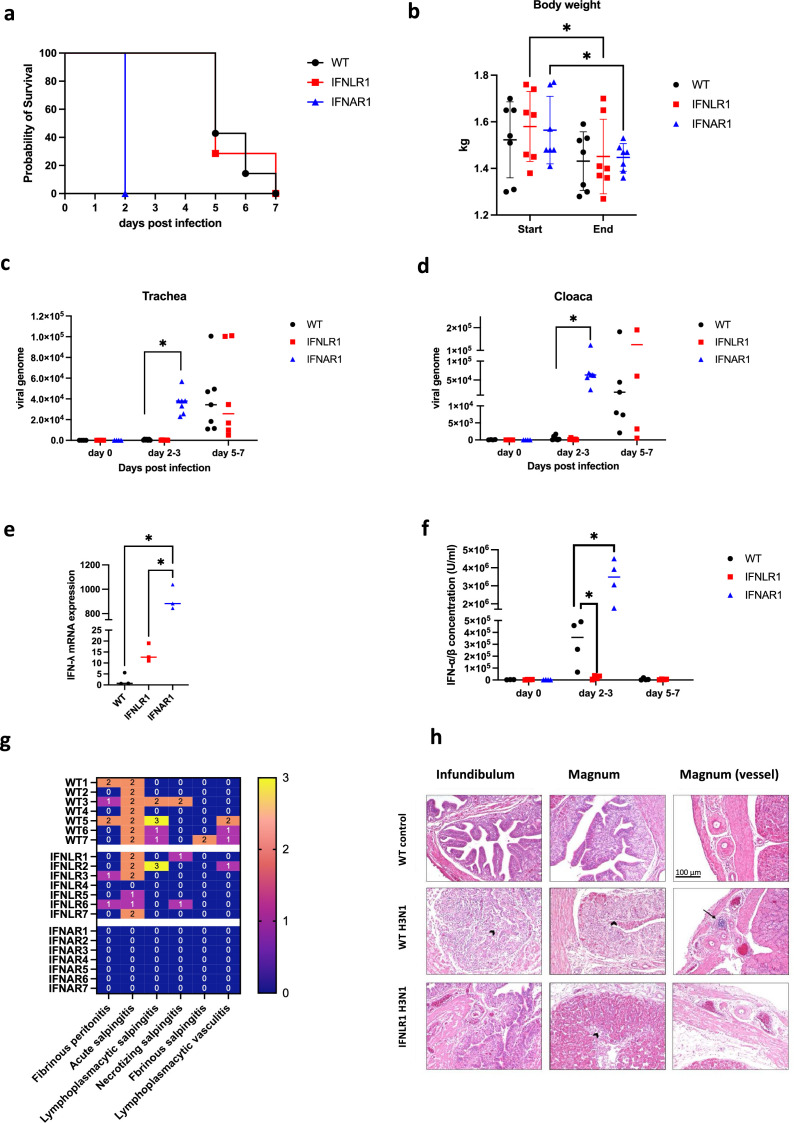
In vivo challenge of WT, IFNLR1^−/−^, and IFNAR1 ^−/−^ hens with a low pathogenic avian influenza A virus (H3N1). (**a**) Probability of survival in the challenged hens. (**b**) Chickens' body weight (kg) was measured before the viral challenge and at the end of the experiment (date of animal euthanization). (**c–d**) H3N1 titer in the tracheal and cloacal swabs using qPCR. The IFNAR1^−/−^ hens were sampled on day 2 post-infection. WT and IFNLR1^−/−^ hens were sampled on day 3 and days 5–7 post-infection based on the days of euthanization. The housekeeping gene 28S was used to normalize target gene expression. (**e**) Relative mRNA expression of IFN-λ in the spleen. Comparisons were designed to represent equivalent phases of disease progression. For panel e, IFNAR1^−/−^ hens at day 2 (high viral titer) were compared to WT and IFNLR1^−/−^ hens at day 5 (comparable high titer). (**f**) Plasma IFN-α/β concentration was quantified by titration on CEC-32 #511 cells. For panel f, IFNAR1^−/−^ hens (day 2, high titer) were compared to WT and IFNLR1^−/−^ hens at day 3 (low titer) and days 5–7 (high titer), reflecting distinct infection phases. (**g**) Scoring of lesions in the reproductive tract in all in vivo H3N1 challenged groups. The scoring is on a scale of 0–3, where 0 indicates normal and 3 maximum severity regarding lymphoplasmacytic salpingitis with mucosa atrophy. The IFNAR1^−/−^ hens were euthanized and scored on day 2 post-infection. WT and IFNLR1^−/−^ hens were euthanized and scored on days 5–7 post-infection based on the days of euthanization. (**h**) Histological assessment of the reproductive tract on day 5 post-infection. WT control: normal histological appearance of the infundibulum and magnum; WT H3N1: moderate acute inflammation of the infundibulum and magnum with hyperemia, edema, loss of epithelial cells (arrowheads), and perivascular infiltration of lymphocytes (arrow); IFNLR1^−/−^: milder acute inflammation of the infundibulum and magnum with hyperemia, edema, and loss of epithelial cells in the magnum (arrowhead). Hematoxylin-eosin (HE) stain; original magnification, ×100. Scale bars, 100 µm. For panels with error bars, data are presented as mean ± SEM. Seven infected hens and three non-infected control hens were included per genotype. Individual symbols represent individual hens sampled at the indicated time points. Statistical differences between groups are indicated by asterisks (*, p≤0.05).

To gain deeper insight into the role of IFN-α/β and IFN-λ in the defense mechanism against low pathogenic avian influenza A virus strains (H3N1), an in vivo infection experiment was carried out (Figure 7—figure supplement 1). IFNAR1^−/−^ hens exhibited a rapid onset of severe symptoms at 48 hr post H3N1 infection, including conjunctivitis, diarrhea, mucus discharge, inflamed, red eyes, abnormal posture, and severely restricted movement compared to WT and IFNLR1^−/−^ hens, which only showed similar symptoms from day 5 onward. All IFNAR1^−/−^ hens (n=7) were euthanized by day 2 post-infection after reaching the humane endpoint to minimize suffering. In comparison, four WT hens were euthanized on day 5, two on day 6, and one on day 7 post-infection. Similarly, five IFNLR1^−/−^ hens were euthanized on day 6 and two on day 7 post-infection (Figure 7a). Because humane endpoints were reached at different days for each genotype, body weight changes were analyzed within genotypes, and data are presented as mean ± SD (Figure 7b). There was a significant reduction (p≤0.05) in the body weight of IFNAR1^−/−^ and IFNLR1^−/−^ hens from the start to the end of the experiment. In contrast, while WT hens also showed a reduction in body weight by the end of the experiment, the change was not statistically significant (Figure 7b).

**Figure 7—figure supplement 1.**
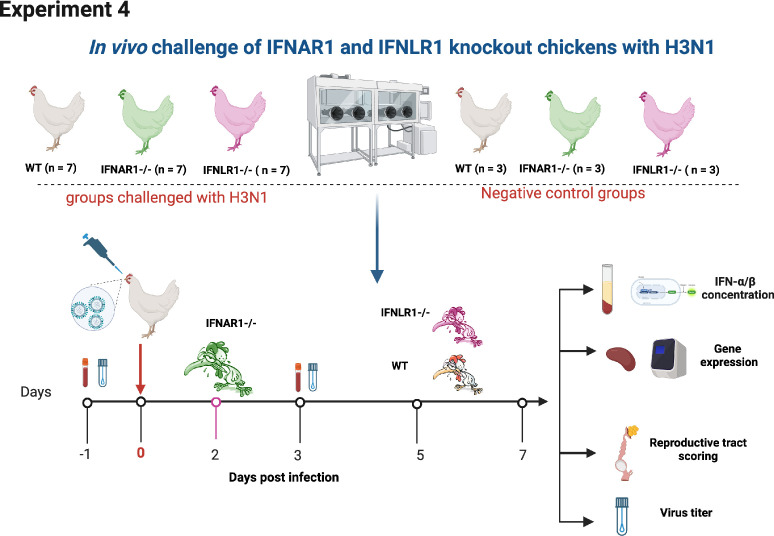
In experiment 4, 27-week-old WT, IFNLR1^−/−^, and IFNAR1^−/−^ hens were challenged with H3N1 avian influenza (10^6^ FFU) in 0.2 ml PBS per bird and distributed via nasal and tracheal routes. Seven birds were used for the infection experiment, and three others were used as negative controls per group. The IFNAR1^−/−^ hens showed severe sickness symptoms and were euthanized at day 2 post-infection, whereas WT, IFNLR1^−/−^ hens showed similar symptoms and were euthanized in the period between days 5 and 7 post-infection. Birds were monitored daily for clinical symptoms. Tracheal and cloacal swabs were collected to analyze the viral RNA loads by qPCR. Plasma was collected to estimate IFN-α/β concentration through luciferase assay. The spleen was collected for an immune-related gene expression study through qPCR. The female reproductive scoring was also studied to evaluate the impact of type I and type III IFNs on H3N1 pathogenesis.

### Efficient H3N1 replication in IFNAR1^−/−^ hens

In IFNAR1^−/−^ hens, the H3N1 virus titer increased significantly (p≤0.05) by day 2 post-infection in both the trachea and cloaca, indicating the limited ability of the immune system to restrict viral replications in these birds. In contrast, WT and IFNLR1^−/−^ hens maintained low H3N1 titers until day 5 post-infection, with levels rising similar to those in IFNAR1^−/−^ hens (Figure 7c and d).

### Increased plasma concentration of IFN-α/β and spleen expression of IFN-λ in IFNAR1^−/−^ hens

In IFNAR1^−/−^ hens, we observed an increase (p≤0.05) in the mRNA expression of IFN-λ, suggesting a compensatory mechanism in the absence of type I IFN signaling in the spleen where IFN-λ receptors are expressed (Figure 7e). For Figure 7e, comparisons were designed to represent equivalent phases of disease progression: IFNAR1^−/−^ spleens at day 2 post-infection (high viral titer) were compared with WT and IFNLR1^−/−^ spleens at day 5 post-infection, when viral titers were comparably high. In IFNAR1^−/−^ hens, the concentration of IFN-α/β showed around 10 times higher (p≤0.05) levels than in WT and IFNLR1^−/−^ hens at day 2–3 post-infection, indicating uncontrolled secretion of IFN-α/β in IFNAR1^−/−^ hens (Figure 7f). For Figure 7f, IFNAR1^−/−^ hens at day 2 (high titer) were compared with WT and IFNLR1^−/−^ hens at day 3 (low titer) and days 5–7 (high titer), thereby sampling corresponding disease stages across genotypes.

### IFN-λ signaling promotes oviduct inflammation in H3N1-infected chickens

The H3N1 avian influenza A virus demonstrates a distinct tropism for the hen’s oviduct, causing salpingitis and peritonitis associated with a severe decline in egg production. While young birds showed minimal clinical signs, adult layers exhibited higher mortality and decreased egg production in the second week post-infection (de Wit et al., 2020). To evaluate the impact of type I and type III IFN on the pathogenesis of H3N1, we evaluated oviduct inflammation, the site where H3N1 establishes the infection. The oviduct inflammation was scored on a magnum grade scale of 0–3 (0 indicating an intact oviduct and 3 indicating severe inflammation) (Sid, 2025). The results revealed a magnum grade 0 for the oviduct collected from IFNAR1^−/−^ at day 2 post-infection. The IFNLR1^−/−^ hens predominantly exhibited magnum grade 1, indicating mild lymphoplasmacellular salpingitis with normal mucosal height. In contrast, WT hens mostly displayed magnum grade 2, suggesting moderate lymphoplasmacellular salpingitis with prominent atrophy of the mucosa (Figure 7g). Histological analysis of the oviduct on day 5 post-infection revealed moderate acute inflammation in the infundibulum and magnum with hyperemia, edema, epithelial cell loss, and perivascular infiltration of lymphocytes in WT hens challenged with H3N1 compared to WT negative controls. In contrast, IFNLR1^−/−^ hens displayed milder inflammation in these parts of the oviduct (Figure 7h). These results indicate a pro-inflammatory role of IFN-λ in H3N1 infection in the oviduct.

### Increased expression of pro-inflammatory cytokines in the spleen of IFNAR1^−/−^ hens

In spleens sampled 2 days after infection, IFNAR1^−/−^ hens showed a significant (p≤0.05) increase in the mRNA expression of several pro-inflammatory cytokines, including IL-1β, IL-2, IL-6, IL-8, IL-12, and IL-22. This increase was observed compared to both WT and IFNLR1^−/−^ hens, whose spleens were sampled 5 days post-infection. Notably, IL-17A expression did not show a significant difference between the groups (Figure 8—figure supplement 1). The elevated levels of IL-2 and IL-12 point to the activation of the Th1 pathway (Kaiser, 2010). The upregulation of IL-1 and IL-6 suggests an induction of the Th22 pathway, known to stimulate Th22 cells, a recently characterized CD4^+^ T cell lineage, to release IL-22 but not IL-17A (Bean and Lowenthal, 2022; Eyerich et al., 2009). This would lead to inflammation and tissue damage as overexpression of IL-22 is associated with inflammatory tissue pathology (Dudakov et al., 2015). Also, there was no change in the expression of genes associated with the anti-inflammatory pathway and regulatory T cells (Tregs) (FOXP3, IL-10, TGF-β), indicating a lack of a compensatory anti-inflammatory mechanism to control the inflammation in IFNAR1^−/−^ hens. Additionally, there was a significant (p≤0.05) decrease in the expression of IL-4 and IL-5 in IFNAR1^−/−^ hens, which indicates lower Th2 pathway activity in these hens (Figure 8—figure supplement 1).

### Impairment of negative feedback mechanisms regulating IFN-α/β secretion and low antiviral state in the spleen of IFNAR1^−/−^ hens

**Figure 8.**
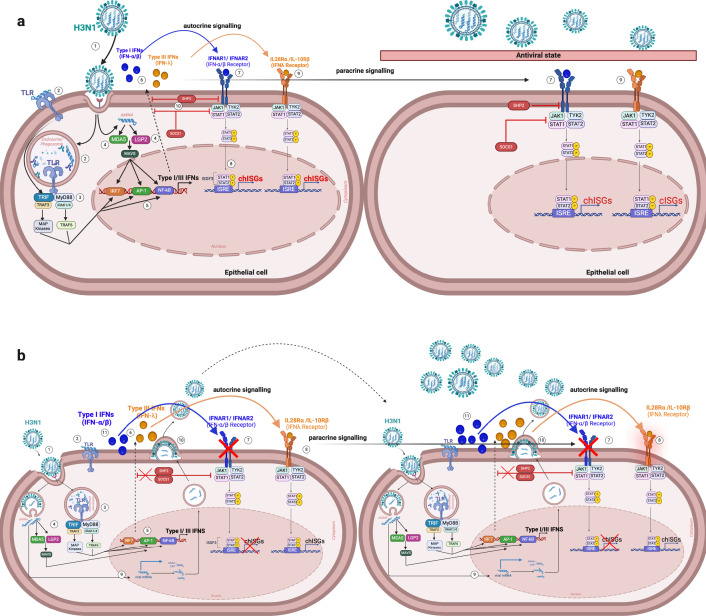
Schematic representation of H3N1 host cell interaction and interferon (IFN) signaling pathway in WT and IFNAR1^−/−^ at day 2 post-H3N1 challenge. (**a**) Immune signaling pathway in WT epithelial cells during an H3N1 viral infection. The cleaved hemagglutinin (HA) proteins of H3N1 bind to sialic acid receptors on the surface of epithelial cells, facilitating viral entry through endocytosis (1). The viral RNA inside the endosome is recognized by Toll-like receptors (TLRs) (2), leading to the activation of the MYD88 signaling pathway (3) and melanoma differentiation-associated protein 5 (MDA5) and/or laboratory of genetics and physiology 2 (LGP2) (4). In the MDA5/LGP2 pathway, mitochondrial antiviral-signaling proteins (MAVS) are activated. The activation of MYD88 leads to the recruitment of IRAK1/4 and TRAF6/3, which then stimulate the transcription factors nuclear factor kappa-light-chain-enhancer of activated B cells (NF-κB), interferon regulatory factor 7 (IRF7), and activating protein 1 (AP-1) (5). These transcription factors induce the production of pro-inflammatory cytokines and type I and type III IFNs (6). Type I IFN (IFN-α/β) binds to its receptors (IFNAR1/2) by autocrine and paracrine signaling (7). This binding activates the JAK-STAT pathway, leading to the phosphorylation of signal transducers and activators of transcription (STATs). These STATs translocate to the nucleus and activate the transcription of interferon-stimulated response elements (ISREs). The activation of ISREs drives the expression of interferon-stimulated genes (ISGs), establishing a robust antiviral state that effectively halts viral replication and spread within the cell (8). Similarly, type III IFN (IFN-λ) contributes to the antiviral state by binding to its receptors (IL-28Rα /IL-10Rβ) and activating the JAK-STAT pathway in an autocrine or paracrine manner (9). The immune response is tightly regulated by negative feedback mechanisms, including suppressor of cytokine signaling (SOCS1) and Src Homology 2-containing Phosphatase 2 (SHP2), which inhibit further production of IFNs and prevent excessive inflammatory responses (10). This response ensures that any initial viral replication is quickly suppressed, protecting the cell from significant infection and excessive immune responses that could lead to cytokine storm. The viral replication is significantly halted since the cells are in an antiviral state. (**b**) Immune signaling pathway in IFNAR1^−/−^ epithelial cells. Virus entry to the cell, recognition of viral RNA by the host cell, and induction of pro-inflammatory cytokines and IFNs happen in a mechanism similar to the WT cell (steps 1–6). However, without IFNAR1, IFN-α/β cannot bind to its receptors, disrupting downstream signaling and feedback inhibition, leading to unregulated IFN production and poor antiviral state (7). Higher IFN-λ is produced to compensate for the low antiviral state but cannot compensate for the absence of IFN-α/β (8). This makes the infected and neighboring cells easy targets for the H3N1 virus that can replicate quickly without resistance. Once inside the cell in the endosome, the viral RNA is released and transported into the nucleus for replication and transcription (9). Newly formed viral particles assemble, bud off, and are released to infect additional cells (10). In the absence of IFNAR1 and an antiviral state, the autocrine feedback loop mediated by SOCS1 and SHP2 is disrupted, and thus, the production of IFN-α/β is continuously increasing (11). The extensive pro-inflammatory cytokines and IFN production exacerbate inflammatory responses and cell death.

A gene expression study was performed on the spleen to gain deeper insights into the alterations in H3N1-host cell interactions and modulation of IFN signaling in IFNAR1^−/−^ hens. mRNA expression analysis of genes related to H3N1-host cell interaction, pro-inflammatory cytokines, and IFN induction (TLR3, MDA5, MyD88, NFkB, and IRF7) showed no significant differences between the groups (data not shown), suggesting that these pathways function normally in both WT and IFNAR1^−/−^ cells (Figure 8a and b). This could also be attributed to the fact that the spleen used in this analysis was collected from WT and IFNLR1^−/−^ at day 5 post-infection, a time when the viral load was high in these animals, and this pathway was active, similar to the IFNAR1^−/−^ hens. Viral sensation by the host cell and activation of IFN-α/β secretions occur in various cell types expressing TLRs, including immune cells, epithelial cells, fibroblasts, and endothelial cells (Katze et al., 2002; Röll et al., 2017). The interaction with the IFNAR1 receptor is needed for the secreted IFN-α/β to mediate its function. The KO of IFNAR1 led to a significant decrease in this group’s antiviral state. Relative mRNA expression of antiviral genes induced by IFN, including viperin, ISG12, IFITM5, PKR, and STAT1, reflects significantly (p≤0.05) lower antiviral state in the IFNAR1^−/−^ group as compared to WT and IFNLR1^−/−^ hens. These results indicate that IFN-α/β can compensate IFN-λ to induce an adequate antiviral state during H3N1 infection. However, IFN-λ cannot compensate for IFN-α/β in the spleen (Figure 8—figure supplement 1). In WT cells, negative feedback mechanisms mediated by suppressor of cytokine signaling 1 (SOCS1) and Src Homology 2-containing Phosphatase 2 (SHP2) regulate the secretion of IFN and associated inflammation once an adequate antiviral state is achieved. In IFNAR1^−/−^ cells, a low antiviral state and high viral titers likely prevent the efficient activation of the negative feedback loop, as indicated by the reduced expression of SOCS1 and SHP2 (Figure 8—figure supplement 1). Consequently, this leads to the continuous and significant secretion of IFN-α/β (Figure 8).

**Figure 8—figure supplement 1.**
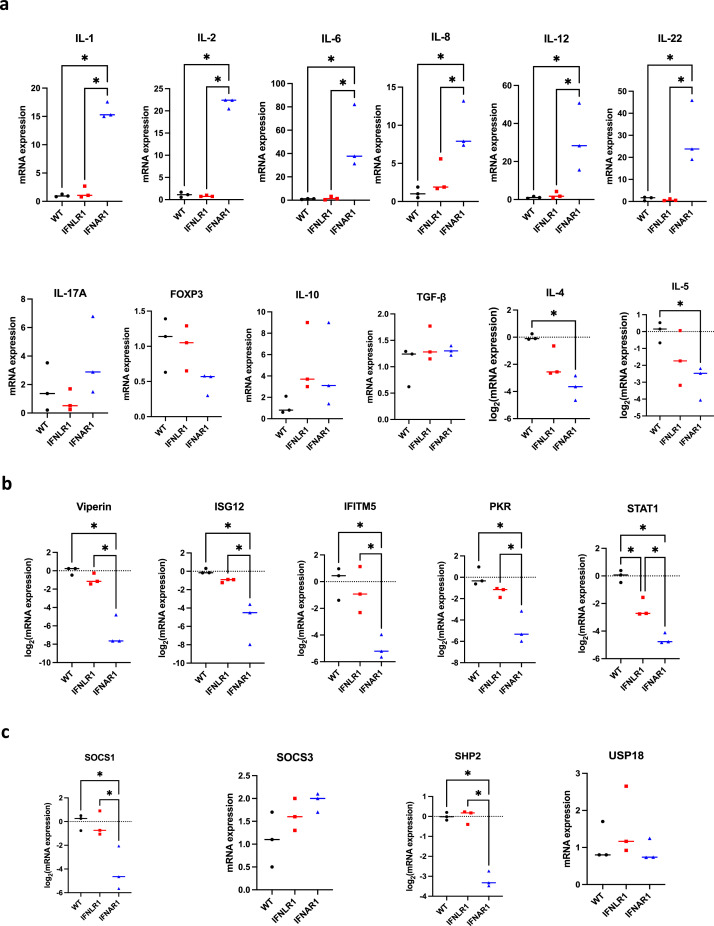
Relative mRNA expression of various pro- and anti-inflammatory cytokines (**a**), interferon-stimulated genes (**b**), and interferon signaling inhibitors (**c**) in the spleen of H3N1 challenged hens. For the qPCR study, RNA was isolated by TRIzol and reverse-transcribed with GoScript, and qPCR was done via GoTaq. The WT control group was used as a calibrator for the expression of all the studied genes, and the housekeeping gene 18S was used to normalize target gene expression. Relative quantification of the studied gene was performed using the 2^^ΔΔCT^ method. A no-template control was also included. The IFNAR1^−/−^ hens were euthanized on day 2 post-infection. WT and IFNLR1^−/−^ hens were euthanized on days 5–7 post-infection based on the days of euthanization. Only spleen samples collected on day 5 post-infection were included for WT and IFNLR1^−/−^ hens for the gene expression study. Data are presented as mean ± SEM; n=3 hens per genotype. Individual symbols represent individual hens. Statistical differences between groups are indicated by asterisks (*, p≤0.05).

## Discussion

IFNs are crucial components of the innate immune system, playing a pivotal role in host defense against viral, bacterial, and other pathogenic insults. In mammals, the potent antiviral activities induced by IFNs have rendered them attractive therapeutic agents for bolstering the immune response against infections and diseases (Galani et al., 2021; Hemann et al., 2019; Santer et al., 2022). Notably, both IFN-α/β and IFN-λ have demonstrated potential as adjuvants for enhancing the efficacy of mucosal vaccines, particularly against respiratory viruses such as influenza, in murine models (Bracci et al., 2005; Ye et al., 2021). Developing novel therapeutic approaches to combat and eradicate viral and bacterial infections in poultry necessitates a comprehensive understanding of the mechanisms of action of type I and type III IFNs. To investigate this further, we have generated type I and type III IFN receptors KO chickens to elucidate their potential roles in controlling avian influenza and other avian viral diseases. The IFNAR1^−/−^ and IFNLR1^−/−^ chickens exhibited normal growth and development compared to their WT counterparts. Flow cytometry analysis of the immune cell population in blood, spleen, and plasma concentrations of immunoglobulins indicate that IFN-α/β plays a crucial role in regulating innate immune cells, T cell subsets, and their contribution to antibody production under homeostatic conditions.

Both IFNAR1^−/−^ and IFNLR1^−/−^ chickens exhibited impaired antibody production in response to KLH immunization. Phagocytosis and antigen processing are augmented in a properly functioning IFN system, thereby contributing to efficient antibody production in chickens (Wu and Kaiser, 2011). In avian species, macrophages and B cells are well-characterized antigen-presenting cells (APCs) that express MHCII molecules and present antigens to CD4^+^ T helper cells, leading to antibody production (Wu and Kaiser, 2011; Silva and Gallardo, 2020). On day 5 after booster immunization, the spleens of IFNAR1^−/−^ chickens exhibited fewer B cells, macrophages, and MHCII^+^ cells, including MHCII^+^ B cells and MHCII^+^ macrophages, than other genotypes. Loss of IFN sensing led to reduced Treg selection and reduced TCR repertoire diversity in a murine model (Ashby et al., 2024).

In ovo challenge of both IFNAR1^−/−^ and IFNLR1^−/−^ embryos revealed a complex interplay between chicken IFN signaling and viral pathogenesis, demonstrating that susceptibility to infection and subsequent immune responses highly depend on viral species and strain. Increased susceptibility and poor restrictions of viral replication in IFN receptor KO embryos were observed only for WSN33 (H1N1) strain. At the same time, no significant differences in viral titer were noted between the groups in response to H3N1. The case of low pathogenic avian H9N2 virus is particularly intriguing. Despite causing comparatively few deaths, H9N2 has garnered significant attention due to its potential genetic exchange with emerging zoonotic influenza viruses such as H7N9 and H10N8 subtypes (Chen et al., 2014; Pu et al., 2015). Interestingly, we observed that reduced H9N2 titers in IFNLR1^−/−^ did not correlate with improved embryo survival compared to other genotypes. This finding highlights the critical role of chicken IFN-λ as anti-inflammatory cytokines in maintaining immune balance during the antiviral defense against H9N2.

Because viral titers and type I IFN concentrations were measured in the allantoic fluid, while Mx expression reflected responses in the CAM, these analyses delineate distinct layers of IFN regulation. The CAM’s mixed cellular composition allows systemic-type I IFN signaling through vascular and mesodermal layers, whereas type III IFN activity via IFNLR1 is concentrated at the epithelial surface (Reuter et al., 2014; Nowak-Sliwinska et al., 2014). In our assays, Mx expression was measured at 24 hr post-infection, a time when type I IFN response may be highly active and capable of compensating for loss of type III IFN even in response to viruses with a predominant or prolonged epithelial phase like H9N2 and IBV (Raj and Jones, 1996; Yang et al., 2023). This redundancy may explain why type I IFN signaling alone was sufficient to drive robust Mx induction in IFNLR1 KO embryos for these viruses, while type III IFN remains essential for maximal Mx induction in infections that spread to both compartments (e.g. WSN33, H3N1) (de Wit et al., 2020; Swayne and Pantin-Jackwood, 2008). Thus, the observed differential impacts of receptor KO reflect both spatial compartmentalization and the timing of IFN responses, rather than a lack of type III IFN function in epithelial antiviral defense.

Although H3N1 is classified as a low pathogenic avian influenza A virus due to its monobasic hemagglutinin cleavage site, it has shown high virulence in adult layers, causing severe clinical infections and mortality (de Wit et al., 2020). Researchers revealed that H3N1 can rapidly replicate and disseminate systemically in chickens due to a neuraminidase mutation that activates plasminogen-mediated hemagglutinin (Schön et al., 2021). This mechanism mimics highly pathogenic strains and raises concerns about potential zoonotic transmission, particularly given the history of H3 subtypes in human infections. The in vivo H3N1 infection in WT, IFNLR1^−/−^, and IFNAR1^−/−^ hens revealed significant differences in disease progression and immune responses, emphasizing the critical role of type I IFN signaling in the defense mechanism against this H3N1 strain. In contrast, in mammalian systems, disruption of both type I and type III IFN pathways is generally required to produce comparable effects (Mordstein et al., 2008; Mordstein et al., 2010). These findings suggest that type I IFNs may play a more dominant and less redundant role in the response to the H3N1 strain and point to species-specific differences in IFN biology that warrant cautious comparison with mammalian systems.

Previous studies suggested that H3N1 replication in the oviduct is a critical factor in the onset of clinical disease and increased viral excretion (de Wit et al., 2020). Our current study provides new insights into H3N1 pathogenesis. The IFNAR1^−/−^ group displayed severe clinical symptoms and higher viral titers despite having intact oviducts and egg production. This observation suggests that H3N1 can establish infection and replicate independently of oviduct involvement, indicating a more complex pathogenic mechanism than previously thought. IFN-λ, initially described as an anti-inflammatory counterpart to type I IFN, is highly expressed by epithelial cells, which line the avian oviduct (Mork et al., 2014). Recent studies have revealed that mammalian IFN-λs can exert complex effects on innate and adaptive immunity, sometimes promoting inflammation in specific contexts (Broggi et al., 2020; Major et al., 2020; Goel et al., 2021), which agrees with the lower and delayed severity of oviduct infection and tissue damage in IFNLR1^−/−^ compared to the WT hens. This observation suggests a potential pro-inflammatory role for IFN-λ in the context of H3N1 infection in the avian oviduct. Similarly, mammalian type III IFNs can disrupt the lung epithelial barrier upon viral recognition, showing detrimental effects during chronic viral infection (Broggi et al., 2020). Moreover, IFN signaling, particularly IFN-λ, impairs lung repair by inducing p53 and inhibiting epithelial proliferation and differentiation in a mouse model of influenza infection (Major et al., 2020).

SOCS1 and SHP2 are pivotal regulators of IFN signaling, playing a crucial role in maintaining a delicate balance between effective immune responses and homeostasis (Röll et al., 2017; Qu, 2000). The lack of negative feedback mediated by SOCS1 and SHP2 in the IFNAR1^−/−^ hens, due to the absence of an antiviral state and increased viral spread, led to a continuous and significant secretion of IFN-α/β. These results agree with several studies that reported IFNAR1^−/−^ mice produce high levels of circulating IFNs. This phenomenon has been attributed to the lack of an IFNAR1 receptor to bind and internalize type I IFNs, reduced clearance of IFNs from circulation, and continued production of IFNs by cells in response to ongoing stimuli, resulting in their accumulation (McNab et al., 2015; Sheehan et al., 2015).

Type I IFNs are crucial in moderating leukocyte infiltration and the inflammatory cascade. However, disrupting the IFNAR1 receptor can lead to a dysregulated cytokine response and excessive inflammation (Seo et al., 2011; Sharma et al., 2022). In IFNAR1^−/−^ hens, eliminating type I IFN signaling and unaffected expression of inflammatory cytokines allows the H3N1 virus to spread further. The increased presence of viral components in the system continuously amplifies inflammatory cytokine production, potentially leading to a cytokine storm. Our observations support this hypothesis, as we found increased mRNA expression of pro-inflammatory cytokines in the spleen of IFNAR1^−/−^ hens, which indicates a clear activation of Th1 and Th22 pathways in these birds (Kaiser, 2010; Bean and Lowenthal, 2022; Eyerich et al., 2009). The simultaneous activation of multiple T helper cell subsets likely contributed to the rapid onset of severe symptoms observed in IFNAR1^−/−^ hens. Over-induction of Th1 and Th22 responses can lead to significant tissue damage and chronic inflammation, potentially increasing the risk of autoimmune-like reactions when left unregulated. Tregs normally modulate these responses by producing anti-inflammatory cytokines such as IL-10 and TGF-β (Burkhardt et al., 2022). However, our findings of unchanged expression of genes associated with the Tregs (FOXP3, IL-10, TGF-β) suggest that Treg activation was insufficient in the IFNAR1^−/−^ group.

Previous research on murine models has demonstrated that reduced inflammation can confer resistance to influenza infection, even in elevated viral loads (Herold et al., 2008; Lin et al., 2008). These observations suggest that the inflammatory response triggered by the virus may play a more crucial role in influenza pathology than the viral burden itself. Based on this understanding, we propose that the heightened inflammatory response observed in IFNAR1^−/−^ hens is a key factor in the increased pathology associated with H3N1 infection. Our findings indicate that the IFNAR1 receptor is critical for preserving the delicate balance between potent antiviral immunity and the potentially harmful consequences of unrestrained inflammatory responses.

The present study uncovered the critical roles of type I and III IFNs on antibody production and baseline immune cell populations in chickens. Furthermore, these KO models revealed that type I and type III IFNs play distinct and crucial roles in modulating viral pathogenesis, immune responses, and tissue-specific effects during avian influenza infection. The roles of these IFNs are virus strain-specific, with type I IFN showing particular importance in early defense against H3N1 avian influenza. By dissecting the complexities of avian IFN responses, these chicken models are critical for developing more effective strategies to control avian viral infections, including avian influenza, impacting veterinary medicine and zoonotic disease control.

## Materials and methods

The Key resources table is provided in Appendix 1.

### Animals

Specific pathogen-free (SPF) hatching eggs from white Lohman selected leghorn (LSL) were obtained from ValoBioMedia GmbH (Osterholz-Scharmbeck, Germany). These chickens were housed at the TUM Animal Research Center (TUM School of Life Sciences, Weihenstephan). They were provided with water and commercial feed ad libitum. All animal experiments were conducted following current laws and approved by the government of Upper Bavaria (experiment licenses: ROB-55.2-2532.Vet_02-20-13 and ROB-55.2-2532.Vet_02-23-100).

### Generation of IFNAR1 and IFNLR1 KO PGCs

LSL PGCs were derived from the blood of embryonic vasculature at stages 13–15, as described earlier (Hamburger and Hamilton, 1951). These PGCs, derived from male embryos, were cultured at 37°C in a 5% CO_2_ environment using modified KO-DMEM (Whyte et al., 2015). Guide RNAs targeting the coding region within exon 1 of the IFNAR1 chain of the IFN-α/β receptor and the epithelium-specific chain (IFNLR1) of the IFN-λ receptor (Supplementary file 1a) were designed and cloned separately into the PX458-Cas 9 vector (Addgene, USA), which contains eGFP as a selectable marker. An ssODN (Supplementary file 1a) was synthesized by Integrated DNA Technologies (IDT, Zellik, Belgium) to serve as a repair template for the IFNAR1 KO. The PGCs were transfected with the targeting vector (PX458-sgRNA) and sorted based on eGFP fluorescence with fluorescence-activated cell sorting (FACS) as previously described (Hellmich et al., 2020). Selected eGFP-positive PGCs were plated on a 48-well plate to expand single-cell clones. Sanger sequencing was employed to identify the desired genetic modification.

### Generation of IFNAR1^−/−^ and IFNLR1^−/−^ chickens

After successful clonal selection, PGCs were genotyped to identify clones with a KO. These clones were used to generate chimeric roosters. For IFNAR1, the INDEL was pre-designed using CRISPR-mediated HDR technology, resulting in a 7 bp deletion that produced a stop codon after 21 bp. For IFNLR1, the clone with the maximum deletion (28 bp) was selected as it allowed for the easy design of an IFNLR1 KO-specific PCR genotyping assay and produced a stop codon after 87 bp (Figure 1a and b). The KO PGC clones were injected into the vasculature of 65-hr-old embryos and incubated until the hatch. Upon sexual maturity, sperm was collected, and germline transmission was evaluated as previously described (Schusser et al., 2013; van de Lavoir et al., 2006). Germline-positive chimeric roosters were bred with WT hens to obtain heterozygous animals. IFNAR1 and IFNLR1 homozygous birds were generated by crossing the heterozygous birds (IFNAR1^+/−^) and (IFNLR1^+/−^), respectively. Following the generation of different transgenic lines, the birds were monitored until sexual maturity for any harmful phenotypes, such as impacts on weight gain and reproductive capabilities. Additionally, homozygous roosters and chickens were bred within each line to test for growth rate and the ability to produce fertile sperm or eggs.

### Genotyping of genetically modified chickens

For the genotyping of IFNAR1 KO, TaqMan assay was designed and performed using 5x HOT Firepol Probe qPCR Mix Plus (Solis Biodyne, Estonia). Detailed information about the sequence of the 1263-IFNAR1-For and 1264-IFNAR1-Rev primers (IDT, Zellik, Belgium), as well as 1291-IFNAR1 MUT-probe_HEX and 1262-IFNAR1 WT-probe_FAM probes (Eurofins, Ebersberg, Germany), can be found in Supplementary file 1b. The following cycler conditions were used: pre-read stage at 60°C for 30 s, hold stage at 95°C for 15 min, followed by 40 cycles of 95°C for 15 s, and 60°C for 1 min, and a post-read stage of 60°C for 30 s. The genotyping of IFNLR1 KO was done via endpoint PCR using Firepol DNA Polymerase (Solis Biodyne, Estonia) employing the IFNLR1 primers shown in Supplementary file 1b. The following PCR conditions were used: initial denaturation at 95°C for 3 min, followed by 40 cycles of 95°C for 15 s, 59°C for 30 s, 72°C for 15 s, and a final extension step of 72°C for 5 min. The WT sample produced an amplicon size of 250 bp, the homozygote produced an amplicon size of 222 bp, and the heterozygote sample produced amplicon sizes of both 250 bp and 222 bp.

### Western blot and RT-PCR to examine successful KO of IFNAR1/IFNLR1

To confirm the deletion of IFNAR1, a western blot experiment was conducted to study the expression of Mx protein in chicken CEFs treated with recombinant IFN-α. CEFs were isolated following a previously established protocol from 10-day-old embryos, with genotype confirmation through blood collection from a 0.5 cm² window in the eggshell that allowed access to the embryonic vasculature (Hernandez and Brown, 2010). The CEFs were cultured and maintained as described earlier (Hellmich et al., 2020). WT, IFNAR1^+/−^, and IFNAR1^−/−^ CEFs (n=3) were cultured in a six-well plate and stimulated with recombinant IFN-α (500 U/ml) for 12 hr. A control group of CEFs was treated with CEF medium without recombinant IFN-α. Following the stimulation period, both control and treated CEF cells were trypsinized and collected for SDS-PAGE and western blot analysis to study the expression of chicken Mx protein (75 kDa) and the control protein chicken β-actin (42 kDa). Mouse anti-human monoclonal IgGκ antibodies targeting MxA (M143; Merck, Germany) and mouse anti-chicken monoclonal IgG2b antibodies targeting β-actin (Thermo Fisher Scientific, Germany) were used as primary antibodies as described earlier (Schusser et al., 2011). Horseradish peroxidase (HRP)-conjugated donkey anti-mouse IgG was used as the secondary antibody (Thermo Fisher Scientific, Germany). Blots were developed with homemade ECL substrate, and the signal was detected using the Vilber Lourmat Fusion Fx system (Eberhardzell, Germany). Additionally, an experiment examined the absence of viral resistance in IFNAR1^−/−^ embryos compared to WT embryos after stimulation with recombinant IFN-α. 10-day-old chicken embryos were stimulated with 1.5×10^5^ U of recombinant IFN-α in 100 µl PBS 12 hr before and at the time of infection, while control groups did not receive IFN-α stimulation. All groups of embryos (four embryos per group) were infected with 1000 FFU of WSN33, and the viral load in the allantoic fluid was analyzed 24 hr post-infection by titration on the Madine-Darby canine kidney (MDCK) cells, as described earlier (Schusser et al., 2011).

To confirm the deletion of IL-28Rα (IFNLR1), various tissues (spleen, liver, lung, intestine, bursa, heart, and cecum) were collected from 18-day embryos of WT, IFNLR1^+/−^, and IFNLR1^−/−^. To ensure that the expression of IL-28Rα is induced, these embryos were challenged with 1000 FFU of H3N1 strain 12 hr before organ collection. The organ samples were rinsed with PBS and homogenized using a SpeedMill Homogenizer (Analytik Jena). After genotyping the embryos, RNA was isolated using the TRIzol method (VWR, Germany) (Panda et al., 2020), followed by complementary DNA (cDNA) synthesis with the GoScript Reverse Transcription Mix (Promega, USA). PCR was conducted using β-actin primers (277-FW and 278-RV) and IL-28Rα primers (1642-FW and 1643-RV) (Supplementary file 1c). The PCR was performed with Firepol DNA Polymerase (Solis Biodyne, Estonia) according to the manufacturer’s instructions. The PCR conditions were as follows: initial denaturation at 95°C for 3 min, followed by 40 cycles for β-actin or 35 cycles for IL-28Rα of 95°C for 30 s, 56°C for β-actin or 62°C for IL-28Rα for 30 s, and 72°C for 25 s. A final extension step was carried out at 72°C for 5 min. Nuclease-free water was used as a negative control for both. A 1.5% Tris-borate-EDTA (TBE) gel was used to visualize the β-actin amplicon at 300 bp, and a 2% TBE gel was used to visualize the 108 bp amplicon for IL-28Rα.

### Experimental design

Various in vitro, in ovo, and in vivo experiments were conducted to characterize the IFNAR1^−/−^ and IFNLR1^−/−^ genetically modified lines. Sample sizes were determined based on standard practice in the field and prior experience. The first experiment aimed to study the impact of type I and III IFNs on growth development, immune response, and cecal microbiota. Heterozygous birds of each line were bred separately, resulting in WT, heterozygous, and homozygous birds for each line. At least 12 animals per group of WT, IFNAR1^−/−^, and IFNLR1^−/−^ were included in the study. Six chicks per group were euthanized at the age of 1 month to examine differences in immune cell populations in blood and spleen. Cecal contents from IFNLR1^−/−^ chickens and their WT siblings were collected for microbiota analysis. Animals were grouped by genotype, and individuals within each genotype were randomly selected and used consistently across analyses. In the second experiment, the ability of IFNAR1^−/−^ and IFNLR1^−/−^ chickens to respond to immunization and secrete antibodies was investigated. KLH was used as a model T cell-dependent antigen to assess adaptive immune and antibody responses, providing a robust system for evaluating B cell activation and humoral immunity in KO models (Schusser et al., 2016). Five-week-old WT, IFNLR1^−/−^, and IFNAR1^−/−^ chicks were immunized via intramuscular injection with 300 µg KLH, mixed 1:1 with incomplete Freund’s adjuvant. A booster injection of 300 µg KLH, mixed 1:1 with Freund’s incomplete adjuvant, was administered 2 weeks after the initial immunization. Plasma samples were collected from all groups before immunization (day 0), on day 5 post-primary immunization (PP), and on days 3 and 5 post-booster immunization (PB). The total concentration of IgM, IgY, and levels of KLH antigen-specific IgM and IgY were assessed using enzyme-linked immunosorbent assay (ELISA). Peripheral blood mononuclear cells (PBMCs) were isolated on day 3 post-PP, and splenocytes were isolated on day 5 PB for FACS analysis of immune and MHC-positive cells. Spleen samples were used to study TCR repertoire. At least six animals per group were included (Figure 4—figure supplement 1).

In the third experiment, an in ovo study was conducted to examine the effects of IFNAR1 and IFNLR1 KO on viral replication, survival probability, Mx, and IFNλ mRNA expression, as well as IFN-α/β concentrations in WT, IFNLR1^−/−^, and IFNAR1^−/−^ embryos (Figure 6—figure supplement 1). Fertilized eggs from a crossing of heterozygous roosters and hens of each line were collected and incubated until embryonic day 11. The eggs were infected with 1000 FFU of different influenza strains (WSN33(H1N1)/H3N1/H9N2) and IBV-Beaudette strain separately, as described previously (Brauer and Chen, 2015). The experiment was performed in a blinded manner, with investigators unaware of the genotype during infection and sample processing; embryos were genotyped after sample collection and subsequently assigned to groups (WT and homozygous) for analysis. Details about the viral strains used in this study are provided in Supplementary file 1d. Twenty-four hours post-infection, the allantois fluid and breast tissue were collected for viral titration and genotyping, respectively. The CAM was also sampled 24 hr post-infection for gene expression studies. As previously described, the viral titers of WSN33, H3N1, and H9N2 were quantified by titration on MDCK cells (Schusser et al., 2011). For IBV, qPCR was used to measure the viral expression in the CAM (Supplementary file 1c). The relative mRNA expression of Mx and IFNλ was measured in the CAM. IFN-α/β concentration was quantified by titration of allantoic fluids on CEC-32 #511 reporter cells expressing firefly luciferase under the control of the chicken Mx promoter (Schultz et al., 1995). Plasmid #511 contains resistance to the antibiotic Geneticin (G418, 250 μg/ml) to keep the cells under selection pressure. Both MDCK and CEC-32 #511 reporter cells were maintained under standard laboratory conditions and routinely tested for mycoplasma contamination.

In the fourth experiment, the antiviral and immunomodulatory properties of type I and type III IFNs in the defense against H3N1 were characterized under in vivo conditions (Figure 7—figure supplement 1). The experimental groups consisted of WT, IFNLR1^−/−^, and IFNAR1^−/−^ hens, with 10 hens in each group. Seven hens per group were infected with H3N1, while the remaining three were negative controls. Hens were assigned to experimental groups based on genotype, and infected animals were selected randomly within each group. The hens, 27 weeks of age and actively laying eggs, received an infectious dose of 10^6^ FFU of H3N1 in 0.2 ml PBS per hen via nasal and tracheal routes on day 0. The H3N1 virus (A/Chicken/Belgium/460/2019) was provided by Dr. Joris Pieter De Gussem (Poulpharm BV). All IFNAR1^−/−^ hens (n=7) were euthanized by day 2 post-infection due to early and severe symptoms. In comparison, four WT hens were euthanized on day 5, two on day 6, and one on day 7 post-infection. Similarly, five IFNLR1^−/−^ hens were euthanized on day 5 and two on day 7 post-infection based on symptom severity. Cloacal and tracheal swabs for viral RNA loads by RT-qPCR (Sid et al., 2017) and blood samples for quantifying plasma IFN-α/β concentrations are taken on day 0 (all groups), day 2 (only IFNAR1^−/−^), day 3 (WT and IFNLR1^−/−^), and days 5–7 post-infection (WT and IFNLR1^−/−^) based on the appearance and severity of symptoms. Blood plasma IFN-α/β concentrations were quantified using luciferase assay as described in experiment 3. Spleen samples were collected from all the infected animals for gene expression study. The oviducts were also collected from all the hens to study the impact of type I and type III IFN on the pathogenesis of H3N1 (Figure 7—figure supplement 1).

### Isolation of peripheral blood and splenic mononuclear cells

PBMCs and mononuclear spleen cells were isolated using Histopaque-1077 density gradient centrifugation (Sigma-Aldrich, Darmstadt, Germany). Collected blood was diluted 1:1 with PBS and layered onto Histopaque-1077, followed by centrifugation at 400×*g* for 20 min at room temperature without brake. The mononuclear cell layer was collected, washed twice with PBS, and resuspended in FLUO-Buffer (1× PBS +1% BSA+0.01% NaN_3_). For spleen samples, tissues were aseptically dissociated through a 100 μm cell strainer into PBS to obtain single-cell suspensions. After centrifugation, cells were layered onto Histopaque-1077 and processed as described for PBMCs to obtain the mononuclear fraction.

### Flow cytometry

Flow cytometric analyses were performed on PBMCs and mononuclear spleen cells isolated as described above. The detailed panel of primary and secondary antibodies employed for the identification and characterization of B cells, monocytes, γδ T cells (TCR1), αβ T cells (TCR2+TCR3), and CD4-CD8 T cell subsets in experiment 1 is provided in Supplementary file 1e. Similarly, the antibody panel utilized for the analysis of B cells, monocytes, γδ T cells (TCR1), αβ T cells (TCR2+TCR3), major histocompatibility complex class I (MHCI), and class II (MHCII), MHCII+B cells, and MHCII+monocytes in experiment 2 are detailed in Supplementary file 1e. Briefly, 5×10^6^ cells per sample were washed with 1% BSA diluted in FLUO-Buffer (1× PBS+1% BSA+0.01% NaN3). To discriminate viable cells, the samples were incubated with Fixable Viability Dye eFluor 780 (eBioscience, Thermo Fisher Scientific, USA). Following a wash step with FLUO-Buffer, the cells were incubated with primary antibodies for 20 min, rewashed with FLUO-Buffer, and subsequently incubated with conjugated secondary antibodies for 20 min. After a final wash step, the cells were analyzed using the AttuneNXT flow cytometer (Thermo Fisher Scientific, USA) (Cossarizza et al., 2021). Data analysis was performed using FlowJo 10.8.1 software (FlowJo, Ashland, USA). Representative gating strategies employed for the first and second experiments are illustrated in Figure 3—figure supplement 1, Figure 4—figure supplement 3, respectively.

### Microbiome analysis

Cecum content was taken from 4-week-old WT and IFNLR1^−/−^ chickens. The caeca were isolated from the rest of the intestine, and one cecum was cut open longitudinally. The content was collected using sterile 10 µl inoculation loops and transferred to 2 ml lysing matrix B tubes (MP Biomedicals) filled with 1 ml DNA Stool Stabilizer (Invitek Diagnostics, Germany). The remaining digesta was cautiously removed, and the mucosa was scraped with the inoculation loop to transfer mucosa-associated bacteria. Samples were kept on ice and brought to the laboratory within 2 hr after collection. DNA was extracted and processed at the TUM Core Facility Microbiome. In brief, following mechanical cell lysis, DNA was purified on columns (Machery-Nagel, Düren, Germany). The V3/V4 region of the 16S ribosomal RNA (rRNA) gene was amplified by 25 cycles of a two-step PCR with primers 341FW and 785RV using a combinatorial dual-barcoding strategy. Amplicon libraries were sequenced on an Illumina MiSeq (Illumina, San Diego, USA) following the manufacturer’s instructions. A detailed description of DNA isolation, library preparation, and sequencing can be found elsewhere (Reitmeier et al., 2020). Raw sequence reads were processed using IMNGS2 (https://www.imngs2.org) (Lagkouvardos et al., 2016), a platform based on UPARSE (Edgar, 2013). Zero-radius operational taxonomic units were calculated, and a 0.25% abundance filter was applied. The IMNGS2-created output files were further processed using RHEA (Lagkouvardos et al., 2017), a modular pipeline for analyzing microbial profiles based on the 16S rRNA gene. Sequence reads were normalized using the normalization script in RHEA, and sequencing depth was sufficient, as depicted by the rarefaction curve (Figure 3—figure supplement 2).

### Enzyme-linked immunosorbent assay

ELISA was performed according to an earlier protocol with minor modifications (Schusser et al., 2016). For the quantification of total plasma IgM and IgY levels, 96-well maxisorp non-immuno ELISA plates (Thermo Fisher Scientific, Germany) were coated overnight at 4°C with 2 μg/ml goat anti-chicken IgM antibody (Bethyl Laboratories) or 2 μg/ml rabbit anti-chicken IgY antibody (Sigma-Aldrich, Darmstadt, Germany). Subsequently, the plates were blocked with PBS containing 4% skim milk and incubated with chicken plasma samples. Purified IgM (Rockland) at a concentration of 10 μg/ml and IgY (Jackson IR) at 2.9 μg/ml were used as standards. Bound IgM or IgY were detected using goat anti-chicken IgM-HRP conjugate (Bethyl Laboratories) or rabbit anti-chicken IgY-HRP conjugate (Sigma-Aldrich, Darmstadt, Germany), respectively, followed by development with 3,3',5,5'-tetramethylbenzidine substrate (Thermo Fisher Scientific, Germany). The OD was measured using a FluoStar Omega microplate reader (BMG LABTECH, Ortenberg, Germany) at a wavelength of 450 nm with a reference filter of 620 nm (version 5.91 5.70 R2). To determine antigen-specific IgM and IgY levels, the ELISA plates were coated with 2 μg/ml KLH (Sigma-Aldrich, Darmstadt, Germany) or BSA, and subsequent steps were performed as described for the detection of total plasma IgM and IgY, without the inclusion of purified immunoglobulin standards.

### TCR amplicon sequencing

TCR amplicons were prepared for next-generation sequencing as previously described (Früh et al., 2024). Briefly, total RNA was extracted from the spleen using the SV Total RNA Isolation System (Promega, Madison, USA), quantified on a Nanodrop ND-1000 (Thermo Fisher Scientific, Waltham, USA), and the quality was determined on a 2100 Bioanalyzer (Agilent Technologies, Santa Clara, USA). 900 ng of total RNA from samples with an RNA Integrity Number (RIN)≥8 were subjected to TCR constant (C) gene-specific 5’ RACE with molecular barcoding followed by two consecutive semi-nested and semi-step-out PCRs according to previously established protocols (Früh et al., 2024). The number of repeated cycles was 21 in the first PCR and 11 (β) or 13 (α) in the second PCR. Gel-purified PCR amplicons were sequenced on an Illumina MiSeq Instrument (Illumina, San Diego, USA) by Eurofins Genomics Germany GmbH (Ebersberg, Germany).

### TCR repertoire analysis

Raw read quality assessment with *FastQC v0.12.1* (Andrews, 2010), available online at: https://www.bioinformatics.babraham.ac.uk/projects/fastqc/, and *MultiQC v1.23* (Ewels et al., 2016) was followed by TCR analysis and annotation with *MiXCR v4.7.0* (Bolotin et al., 2015) as previously described (Früh et al., 2024). CDR3 clonotypes were annotated and quantified using a chicken-specific germline V(D)J gene segment library with *MiXCR* and analyzed with *R v4.4.1*. Samples were downsampled with the smallest sample size greater than 0.5 * 20th quantile as the target size. Analysis was performed using *tidyverse v2.0.0* packages. For the Kmer analysis, the first and last four amino acids of the CDR3 were excluded, and kmer counts were normalized to the total count of CDR3s in the sample.

### RNA isolation, cDNA synthesis, and qRT-PCR

Total RNA was isolated from the CAM and spleen using the TRIzol method (VWR, Germany) (Panda et al., 2020). The concentration of the extracted RNA was assessed by measuring the absorbance ratio at 260/280 nm using a BioSpec-nano spectrophotometer. An absorbance ratio between 1.9 and 2.0 was considered indicative of pure RNA. Bioanalyzer Systems 6000 nano kit was also used to measure RNA integrity, and only RIN values above 7 were considered for further processing. Subsequently, 400 ng of RNA was reverse-transcribed into cDNA using the GoScript Reverse Transcription Mix and Random Primers (Promega, USA), following the manufacturer’s protocol. The primer sequences used to analyze various gene expressions in the CAM and spleen are provided in Supplementary file 1f. A no-template control, containing PCR-grade water instead of the sample, was included to detect potential contamination. Real-time quantitative reverse transcription PCR (qRT-PCR) was performed using the QuantStudio5 system (QuantStudio Design & Analysis Software v1.5.2, Thermo Fisher Scientific) and the Promega GoTaq qRT-PCR kit (Promega, USA), following the manufacturer’s instructions. The annealing temperature for all the studied genes was 59°C. The expression of each gene was analyzed in triplicate and normalized to the 18S rRNA gene, which served as the housekeeping reference. For the third experiment, the mRNA abundance in unchallenged WT embryos was used to calculate the relative expression levels across all experimental groups. For the spleen analysis of H3N1 infection, the challenged WT group was used as a control. In addition, the genes that were downregulated in IFNAR1^−/−^ were presented as log2. The relative quantification of the studied genes was done using the 2^−ΔΔCt^ method.

### Scoring and histology of the oviduct

Lesions were scored in the reproductive tract in all in vivo H3N1-challenged groups and negative controls (Sid, 2025). Magnum grade 0: normal with physiological height of mucosa, Magnum grade 1: mild lymphoplasmacellular salpingitis with physiological height of mucosa, Magnum grade 2: moderate lymphoplasmacellular salpingitis with prominent atrophy of the mucosa, Magnum grade 3: severe lymphoplasmacellular salpingitis with severe atrophy of the mucosa. The oviduct (infundibulum, magnum) of day 5 post-infection of the unchallenged WT group, as well as WT and IFNLR1^−/−^ (challenged with H3N1), were used for histological assessment. The samples were fixed in 10% neutral-buffered formalin and processed routinely. Four-micrometer tissue sections were stained with hematoxylin and eosin for light microscopy. The sections’ analysis addressed histopathological changes and signs of inflammatory reactions, including hyperemia, edema, loss of epithelial cells, and infiltration of immune cells.

### Statistical analysis

Statistical analyses were performed using SPSS statistics software (version 28.0.1.1, IBM). Data were analyzed using one-way or two-way ANOVA followed by Tukey’s post hoc multiple-comparison test, as appropriate for the experimental design. All p-values≤0.05 were marked as significant. Graphs were designed with GraphPad Prism (version 9.3.1, Dotmatics). Statistics of the microbiome analysis were performed using the Wilcoxon rank-sum pairwise test and PERMANOVA. For the TCR repertoire, statistical analyses were conducted in *R* as previously described (Früh et al., 2024). A negative binomial generalized linear model (GLM) was fitted using the *glm.nb*() function from the *MASS* package v7.3-61 (Venables and Ripley, 2002) available at: https://www.stats.ox.ac.uk/pub/MASS4/ with TCR clonotype counts of V genes as the response variable and the experimental group (genotype and immunization) as predictors. Post hoc pairwise comparisons among the levels of predictors were conducted with the *emmeans* package v1.10.3 (Fox and Weisberg, 2018), available at: https://socialsciences.mcmaster.ca/jfox/Books/Companion/, applying Holm adjustment for multiple comparisons.

## Data Availability

Raw sequencing data generated in this study have been deposited in the NCBI Sequence Read Archive. The 16S rRNA amplicon sequencing data from the cecal microbiota analysis are available under BioProject accession PRJNA1472969. The T cell receptor alpha and beta repertoire sequencing data from chicken spleen samples are available under BioProject accession PRJNA1473170. Source data files containing the original uncropped and labelled gel and western blot images are provided for the relevant figures. Supplementary file 1 contains the sgRNA and ssODN sequences, primers and probes, virus strains, antibodies, and qPCR primer information used in this study. The following datasets were generated: AlhussienMN VikkulaHK KlingerR ZennerC FrühSP NegiR von HeylT SchleibingerS BrunnerM BerghofTVL AvolioL ReichA SchadeB SchusserBL RautenschleinS PreisingerR SidH 2026Cecal microbiome analysis of IFNLR1 knockout and wild-type chickensNCBI BioProjectPRJNA1472969 AlhussienMN VikkulaHK KlingerR ZennerC FrühSP NegiR von HeylT SchleibingerS BrunnerM BerghofTVL AvolioL ReichA SchadeB SchusserBL RautenschleinS PreisingerR SidH SchusserB 2026Gallus gallus breed:Lohman selected leghorn (chicken)NCBI BioProjectPRJNA1473170

## Additional files

Supplementary file 1. Key experimental resources used in this study, including gene-editing constructs, primers, probes, virus strains, and antibodies.

MDAR checklist

## Appendix 1

**Appendix 1—key resources table:**

Reagent type (species) or resource	Designation	Source or reference	Identifiers	Additional information
Gene (*Gallus gallus*)	IFNAR1	NCBI Gene	GeneID: 395665	Target gene encoding the type I IFN receptor chain (IFNAR1) studied in this work.
Gene (*Gallus gallus*)	IFNLR1; IL-28Rα	NCBI Gene	GeneID: 419694	Target gene encoding the epithelium-specific type III IFN receptor chain (IFNLR1) studied in this work.
Strain, strain background (*Gallus gallus*)	Lohman selected leghorn chickens	Lohman		SPF hatching eggs used to derive animals; adult in vivo H3N1 challenge experiments used hens; housed at TUM Animal Research Center.
Genetic reagent (*Gallus gallus*)	IFNAR1^−/−^ chicken line	This paper		Newly generated CRISPR/HDR IFNAR1 knockout line; 7 bp deletion causing a stop codon after 21 bp; maintained at Technical University of Munich; The line is available upon reasonable request from the Reproductive Biotechnology, TUM School of Life Sciences, Technical University of Munich
Genetic reagent (*Gallus gallus*)	IFNLR1^−/−^ chicken line	This paper		Newly generated CRISPR/Cas9 IFNLR1 knockout line; 28 bp deletion causing a stop codon after 87 bp; maintained at Technical University of Munich; The line is available upon reasonable request from the Reproductive Biotechnology, TUM School of Life Sciences, Technical University of Munich
Genetic reagent (*Gallus gallus*)	IFNAR1 knockout PGC clone	This paper		Primordial germ cell clone carrying the IFNAR1 knockout allele used to generate germline chimeras. PGC-derived material was generated from male PGCs.
Genetic reagent (*Gallus gallus*)	IFNLR1 knockout PGC clone	This paper		Primordial germ cell clone carrying the IFNLR1 knockout allele used to generate germline chimeras. PGC-derived material was generated from male PGCs.
Cell line (*Gallus gallus*)	Chicken embryonic fibroblast cells, CEFs	This paper		Primary cultures derived from ED10 embryos; genotyped as WT, IFNAR1^+/−^ or IFNAR1^−/−^ before IFN-α stimulation and western blot analysis.
Cell line (*Coturnix coturnix*)	CEC-32 #511 IFN reporter cells			Quail reporter cells expressing firefly luciferase under control of the chicken Mx promoter; maintained with G418 (250 µg/ml).
Cell line (*Canis lupus familiaris*)	MDCK cells			Madine-Darby canine kidney cells used for influenza virus titration; laboratory stock.
Transfected construct (*Gallus gallus*)	PX458-Cas9-2A-eGFP-IFNAR1-sgRNA	This paper	Plasmid: PX458	PX458-Cas9/eGFP backbone carrying IFNAR1-targeting sgRNA; sgRNA sequence provided in Supplementary file 1a.
Transfected construct (*Gallus gallus*)	PX458-Cas9-2A-eGFP-IFNLR1-sgRNA	This paper	Plasmid: PX458	PX458-Cas9/eGFP backbone carrying IFNLR1-targeting sgRNA; sgRNA sequence provided in Supplementary file 1a.
Strain, strain background (Influenza A virus)	WSN/33 (H1N1)	Supplementary file 1d		Virus strain used for in ovo challenge; titer 4500 U/µl; infection dose 1000 U/100 µl.
Strain, strain background (Influenza A virus)	A/Chicken/Belgium/460/2019 (H3N1)	Supplementary file 1d		Low pathogenic avian influenza strain used for in ovo and in vivo challenge; infection dose 1000 U/100 µl for in ovo experiments.
Strain, strain background (Influenza A virus)	A/chicken/Saudi Arabia/CP7/1998 (H9N2)	Supplementary file 1d		Low pathogenic avian influenza strain used for in ovo challenge; infection dose 1000 U/100 µl.
Strain, strain background (Infectious bronchitis virus)	IBV-Beaudette strain	Supplementary file 1d		Avian gamma coronavirus strain used for in ovo challenge; infection dose 1000 U/100 µl.
Antibody	Anti-human MxA (mouse monoclonal)	Merck (Cat# MABF938)	Clone: M143	Primary antibody for western blot detection of chicken Mx protein (75 kDa).
Antibody	Anti-chicken β-actin (mouse monoclonal)	Thermo Fisher Scientific (MA5-15739)	Clone: BA3R	Primary antibody for western blot loading control (42 kDa).
Antibody	HRP-conjugated anti-mouse IgG (donkey polyclonal)	Thermo Fisher Scientific (Cat# 715-035-151)		Secondary antibody for western blot detection.
Antibody	Anti-chicken TCRγδ-BIOT (mouse monoclonal)	Biozol (Cat# SBA-8230-08)	Clone: TCR-1	Flow cytometry (0.625 µg/ml); Supplementary file 1e.
Antibody	Anti-chicken TCRαβ/Vβ1-BIOT (mouse monoclonal)	Biozol (Cat# SBA-8240-08)	Clone: TCR-2	Flow cytometry (2.5 µg/ml); Supplementary file 1e
Antibody	Anti-chicken TCRαβ/Vβ2-BIOT (mouse monoclonal)	Biozol (Cat# SBA-8250-08)	Clone: TCR-3	Flow cytometry (2.5 µg/ml); Supplementary file 1e
Antibody	Anti-chicken Bu1-FITC (mouse monoclonal)	Biozol (Cat# SBA-8395-02)	Clone: AV20	Flow cytometry (2.5 µg/ml); Supplementary file 1e
Antibody	Anti-chicken KUL01_UNLAB (mouse monoclonal)	Biozol (Cat# SBA-8420-01)	Clone: KUL01	Flow cytometry (2.5 µg/ml); Supplementary file 1e
Antibody	Anti-chicken CD8α_Pacific Blue (mouse monoclonal)	Biozol (Cat# 8220-26)	Clone: CT-8	Flow cytometry (0.625 µg/ml); Supplementary file 1e
Antibody	Anti-chicken CD8β_UNLAB (mouse monoclonal)	Biozol (Cat# 8280-01)	Clone: EP42	Flow cytometry (0.625 µg/ml); Supplementary file 1e
Antibody	Anti-chicken CD4_FITC (mouse monoclonal)	Biozol (Cat# 8210-02)	Clone: CT-4	Flow cytometry (0.625 µg/ml); Supplementary file 1e
Antibody	Anti-mouse IgG2a_PE (rat monoclonal)	Biozol (Cat# 1155-09)	Clone: SB84a	Secondary antibody for flow cytometry (0.125 µg/ml); Supplementary file 1e
Peptide, recombinant protein	Streptavidin_APC	VWR (Cat# 20-4317-U500)		Flow cytometry detection reagent for biotinylated antibodies (0.2 µg/ml); Supplementary file 1e.
Antibody	Anti-mouse IgG (H+L)-APC (goat polyclonal)	Biozol (Cat# SBA-1031-11L)	Polyclonal	Secondary antibody for flow cytometry (0.625 µg/ml); Supplementary file 1e
Antibody	Anti-chicken Bu-1-AF647 (mouse monoclonal)	Biozol (Cat# SBA-8395-31)	Clone: AV20	Flow cytometry (1:500); Supplementary file 1e
Antibody	Anti-chicken MHC I (mouse monoclonal)	Southern Biotech (Cat# 8345-01)	Clone: F21-2	Flow cytometry (1:200); Supplementary file 1e
Antibody	Anti-chicken MHC II-AF488 (mouse monoclonal)	Biozol (Cat# 8350-30)	Clone: 2G11	Flow cytometry (1:1000); Supplementary file 1e
Sequence-based reagent	IFNAR1 ssODN part 1; IFNAR1 ssODN part 2	Integrated DNA Technologies; this paper		HDR repair templates for IFNAR1 knockout; sequences provided in Supplementary file 1a.
Sequence-based reagent	IFNLR1 sgRNA; IFNAR1 sgRNA	This paper		CRISPR/Cas9 sgRNAs; sequences and PAMs provided in Supplementary file 1a.
Sequence-based reagent	IFNAR1 and IFNLR1 genotyping primers and probes	This paper		Primer/probe sequences provided in Supplementary file 1b.
Sequence-based reagent	Sexing, RT-PCR, IBV qPCR and 28S qPCR primers/probes	This paper		Primer/probe sequences provided in Supplementary file 1c.
Sequence-based reagent	qPCR primers for cytokines, ISGs, and IFN signaling genes	This paper		Primer sequences, amplicon sizes, and accessions provided in Supplementary file 1f.
Sequence-based reagent	TCR amplicon sequencing primers	Früh et al., 2024		TCR constant gene-specific 5' RACE and semi-nested/step-out PCR primers; see Materials and methods.
Peptide, recombinant protein	Recombinant chicken IFN-α	Schultz et al., 1995		Used for stimulation of CEFs and ED10 embryos; 500 U/ml for CEF stimulation; 1.5×10^5^ U for embryo stimulation.
Peptide, recombinant protein	Keyhole limpet hemocyanin; KLH	Sigma-Aldrich		Model T cell-dependent antigen used for immunization; 300 µg per injection.
Commercial assay or kit	5x HOT Firepol Probe qPCR Mix Plus	Solis Biodyne		Used for TaqMan genotyping of IFNAR1 KO animals.
Commercial assay or kit	Firepol DNA Polymerase	Solis Biodyne		Used for endpoint PCR genotyping and RT-PCR assays.
Commercial assay or kit	GoScript Reverse Transcription Mix	Promega	Cat#A5001	Used for cDNA synthesis.
Commercial assay or kit	GoTaq qRT-PCR kit	Promega	Cat#A6002	Used for qRT-PCR gene expression analysis.
Commercial assay or kit	Histopaque-1077	Sigma-Aldrich	Cat#10771	Used for PBMC and splenic mononuclear cell isolation.
Commercial assay or kit	SV Total RNA Isolation System	Promega	Cat#Z3100	Used for RNA extraction before TCR amplicon sequencing.
Commercial assay or kit	Fixable Viability Dye eFluor 780	eBioscience; Thermo Fisher Scientific	Cat#65-0865-14	Flow cytometry viability dye; 1:1000; Supplementary file 1e.
Commercial assay or kit	Maxisorp non-immuno ELISA plates	Thermo Fisher Scientific		Used for IgM/IgY and KLH-specific ELISA assays.
Chemical compound, drug	TRIzol reagent	VWR		Used for RNA isolation from CAM, spleen, and tissues.
Chemical compound, drug	Incomplete Freund’s adjuvant	Sigma-Aldrich		Mixed 1:1 with KLH for primary and booster immunization.
Chemical compound, drug	Geneticin; G418	Thermo Fisher Scientific		Used at 250 µg/ml to maintain CEC-32 #511 reporter cells under selection pressure.
Chemical compound, drug	Homemade ECL substrate	This paper		Prepared in-house; luminol-based HRP chemiluminescent substrate used for western blot signal development.
Software, algorithm	GraphPad Prism	Dotmatics	Version:9.3.1; RRID:SCR_002798	Used for graph generation.
Software, algorithm	SPSS Statistics	IBM	Version:28.0.1.1; RRID:SCR_002865	Used for statistical analyses.
Software, algorithm	FlowJo	FlowJo; Ashland	Version:10.8.1; RRID:SCR_008520	Used for flow cytometry data analysis.
Software, algorithm	QuantStudio Design & Analysis Software	Thermo Fisher Scientific	Version:1.5.2	Used for qRT-PCR data acquisition/analysis.
Software, algorithm	FastQC	Babraham Bioinformatics	Version:0.12.1; RRID:SCR_014583	Used for raw read quality assessment.
Software, algorithm	MultiQC	Ewels et al., 2016	Version:1.23	Used to summarize sequencing quality-control results.
Software, algorithm	MiXCR	Bolotin et al., 2015	Version:4.7.0; RRID:SCR_018725	Used for TCR analysis and annotation.
Software, algorithm	R	R Foundation for Statistical Computing	Version:4.4.1; RRID:SCR_001905	Used for TCR repertoire and statistical analyses.
Software, algorithm	tidyverse	R package	Version:2.0.0	Used for data plotting in R.
Software, algorithm	MASS	R package	Version:7.3–61	Used for negative binomial generalized linear models.
Software, algorithm	emmeans	R package	Version:1.10.3	Used for post hoc pairwise comparisons.
Software, algorithm	IMNGS2	Lagkouvardos et al., 2016		Used for processing 16S rRNA gene amplicon sequencing data.
Software, algorithm	RHEA	Lagkouvardos et al., 2017		Used for microbial profiling based on 16S rRNA gene amplicons.
Other	Illumina MiSeq	Illumina		Sequencing instrument used for 16S rRNA gene amplicon sequencing and TCR amplicon sequencing.
Other	AttuneNXT flow cytometer	Thermo Fisher Scientific		Instrument used for flow cytometry.
Other	Vilber Lourmat Fusion Fx system	Vilber Lourmat		Imaging system used for western blot signal detection.
Other	SpeedMill Homogenizer	Analytik Jena		Used for tissue homogenization.
Other	BioSpec-nano spectrophotometer	Shimadzu		Used for RNA concentration/quality assessment.
Other	2100 Bioanalyzer	Agilent Technologies		Used for RNA integrity assessment.
Other	FluoStar Omega microplate reader	BMG LABTECH		Instrument used for ELISA absorbance measurements at 450 nm with 620 nm reference filter; software version 5.91/5.70R2.
Other	QuantStudio 5 system	Thermo Fisher Scientific		Instrument used for qRT-PCR data acquisition.
Other	NanoDrop ND-1000	Thermo Fisher Scientific		Instrument used for RNA quantification before TCR amplicon sequencing.
