# Supplementary material for: Deciphering interferon functions in avian influenza using receptor knockout models in the natural host
Source: eLife. 2026 Jun 26;14:RP107855. doi: 10.7554/eLife.107855 (PMC13309126; doi:10.7554/eLife.107855)
Supplement: Figure 1—source data 1. [file elife-107855-fig1-data1.zip › Figure_1_source_data_1/Figure_1d_labelled_uncropped_gel.pdf]

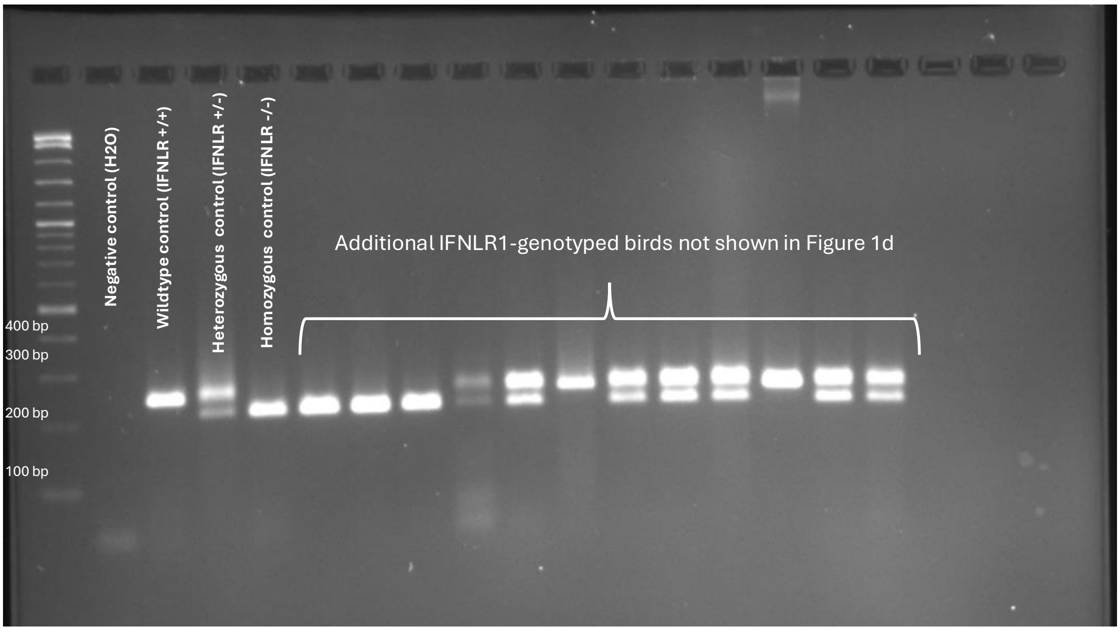

Figure 1-source data 1. Labelled uncropped gel image for the IFNLR1 PCR genotyping assay shown in Figure 1d. The gel shows the negative control (H2O), wildtype control ($IFNLR1^{+/+}$), heterozygous control ($IFNLR1^{+/-}$), and homozygous knockout control ($IFNLR1^{-/-}$). The expected bands are approximately 250 bp for the wildtype allele and 222 bp for the IFNLR1 knockout allele. Additional IFNLR1-genotyped birds not displayed in the main figure are also included in the uncropped gel.
