# Supplementary material for: Deciphering interferon functions in avian influenza using receptor knockout models in the natural host
Source: eLife. 2026 Jun 26;14:RP107855. doi: 10.7554/eLife.107855 (PMC13309126; doi:10.7554/eLife.107855)
Supplement: Figure 2—source data 1. [file elife-107855-fig2-data1.zip › Figure_2_source_data_1/Figure 2d_ IL-28R╬▒ RT-PCR gel and ╬▓-actin RT-PCR gel.pdf]

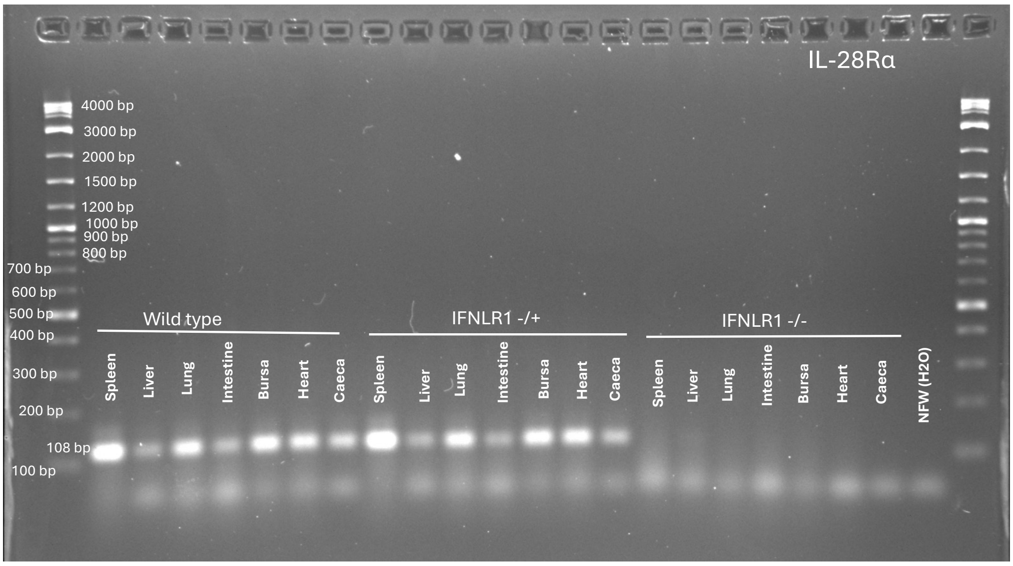

Figure 2-source data 1, Figure 2d, **IL-28Rα**. Labelled uncropped RT-PCR gel showing IL-28Rα amplification in tissues from WT, IFNLR1+/−, and IFNLR1−/− embryos.

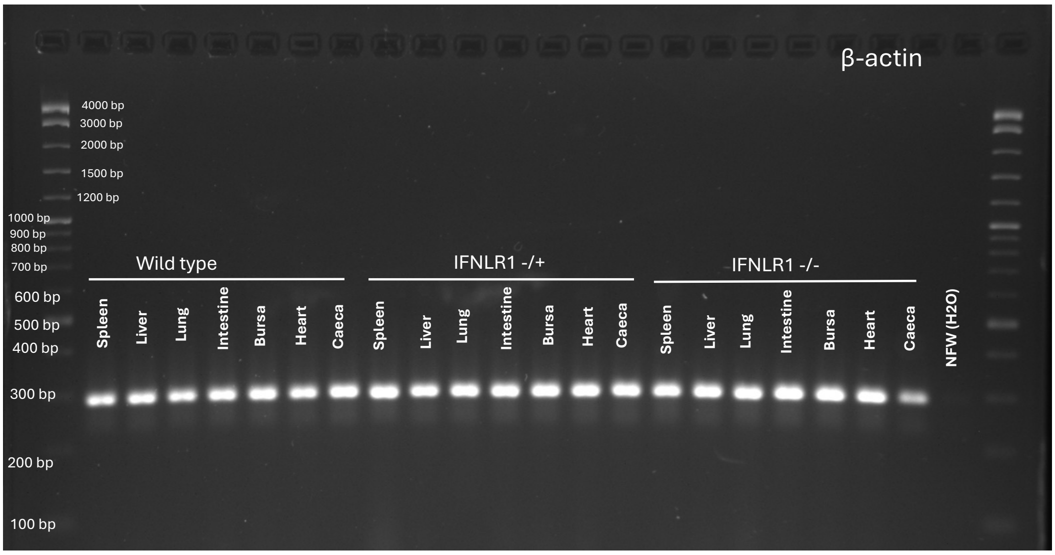

Figure 2-source data 1, Figure 2d, **β-actin**. Labelled uncropped RT-PCR gel showing β-actin amplification as internal control for the IL-28Rα RT-PCR analysis in Figure 2d.
