# Supplementary material for: Deciphering interferon functions in avian influenza using receptor knockout models in the natural host
Source: eLife. 2026 Jun 26;14:RP107855. doi: 10.7554/eLife.107855 (PMC13309126; doi:10.7554/eLife.107855)
Supplement: Figure 2—source data 1. [file elife-107855-fig2-data1.zip › Figure_2_source_data_1/Figure 2b_Mx western blot and ╬▓-actin western blot.pdf]

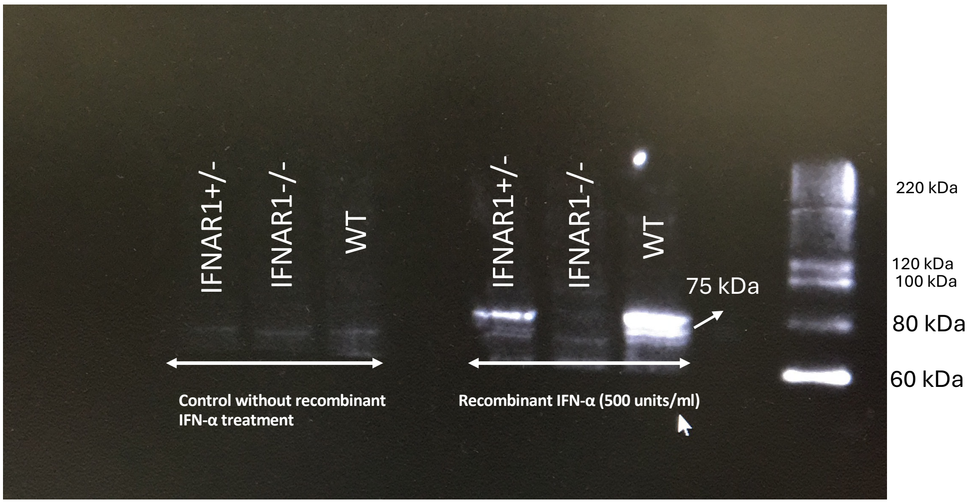

Figure 2-source data 1, Figure 2b, Mx. Labelled uncropped western blot showing Mx protein expression in WT, IFNAR1$^{+/-}$, and IFNAR1$^{-/-}$ chicken embryonic fibroblast cells with or without recombinant IFN-α treatment.

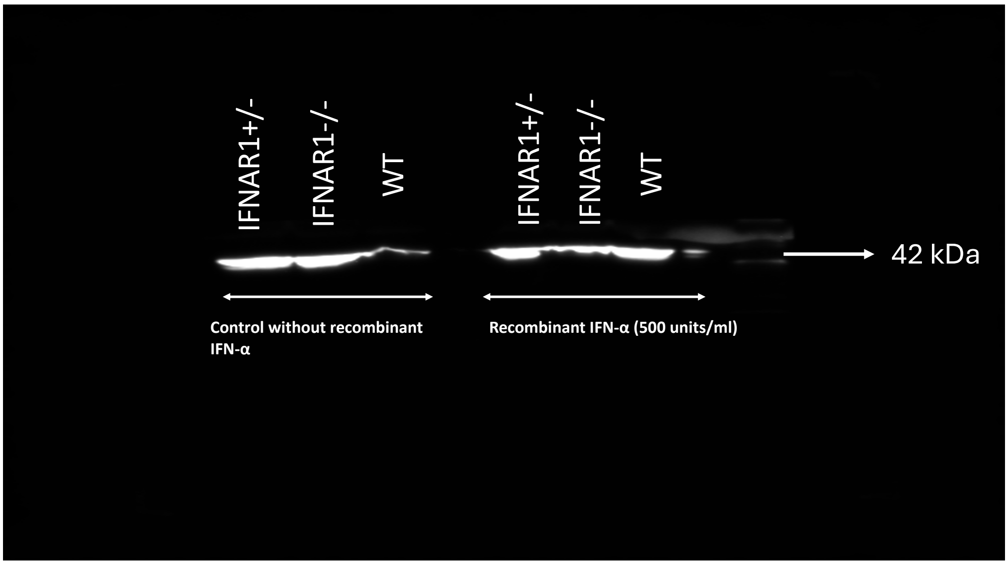

Figure 2-source data 1, Figure 2b, β-actin. Labelled uncropped western blot showing β-actin as loading control for the Mx western blot analysis in Figure 2b.
