# Supplementary material for: Deciphering interferon functions in avian influenza using receptor knockout models in the natural host
Source: eLife. 2026 Jun 26;14:RP107855. doi: 10.7554/eLife.107855 (PMC13309126; doi:10.7554/eLife.107855)
Supplement: Supplementary file 1. [file elife-107855-supp1.docx]

**Supplementary File 1**

**Deciphering interferon functions in avian influenza using receptor knockout models in the natural host**

**Mohanned Naif Alhussien et al.**

**Supplementary file 1a.** List of designed sgRNA and ssODN

| Gene name | Type | Purpose | Sequence (5´ - 3´) |
| --- | --- | --- | --- |
| IFNLR1 sgRNA | single guide RNA | CRISPR/Cas9 targeting | GGCAGCCCAGAGGTACGTCA  (PAM: TGG) |
| IFNAR1 sgRNA | single guide RNA | CRISPR/Cas9 targeting | GTGTGCCTCTGGGCGGCTAG  (PAM: CGG) |
| IFNAR1 ssODN part 1 | repair construct | HDR | GCAGTCGTCAGAGGCTTCCGGTA  AGGGTGAGCGCAGCCACGGACTG  ATGGCTGAGGCGGCGTGTGCCTCT |
| IFNAR1 ssODN part 2 | repair construct | HDR | TAGCGGCTGTGCTGCTTTGTGTCCT  GGTCGTGGTGTCCCGGTGCTGTGCA  GGTGAGCCGAGCACGGTGCA |

**Supplementary file 1b.** Primers and probes were used for the genotype of IFNAR1 and IFNLR1 KO chicken.

| Genes | Internal name & Sequence (5′→3′) of Primers/Probes | Tm °C |
| --- | --- | --- |
| IFNLR1  Primers | FW (1316): 5' GTTCTGCTTCAGCATGTCTGCAT 3'  RV (1317): 5' TCCAACCTCAGCCATACCACG 3' | 59 |
| IFNAR1  Primers | FW (1263): 5’- CGCAGCCACGGACTGAT -3’  RV (1264): 5’- ACGACCAGGACACAAAGCA-3’ | 60 |
| IFNAR1  Probes | IFNAR1 MUT-probe_HEX (1291): [HEX]CAGCCGCTAAGAGGCA[MGBEQ]  IFNAR1 WT-probe_FAM (1262): [FAM]CCGCTAGCCGCCCAG[MGBEQ] | 60 |

**Supplementary file 1c.** Primers used for sexing and various RT-PCR assays and IBV qPCR.

| Purpose | Internal name & Sequence of Primers | Tm °C |
| --- | --- | --- |
| Sexing | Z chromosome FW (1): 5’- AAGCATAGAACAATGTGGGAC-3’  Z chromosome RV (2): 5’- AACTCTGTCTGGAAGGACTT-3’  W chromosome FW (3): 5’- CTATGCCTACCACMTTCTATTTGC3’  W chromosome RV (4): 5’- AGCTGGAYTTCAGWCATCTTCT-3’ | 56 |
| β-actin  RTPCR | FW (277): 5‘- TACCACAATGTACCCTGGC-3‘  RV (278): 5‘- CTCGTCTTGTTTTATGCGC-3‘ | 56 |
| IL-28Rα RTPCR | FW (1642):5’AGTGCTGGCAACTCTGTGCT3‘  RV (1643): 5’TCCTTCTCCTGGAGTCCATGTCA3’ | 62 |
| IBV qPCR | FW (1141): 5- GCTTTT GAGCCTAGC GTT-3‘  RV (1142): 5‘-GCCATGTTG TCACTG TCTATTG-3‘  IBV probe (1140): 5‘-FAM-CACCACCAGAACCTGTCACCTC-BHQ1-3‘ | 59 |
| 28S qPCR | FW (1277): 5- GGCGAAGCCAGAGGAAACT -3‘  RV (1278): 5‘-GACGACCGATTTGCACGTC -3‘  28S probe (1279): 5‘-HEX-AGGACCGCTACGGACCTCCACCA-BHQ1-3‘ | 59 |

**Supplementary file 1d.** Virus strains used for the *in ovo* challenge.

| Virus strain | Full name | Titer (U/µl) | Infection dose (U/100µl) |
| --- | --- | --- | --- |
| WSN | WSN/33 (H1N1) | 4500 | 1000 |
| H3N1 | A/Chicken/Belgium/460/2019 | 21000 | 1000 |
| H9N2 | A/chicken/Saudi Arabia/CP7/1998 | 24000 | 1000 |
| IBV | Infectious bronchitis Virus Beaudette strain | 1400 | 1000 |

**Supplementary file 1e.** clones and concentrations of antibodies used for the FACS studies of experiment 1 and experiment 2.

| Primary Antibody | Internal code | Company | Clone | Concentration |
| --- | --- | --- | --- | --- |
| Experiment 1: FACS analysis of B cells, monocytes, γδ T cells (TCR1), αβ T cells (TCR2 + TCR3) and CD4-CD8 subsets of T cells | | | | |
| mouse IgG1 Anti-chicken  TCRγδ-BIOT | 42 | Biozol | TCR-1 | 0.625 μg/mL |
| mouse IgG1 anti-chicken  TCRαβ/Vb1-BIOT | 43 | Biozol | TCR-2 | 2.5μg/mL |
| mouse IgG1 anti-chicken  TCRαβ/Vb2-BIOT | 44 | Biozol | TCR-3 | 2.5μg/mL |
| mouse IgG1 anti-chicken Bu1-  FITC | 25 | Biozol | AV20 | 2.5μg/mL |
| mouse IgG1 anti-chicken  KUL01_UNLAB | 23 | Biozol | KUL01 | 2.5μg/mL |
| mouse-IgG1_anti-chicken  CD8α_PacBlue | 60 | Biozol | CT-8 | 0.625 μg/mL |
| mouse IgG2a anti-chicken  CD8β_UNLAB | 61 | Biozol | EP42 | 0.625 μg/mL |
| mouse IgG1 anti-chicken  CD4_FITC | 59 | Biozol | CT-4 | 0.625 μg/mL |
| **Secondary Antibody** |  | **Company** | **Clone** | **Concentration** |
| rat anti-mouse IgG2a_PE | 62 | Biozol | SB84a | 0.125 μg/mL |
| Streptavidin_APC | 45 | VWR | - | 0.2 μg/mL |
| goat anti-mouse IgG (H+L)-  APC | 26 | Biozol | polyclonal | 0.625 μg/mL |
| Fixable Viability Dye  eFluor 780 | 13 | eBioscience | ---- | 1:1000 |
| Experiment 2: FACS analysis of B cells, monocytes, γδ T cells (TCR1), αβ T cells (TCR2 + TCR3), MHCI, MHCII, MHCII+ B cells and MHCII+ monocytes | | | | |
| mouse IgG1 anti-chicken Bu-1-AF647 | 22 | Biozol | AV20 | 1:500 |
| mouse IgG1 anti-chicken MHC I | 123 | southern Biotech | F21-2 | 1:200 |
| mouse IgG1 anti-chicken MHC II-AF488 | 118 | Biozol | 2G11 | 1:1000 |
| The primary antibodies utilized in both experiments were 42 (TCR1), 43 (TCR2), 44 (TCR3), and 23 (KUL01), alongside secondary antibodies 26 and 45, as well as the live-dead antibody 13. | | | | |

**Supplementary file 1f.** Details of various primers used for the qPCR study. The annealing temperature for all the used primers is 59 °C.

| **Primer** | **Sequence 5' to 3'** | **amplicon (bp)** | **Source** | **Accession number** |
| --- | --- | --- | --- | --- |
| FoxP3 FW | AGTACGCCACAACCTGAGCCT | 157 | 1 | MT133687.1 |
| FoxP3 RV | TTGGGGTCCTCTCAGCTCCGT |  |  |  |
| IL-12p35 FW | TGGCCGCTGCAAACG | 240 | 2 | NM_001398447.1 |
| IL-12p35 RV | CCAGCTCTGCCTTGTAGGTT |  | 3 |  |
| IL-17A FW | TTTCTGCACATGGGAAGGTG | 144 | 1 | AJ493595 |
| IL-17A RV | CCTGGTTCATGTTGCTGATGC |  |  |  |
| IL-2 FW | GAACCTCAAGAGTCTTACGGGTCTA | 111 | 4 | NM_204153.2 |
| IL-2 RV | ACAAAGTTGGTCAGTTCATGGAGA |  |  |  |
| IL-22 FW | TGTTGTTGCTGTTTCCCTCTTC | 143 | 1 | NM_001199614.1 |
| IL-22 RV | GCCAAGGTGTAGGTGCGATTCC |  |  |  |
| IL-4 FW | GTGCCCACGCTGTGCTTAC | 82 | 1 | AJ621249. |
| IL-4 RV | AGGAAACCTCTCCCTGGATGTC |  |  |  |
| IL-5 FW | GGAACGGCACTGTTGAAAAATAA | 111 | 1 | NM_001007084.2 |
| IL-5 RV | TTCTCCCTCTCCTGTCAGTTGTG |  |  |  |
| IL-6 FW | GCTTCGACGAGGAGAAATGC | 139 | 1 | NM_204628 |
| IL-6 RV | GCCAGGTGCTTTGTGCTGTA |  |  |  |
| TGF-β FW | CGGCCGACGATGAGTGGCTC | 120 | 1 | M31160.1 |
| TGF-β RV | CGGGGCCCATCTCACAGGGA |  |  |  |
| IL-10 FW | CGGGAGCTGAGGGTGAAGT | 88 | 5 | AJ621254.1 |
| IL-10 RV | GTTCAGAGCTGAGCAGTTGGATGT |  |  |  |
| IL-1β FW | GTGAGGCTCAACATTGCGCTGTA | 214 | 1 | NM_204524.2 |
| IL-1β RV | TGTCCAGGCGGTAGAAGATGAAG |  |  |  |
| Viperin FW | CAGTGGTGCCGAGATTATGC | 105 | 6 | EU427332.1 |
| Viperin RV | CACAGGATTGAGTGCCTTGA |  |  |  |
| ISG12 FW | TCCTCAGCCATGAATCCGAACA | 114 | 6 | BN000222.1 |
| ISG12 RV | GGCAGCCGTGAAGCCCAT |  |  |  |
| Mx FW | GCTCCTTCAGGAACTTCCGCTT | 115 | 1 | NM_204609.2 |
| Mx RV | TTCCCAGAGTTCCGGTCTCCAA |  |  |  |
| IFN-λ FW | CTTTGGAGTTGAAGGCAGTGTGG | 195 | 7 | XM_040703743.1 |
| IFN-λ RV | TCTGGGTTGTGGGGTTTGTGAG |  |  |  |
| STAT1 FW | TTGTAACTTCGCTATTGGTATTCC | 106 | 8 | NM_001012914 |
| STAT1 RV | TTCCGTGATGTGTCTTCCTTC |  |  |  |
| TLR3 FW | TCAGTACATTTGTAACACCCCGCC | 256 | 9 | NM_001011691 |
| TLR3 RV | GGCGTCATAATCAAACACTCC |  |  |  |
| MDA5 FW | CTCTGCGAGAAACCCAACAT | 329 | 10 | GU570144 |
| MDA5 RV | GCCCTCTGCTTCATCTTCAC |  |  |  |
| MyD88 FW | ATCCCTCATTTCTGGCATCTT | 89 | 11 | AJ851640.1 |
| MyD88 RV | CCTTCCTTATAGTTCTGGCTTCT |  |  |  |
| IFITM5 FW | TGCTTCACCAGCTAGGACTCTGC | 140 | 12 | XM_421662.4 |
| IFITM5 RV | TGGCTTTTGCTCTGTCACCACTTTG |  |  |  |
| IL8 FW | TTGGAAGCCACTTCAGTCAGAC | 120 | 13 | NM_205498 |
| IL8 RV | GGAGCAGGAGGAATTACCAGTT |  |  |  |
| IRF7 FW | CAGGAAGGATGTCACCAGCA | 117 | 14 | NM_205372.1 |
| IRF7 RV | GCGCAGCGGAAGTTGGTCTT |  |  |  |
| NF-kB1 FW- | GGACGGCGAAAGGACTCT | 208 | 15 | NM_205134.2 |
| NF-kB1 RV- | CCATTGCAAACATTTGGGGAT |  |  |  |
| SOCS3 FW | GCACCAAGAACCTGCGCATC | 103 | This study | NM_204600.2 |
| SOCS3 RV | AGCTTCAGCACGCAGTCGAA |  |  |  |
| USP18 FW | GAGCACCTGGCCTGTCAGAT | 127 | This study | XM_040658339.2 |
| USP18 RV | AGCACTGCAGGCACTTCTCC |  |  |  |
| SHP2 FW | TTGCAACTCAAGCAGCCCCT | 119 | This study | NM_204968 |
| SHP2 RV | TCCCAGAAGCCCTGCTTGAC |  |  |  |
| SOCS1 FW | AGGGTTTTGCAGCCCTCGTT | 92 | This study | *NM_001137648.1* |
| SOCS1 RV | GGCTCCCGTCCGTGCTAATT |  |  |  |
| r18S FW | CATGTCTAAGTACACACGGGCGGTA | 136 | 16 | NC_052547. 1 |
| r18S RV | GGCGCTGCTGGCATGTATTA |  |  |  |

**References for Supplementary file 1f:**

1. von Heyl, T. *et al.* Loss of αβ but not γδ T cells in chickens causes a severe phenotype. *European Journal of Immunology* **53**, 2350503 (2023).

2. Balu, S., Rothwell, L. & Kaiser, P. Production and characterisation of monoclonal antibodies specific for chicken interleukin-12. *Veterinary immunology and immunopathology* **140**, 140-146 (2011).

3. Abdul-Careem, M. *et al.* Marek's Disease Virus–Induced Transient Paralysis Is Associated with Cytokine Gene Expression in the Nervous System. *Viral immunology* **19**, 167-176 (2006).

4. Xu, F., Liu, S. & Li, S. Effects of selenium and cadmium on changes in the gene expression of immune cytokines in chicken splenic lymphocytes. *Biological Trace Element Research* **165**, 214-221 (2015).

5. He, S. *et al.* High-frequency and activation of CD4+ CD25+ T cells maintain persistent immunotolerance induced by congenital ALV-J infection. *Veterinary Research* **52**, 1-15 (2021).

6. Wang, S. *et al.* Dynamic changes in the expression of interferon-stimulated genes in joints of SPF chickens infected with avian reovirus. *Frontiers in Veterinary Science* **8**, 618124 (2021).

7. Yu, Y. *et al.* Effects of infectious bursal disease virus infection on interferon and antiviral gene expression in layer chicken bursa. *Microbial pathogenesis* **144**, 104182 (2020).

8. Truong, A.D., Hong, Y., Hoang, C.T., Lee, J. & Hong, Y.H. Chicken IL-26 regulates immune responses through the JAK/STAT and NF-κB signaling pathways. *Developmental & Comparative Immunology* **73**, 10-20 (2017).

9. Villanueva, A., Kulkarni, R. & Sharif, S. Synthetic double-stranded RNA oligonucleotides are immunostimulatory for chicken spleen cells. *Developmental & Comparative Immunology* **35**, 28-34 (2011).

10. Lee, S.B. *et al.* Targeted knockout of MDA5 and TLR3 in the DF-1 chicken fibroblast cell line impairs innate immune response against RNA ligands. *Frontiers in Immunology* **11**, 678 (2020).

11. Wang, Y. *et al.* Chicken interferon regulatory factor 7 (IRF7) can control ALV-J virus infection by triggering type I interferon production through affecting genes related with innate immune signaling pathway. *Developmental & Comparative Immunology* **119**, 104026 (2021).

12. Giotis, E. *et al.* Constitutively elevated levels of SOCS1 suppress innate responses in DF-1 immortalised chicken fibroblast cells. *Scientific Reports* **7**, 17485 (2017).

13. Li, Y., Handberg, K., Juul-Madsen, H.R., Zhang, M. & Jørgensen, P.H. Transcriptional profiles of chicken embryo cell cultures following infection with infectious bursal disease virus. *Archives of virology* **152**, 463-478 (2007).

14. Wu, W.-J. *et al.* Effects of Reticuloendotheliosis virus on TLR-3/IFN-Β pathway in specific pathogen-free chickens. *Research in Veterinary Science* **156**, 36-44 (2023).

15. Kuehu, D.L. *et al.* Effects of Heat-Induced Oxidative Stress and Astaxanthin on the NF-kB, NFE2L2 and PPARα Transcription Factors and Cytoprotective Capacity in the Thymus of Broilers. *Current Issues in Molecular Biology* **46**, 9215-9233 (2024).

16. Laparidou, M., Schlickenrieder, A., Thoma, T., Lengyel, K. & Schusser, B. Blocking of the CXCR4-CXCL12 interaction inhibits the migration of chicken B cells into the bursa of Fabricius. *Frontiers in immunology* **10**, 3057 (2020).
