# Supplementary material for: Deciphering interferon functions in avian influenza using receptor knockout models in the natural host
Source: eLife. 2026 Jun 26;14:RP107855. doi: 10.7554/eLife.107855 (PMC13309126; doi:10.7554/eLife.107855)
Supplement: MDAR checklist [file elife-107855-mdarchecklist1.docx]

**Materials Design Analysis Reporting (MDAR)**

**Checklist for Authors**

The [MDAR framework](https://osf.io/xfpn4/) establishes a minimum set of requirements in transparent reporting mainly applicable to studies in the life sciences.

*eLife* asks authors to **provide detailed information within their article** to facilitate the interpretation and replication of their work. Authors can also upload supporting materials to comply with relevant reporting guidelines for health-related research (see [EQUATOR Network](http://www.equator-network.org/%20)), life science research (see the [BioSharing Information Resource](http://biosharing.org/)), or animal research (see the [ARRIVE Guidelines](http://www.plosbiology.org/article/info:doi/10.1371/journal.pbio.1000412) and the [STRANGE Framework](https://doi.org/10.1038/d41586-020-01751-5); for details, see *eLife*’s [Journal Policies](https://reviewer.elifesciences.org/author-guide/journal-policies)). Where applicable, authors should refer to any relevant reporting standards materials in this form.

For all that apply, please note **where in the article** the information is provided. Please note that we also collect information about data availability and ethics in the submission form.

**Materials:**

| **Newly created materials** | **Indicate where provided: section/figure legend** | **N/A** |
| --- | --- | --- |
| The manuscript includes a dedicated "materials availability statement" providing transparent disclosure about availability of newly created materials including details on how materials can be accessed and describing any restrictions on access. | Materials and Methods: Generation of IFNAR1 and IFNLR1 KO primordial germ cells; Generation of IFNAR1−/− and IFNLR1−/− chickens; Animals; Data/Materials availability statement. Supplementary File 1a. Newly generated IFNAR1−/− and IFNLR1−/− chicken lines/PGC clones are described; availability should be stated in the manuscript subject to institutional and animal-use restrictions. |  |
|  |  |  |
| **Antibodies** | **Indicate where provided: section/figure legend** | **N/A** |
| For commercial reagents, provide supplier name, catalogue number and [RRID](https://scicrunch.org/resources), if available. | Materials and Methods: Flow cytometry; Western blot and RT-PCR. Supplementary File 1e lists antibody names, suppliers, clones/internal codes, and concentrations used for FACS. Western blot antibodies are described in the Methods. |  |
|  |  |  |
| **DNA and RNA sequences** | **Indicate where provided: section/figure legend** | **N/A** |
| Short novel DNA or RNA including primers, probes: Sequences should be included or deposited in a public repository. | Supplementary File 1a,b,c,f. sgRNA, ssODN, primers, probes, amplicon sizes, accession numbers, and primer sources are provided. |  |
|  |  |  |
| **Cell materials** | **Indicate where provided: section/figure legend** | **N/A** |
| Cell lines: Provide species information, strain. Provide accession number in repository OR supplier name, catalog number, clone number, OR RRID. | Materials and Methods: Experimental design; Western blot and RT-PCR. Cell lines/cell materials include MDCK cells, CEC-32 #511 reporter cells expressing firefly luciferase under the chicken Mx promoter, and chicken embryonic fibroblast cultures. Culture/selection conditions are described where used. |  |
| Primary cultures: Provide species, strain, sex of origin, genetic modification status. | Materials and Methods: Western blot and RT-PCR. Primary chicken embryonic fibroblasts were isolated from ED10 embryos of defined genotypes (WT, IFNAR1+/−, IFNAR1−/−); species, developmental stage, and genetic modification status are provided. |  |
|  |  |  |
| **Experimental animals** | **Indicate where provided: section/figure legend** | **N/A** |
| Laboratory animals or Model organisms: Provide species, strain, sex, age, genetic modification status. Provide accession number in repository OR supplier name, catalog number, clone number, OR RRID. | Materials and Methods: Animals; Experimental design; figure legends. Species/strain/source: SPF Lohman selected leghorn chickens from ValoBioMedia, housed at TUM Animal Research Center. Sex/age/genotype are provided for ED10/ED11/ED18 embryos, 1-month-old chicks, 5-week-old immunized chicks, and 27-week-old hens; genotypes include WT, IFNAR1−/−, IFNLR1−/− and heterozygous controls where relevant. |  |
| Animal observed in or captured from the field: Provide species, sex, and age where possible. | Not applicable; no field-captured animals were used. | X |
|  |  |  |
| **Plants and microbes** | **Indicate where provided: section/figure legend** | **N/A** |
| Plants: provide species and strain, ecotype and cultivar where relevant, unique accession number if available, and source (including location for collected wild specimens). | Not applicable; no plants were used. | X |
| Microbes: provide species and strain, unique accession number if available, and source. | Materials and Methods: Experimental design; Supplementary File 1d. Virus strains and titers are listed, including WSN/33 (H1N1), A/Chicken/Belgium/460/2019 (H3N1), A/chicken/Saudi Arabia/CP7/1998 (H9N2), and IBV Beaudette strain. The source of the H3N1 isolate is stated in the in vivo experimental design section. |  |
|  |  |  |
| **Human research participants** | **Indicate where provided: section/figure legend) or state if these demographics were not collected** | **N/A** |
| If collected and within the bounds of privacy constraints report on age, sex, gender and ethnicity for all study participants. | Not applicable; no human participants were included. | X |

**Design:**

| **Study protocol** | **Indicate where provided: section/figure legend** | **N/A** |
| --- | --- | --- |
| If the study protocol has been pre-registered, provide DOI. For clinical trials, provide the trial registration number OR cite DOI. | Not applicable; the study was not a clinical trial and no preregistered study protocol/DOI applies. | X |
|  |  |  |
| **Laboratory protocol** | **Indicate where provided: section/figure legend** | **N/A** |
| Provide DOI OR other citation details if detailed step-by-step protocols are available. | Materials and Methods cite previously established protocols for PGC isolation/culture, germline transmission, CEF culture, viral propagation/titration, microbiome processing, ELISA, TCR sequencing, qPCR and flow cytometry. Full citations are provided in the References. |  |
|  |  |  |
| **Experimental study design (statistics details) *** | | |
| **For in vivo studies: State whether and how the following have been done** | **Indicate where provided: section/figure legend. If it could have been done, but was not, write “not done”** | **N/A** |
| Sample size determination | Materials and Methods: Experimental design; figure legends. Sample sizes were determined based on standard practice in the field and prior experience. Exact n values are provided in the Methods and figure legends where applicable. |  |
| Randomisation | Materials and Methods: Experimental design. Animals were grouped by genotype; individuals within each genotype were randomly selected for relevant experiments. In vivo infected animals were selected randomly within genotype. For in ovo experiments, embryos were genotyped after sample collection and then assigned to WT/homozygous groups. |  |
| Blinding | Materials and Methods: Experimental design. The in ovo viral challenge was performed blinded, with investigators unaware of genotype during infection and sample processing. Blinding was not reported for all other analyses. |  |
| Inclusion/exclusion criteria | Materials and Methods: Experimental design; TCR repertoire analysis; RNA isolation/cDNA synthesis/qRT-PCR; Figure 8-figure supplement 1 legend. Inclusion/exclusion was based on genotype, humane endpoints/euthanasia timing, RNA quality (RIN ≥ 8 for TCR), and stated sampling windows; only WT and homozygous embryos were selected for specified in ovo analyses. |  |
|  |  |  |
| **Sample definition and in-laboratory replication** | **Indicate where provided: section/figure legend** | **N/A** |
| State number of times the experiment was replicated in the laboratory. | Materials and Methods: Experimental design; figure legends. The manuscript reports the number of animals/embryos/cells per group where relevant. Experiments included independent biological replicates (animals, embryos, tissues/cells) and some technical replicates for assays. |  |
| Define whether data describe technical or biological replicates. | Materials and Methods: qRT-PCR states each gene was analyzed in triplicate. Biological replicates are animals/embryos/tissues/cell cultures as described in the experimental design and figure legends; technical replicates include qPCR triplicates and assay replicates where applicable. |  |
|  |  |  |
| **Ethics** | **Indicate where provided: section/submission form** | **N/A** |
| Studies involving human participants: State details of authority granting ethics approval (IRB or equivalent committee(s), provide reference number for approval. | Not applicable; no human participants were included. | X |
| Studies involving experimental animals: State details of authority granting ethics approval (IRB or equivalent committee(s), provide reference number for approval. | Materials and Methods: Animals. Animal experiments were approved by the government of Upper Bavaria under licenses ROB-55.2-2532.Vet_02-20-13 and ROB-55.2-2532.Vet_02-23-100. |  |
| Studies involving specimen and field samples: State if relevant permits obtained, provide details of authority approving study; if none were required, explain why. | Not applicable; no field specimens requiring permits were used. | X |
|  |  |  |
| **Dual Use Research of Concern (DURC)** | **Indicate where provided: section/submission form** | **N/A** |
| If study is subject to dual use research of concern regulations, state the authority granting approval and reference number for the regulatory approval. | Not applicable; the study is not described as subject to DURC regulations. Low pathogenic avian influenza strains/laboratory virus strains were used as described in Supplementary File 1d. | X |

**Analysis:**

| **Attrition** | **Indicate where provided: section/figure legend** | **N/A** |
| --- | --- | --- |
| Describe whether exclusion criteria were pre-established. Report if sample or data points were omitted from analysis. If yes, report if this was due to attrition or intentional exclusion and provide justification. | Materials and Methods: Experimental design; TCR repertoire analysis; RNA isolation/cDNA synthesis/qRT-PCR; Figure 8-figure supplement 1 legend. Exclusions/selection were based on pre-specified genotype assignment, sampling time, humane endpoints, and RNA quality criteria. Relevant n values are reported in the figure legends. |  |
|  |  |  |
| **Statistics** | **Indicate where provided: section/figure legend** | **N/A** |
| Describe statistical tests used and justify choice of tests. | Materials and Methods: Statistical analysis; figure legends. Statistical tests included one-way or two-way ANOVA with Tukey post hoc tests, Wilcoxon rank-sum pairwise tests, PERMANOVA, and negative binomial generalized linear modeling with Holm-adjusted post-hoc comparisons for TCR repertoire analyses. Significance threshold P ≤ 0.05. |  |
|  |  |  |
| **Data availability** | **Indicate where provided: section/submission form** | **N/A** |
| For newly created and reused datasets, the manuscript includes a data availability statement that provides details for access (or notes restrictions on access). | Data availability section/submission form. All data supporting the findings are stated to be included in the article, figure source data files, and supplementary files. |  |
| When newly created datasets are publicly available, provide accession number in repository OR DOI and licensing details where available. | Data availability section/submission form. Microbiome sequencing data: NCBI BioProject PRJNA1472969. TCR repertoire sequencing data: NCBI BioProject PRJNA1473170. Figure source data and gel/blot source data are provided with the submission. |  |
| If reused data is publicly available provide accession number in repository OR DOI, OR URL, OR citation. | Materials and Methods/References. Reused public resources/software/databases are cited or linked, including IMNGS2, RHEA, FastQC, MultiQC, MiXCR, R/tidyverse, MASS, and emmeans. |  |
|  |  |  |
| **Code availability** | **Indicate where provided: section/figure legend** | **N/A** |
| For any computer code/software/mathematical algorithms essential for replicating the main findings of the study, whether newly generated or re-used, the manuscript includes a data availability statement that provides details for access or notes restrictions. | Materials and Methods: Microbiome analysis; TCR repertoire analysis; Statistical analysis. No newly generated custom software/code is described as essential for replicating the main findings; analyses used established software and R packages with versions/citations where provided. |  |
| Where newly generated code is publicly available, provide accession number in repository, OR DOI OR URL and licensing details where available. State any restrictions on code availability or accessibility. | Not applicable; no newly generated custom code repository is reported. | X |
| If reused code is publicly available provide accession number in repository OR DOI OR URL, OR citation. | Materials and Methods and References provide URLs/citations/versions for reused software and packages, including FastQC v0.12.1, MultiQC v1.23, MiXCR v4.7.0, R v4.4.1/tidyverse v2.0.0, MASS v7.3-61 and emmeans v1.10.3. |  |

**Reporting:**

The MDAR framework recommends adoption of discipline-specific guidelines, established and endorsed through community initiatives.

| **Adherence to community standards** | **Indicate where provided: section/figure legend** | **N/A** |
| --- | --- | --- |
| State if relevant guidelines (e.g., ICMJE, MIBBI, ARRIVE, STRANGE) have been followed, and whether a checklist (e.g., CONSORT, PRISMA, ARRIVE) is provided with the manuscript. | MDAR checklist provided with the manuscript. Animal work is reported with species/strain/source, sex/age/genotype, ethics approval, sample sizes, randomization/blinding where applicable, statistical methods, and data availability details in the Materials and Methods, figure legends, Supplementary File 1, and submission form. ARRIVE-relevant reporting elements are included where applicable. |  |

* We provide the following guidance regarding transparent reporting and statistics; we also refer authors to [Ten common statistical mistakes to watch out for when writing or reviewing a manuscript](https://doi.org/10.7554/eLife.48175).

**Sample-size estimation**

- You should state whether an appropriate sample size was computed when the study was being designed
- You should state the statistical method of sample size computation and any required assumptions
- If no explicit power analysis was used, you should describe how you decided what sample (replicate) size (number) to use

**Replicates**

- You should report how often each experiment was performed
- You should include a definition of biological versus technical replication
- The data obtained should be provided and sufficient information should be provided to indicate the number of independent biological and/or technical replicates
- If you encountered any outliers, you should describe how these were handled
- Criteria for exclusion/inclusion of data should be clearly stated
- High-throughput sequence data should be uploaded before submission, with a private link for reviewers provided (these are available from both GEO and ArrayExpress)

**Statistical reporting**

- Statistical analysis methods should be described and justified
- Raw data should be presented in figures whenever informative to do so (typically when N per group is less than 10)
- For each experiment, you should identify the statistical tests used, exact values of N, definitions of center, methods of multiple test correction, and dispersion and precision measures (e.g., mean, median, SD, SEM, confidence intervals; and, for the major substantive results, a measure of effect size (e.g., Pearson's r, Cohen's d)
- Report exact p-values wherever possible alongside the summary statistics and 95% confidence intervals. These should be reported for all key questions and not only when the p-value is less than 0.05.

**Group allocation**

- Indicate how samples were allocated into experimental groups (in the case of clinical studies, please specify allocation to treatment method); if randomization was used, please also state if restricted randomization was applied
- Indicate if masking was used during group allocation, data collection and/or data analysis
