# Supplementary material for: Deciphering interferon functions in avian influenza using receptor knockout models in the natural host
Source: eLife. 2026 Jun 26;14:RP107855. doi: 10.7554/eLife.107855 (PMC13309126; doi:10.7554/eLife.107855)
Supplement: Peer review history

# Editor report: eLife Assessment

**DOI:** 10.7554/eLife.107855.3.sa0

**Authors:** John W Schoggins

**Affiliations:** The University of Texas Southwestern Medical Center United States

**Keywords:** Compelling, Solid, Important

This study reports on the development and characterization of chickens with genetic deficiencies in type I or type III interferon receptors, which is an **important** contribution to the field of avian immunology. The data reflecting the development of the new interferon-receptor-deficient chickens is **compelling**. The initial characterization of IFN biology and infection responses in these knockout chickens provides a **solid** foundation for future studies on the distinct contributions of type I and type III interferon signaling to antiviral responses.

# Referee report: Reviewer #2 (Public review)

**DOI:** 10.7554/eLife.107855.3.sa1

Summary:

This is a laudable effort to help dissect the contributions of type I and type III IFNs to the antiviral response in chicken and therefore represents an important piece of work, not least in the light of birds being a key carrier and worldwide distributor of influenza virus. The first part of the study characterises the generation of IFNAR and IFNLR KO chicken strains and describes basic differences. Four different viruses are then tested in chicken embryos, while the subsequent analysis of the antiviral response in vivo is performed with one influenza H3N1 strain.

Strengths:

Having these two KO chicken strains as a tool is a great achievement. The initial analysis is solid. Clear effect of IFNAR deficiency in in vivo infection, less so for IFNLR deficiency.

Weaknesses:

(1) The antibody induction by KLH immunisation: We still don't know whether or not this vaccination induces IFN responses in wt mice, so it is still not possible to judge whether the effects observed are due to steady-state differences or to differential effects of IFN induced during the vaccination phase. Pre-immune results are now shown and are indeed zero. As suggested, the whole figure 4 is now condensed into one or two panels by proper calculation of Ab titers - would these titres be significantly different? This as all of the other in vivo experiments have not been repeated if I understand the methods section correctly. I understand that there are three R restrictions that are tighter in some countries, and I accept that with the numbers used here, some statistical significance is reached, but this is for instance not the case for survival.

(2) The basic conundrum here and in later figures is now addressed by the authors in the discussion: Situations where IFN type 1 and 3 signalling deficiency each have an independent effect (i.e. fig.4d) suggest that they act by separate, unrelated mechanisms. However, all the literature about these IFN families suggest that they show almost identical signalling and gene induction downstream of their respective receptors. How can the same signalling, clearly active here downstream of the receptors for IFN type 1 or type 3, be non-redundant, i.e. why does the unaffected IFN family not stand in? The mouse studies, which showed a rather subtle phenotype when only one of the two IFN systems was missing, but a massive reduction in virus control in double KO mice, are discussed, but a clear-cut explanation for the differences has not been reached. Reasons could be a direct effect of IFNab on B cells and an indirect effect of IFNL through non-B cells, timing issues, and many other scenarios can be envisaged. The authors do not address this question experimentally, which limits the depth of analysis, they have however now included a discussion of this dilemma.

(3) In the one in vivo experiment performed with chickens, only one virus tested, more influenza strains should be included as well as non-influenza viruses. I appreciate that this is logistically difficult.

(4) The basic conundrum of point 2 applies equally to Fig. 6a, both KOs have a phenotype. Again, in 6d, both IFNs appear to be separately required for Mx induction. An explanation has been attempted, but more experiments, for instance looking at different time points to understand if we are dealing simply with different kinetics of the response, have not been attempted, despite the fact that such experiments are likely not covered by strict three R rules.

(5) The in vivo infection is the most interesting experiment, and the key outcome here is that IFN type 1 is crucial for anti-H3N1 protection in chickens, while type 3 is less impactful. However, this experiment suffers from the different time points when chickens were culled, so many parameters are impossible to compare (e.g. weight loss, histopathology). Some explanation is given as to the comparisons chosen here, but a more thorough analysis at several time points would have strengthened this study.

Comments on revised version:

In the rebuttal, the authors have gone to some length to add to the discussion of the experiments, and some aspects are better explained now than before. Many of these explanations remain speculative however, so the study remains inconclusive in several aspects. As no new data was added, my overall judgement of this study remains unchanged.

# Author comment: Author response

**DOI:** 10.7554/eLife.107855.3.sa2

**Authors:** Mohanned Naif Alhussien, Hanna Kaisa Vikkula, Romina Klinger, Christian Zenner, Simon P Frueh, Rashi Negi, Theresa von Heyl, Sabrina Schleibinger, Milena Brunner, Tom VL Berghof, Leora Avolio, Arne Reich, Benjamin Schade, Bassel Aboukhadra, Silke Rautenschlein, Rudolf Preisinger, Hicham Sid, Benjamin Schusser

**Affiliations:** Technical University of Munich Freising Germany; Technical University of Munich Freising Germany; Technical University of Munich Freising Germany; Technical University of Munich Freising Germany; Ludwig-Maximilians-Universität München Muenchen Germany; Technical University of Munich Freising Germany; Technical University of Munich Freising Germany; Technical University of Munich Freising Germany; Technical University of Munich Freising Germany; Technical University of Munich Freising Germany; Technical University of Munich Freising Germany; Technical University of Munich Freising Germany; Bavarian Animal Health Service Poing Germany; University of Veterinary Medicine Hannover, Foundation Hannover Germany; University of Veterinary Medicine Hannover, Foundation Hannover Germany; EW GROUP GmbH Visbek Germany; Technical University of Munich Freising Germany; Technical University of Munich Freising Germany

**Public Reviews:**

**Reviewer #1 (Public review):**

Summary:

This manuscript presents an extensive body of work and an outstanding contribution to our understanding of the IFN type I and III system in chickens. The research started with the innovative approach of generating KO chickens that lack the receptor for IFNα/β (IFNAR1) or IFN-λ (IFNLR1). The successful deletion and functional loss of these receptors was clearly and comprehensively demonstrated in comparison to the WT. Moreover, the homozygous KO lines (IFNAR1-/- or IFNLR1-/-) were found to have similar body weights, and normal egg production and fertility compared to their WT counterparts. These lines are a major contribution to the toolbox for the study of avian/chicken immunology.

The significance of this contribution is further demonstrated by the use of these lines by the authors to gain insight into the roles of IFN type I and IFN-type III in chickens, by conducting in ovo and in vivo studies examining basic aspects of immune system development and function, as well as the responses to viral challenges conducted in ovo and in vivo.

Based on solid, state-of the-art methods and convincing evidence from studies comparing various immune system related functions in the IFNAR1-/- or IFNLR1-/- lines to the WT, revealed that the deletion of IFNAR1 and/or IFNLR1 resulted in:

(1) impaired IFN signaling and induction of anti-viral state;

(2) modulation of immune cell profiles in the peripheral blood circulation and spleen;

(3) modulation of the cecum microbiome;

(4) reduced concentrations of IgM and IgY in the blood plasma before and following immunization with model antigen KLH, whereby also line differences in the time-course of the antibody production were observed;

(5) decrease in MHCII+ macrophages and B cells in the spleen of IFNAR1 KO chickens, although the MHCII-expression per cell was not affected in this line; and

(6) reduction in the response of αβ1 TCR+ T cells of IFNAR1 KO chickens as suggested by clonal repertoire analyses.

These studies were then followed by examination of the role of type I and type III IFN in virus infection, using different avian influenza A virus strains as well as an avian gamma corona virus (IBV) in in ovo challenge experiments. These studies revealed: viral titers that reflect virus-species and strain-specific IFN responses; no differences in the secretion of IFN-α/β in both KO compared to the WT lines; a predominant role of type I IFN in inducing the interferon-stimulated gene (ISG) Mx; and that an excessive and unbalanced type I IFN response can harm host fitness (survival rate, length of survival) and contribute to immunopathology.

Based on guidance from the in ovo studies, comprehensive in vivo studies were conducted on host-pathogen interactions in hens from the three lines (WT, IFNAR1 KO, or IFNLR1 KO). These studies revealed the early appearance of symptoms and poor survival of hens from the IFNR1 KO line challenged with H3N1 avian influenza A virus; efficient H#N1 virus replication in IFNAR1 KO hens, increased plasma concentrations of IFNα/β and mRNA expression of IFN-λ in spleens of the IFNAR1 KO hens; a pro-inflammatory role of IFN-λ in the oviduct of hens infected with H3N1 virus; increased proinflammatory cytokine expression in spleens of IFNAR1 KO hens, and Impairment of negative feedback mechanisms regulating IFN-α/β secretion in IFNAR1-KO hens and a significant decrease in this group's antiviral state; additionally it was demonstrated that IFN-α/β can compensate IFN-λ to induce an adequate antiviral state in the spleen during H3N1 infection, but IFN-λ cannot compensate for IFN-α/β signaling in the spleen.

Strengths:

(1) Both the methods and results from the comprehensive, well-designed, and well-executed experiments are considered excellent. The results are well and correctly described in the result narrative and well presented in both the manuscript and supplement Tables and Figures. Excellent discussion/interpretation of results.

(2) The successful generation of the type I and type III IFN KO lines offers unprecedented insight and opens multiple new venues for exploring the IFN system in chickens. The new knowledge reported here is direct evidence of the high impact of this model system on effectively addressing a critical knowledge gap in avian immunology.

(3) The thoughtful selection of highly relevant viruses to poultry and human health for the in ovo and in vivo challenge studies to examine and assess host-pathogen interactions in the IFNR KO and WT lines.

(4) Making use of the unique opportunities in the chicken model to examine and evaluate the host's IFN system responses to various viral challenges in ovo, before conducting challenge studies in hens.

(5) The new knowledge gained from the IFNAR1 and IFNLR1 KO lines will find much-needed application in developing more effective strategies to prevent health challenges like avian influenza and its devastating effects on poultry, humans, and other mammals.

(6) The excellent cooperation and contributions of the co-authors and institutions.

Weaknesses:

No weaknesses were identified by this reviewer.

We thank Reviewer #1 for the very positive and thoughtful evaluation of our manuscript. We appreciate the recognition of the effort involved in generating and characterizing the IFNAR1^-/-^ and IFNLR1^-/-^ chicken lines and for highlighting their significance as valuable tools for advancing avian immunology.

We are grateful for the reviewer’s clear summary of our findings and for acknowledging the quality of the experimental design, data presentation, and interpretation. The encouraging feedback affirms the broader impact of our study and its contribution to understanding type I and type III interferon biology and antiviral defense mechanisms in chickens.

We have carefully considered all reviewer comments and revised the manuscript accordingly to further clarify methodological details and improve the presentation of our results.

**Reviewer #1 (Recommendations for the authors):**

Minor suggestions/corrections:

(1) Line 192, 193, 196 - the superscript "+" sign appears to be underlined.

We corrected the formatting of all superscript "+" symbols (L 192-196).

(2) L195: ...in the spleen "of both IIFNR KO lines" (or some clarification of what you are comparing).

The sentence was revised to read “in the spleen of both IFNR knockout lines” for clarity (L 195).

(3) L198: replace "highlighting" with "and".

“Highlighting” was replaced with “and” as suggested (L 198).

(4) L231 and 235: change "monocytes" to "macrophages" as this description appears to refer to spleen cells. Also, make this change in Figure 3b and in the Figure 3 caption (e.g. monocytes/macrophages).

“Monocytes” was replaced with “macrophages” to accurately describe spleen cells. The same correction was made in Figure 3b and the Figure 3 caption as well as in the supplementary Figure 4 (L 229-234).

(5) L257: indicate this significant difference in Figure 5b.

The significant difference has now been clearly indicated in Figure 5b.

(6) L420, 421: change "monocytes" to "macrophages" as this discussion appears to refer to the spleen.

“Monocytes” was replaced with “macrophages” to reflect the correct cell type discussed in the spleen context (L 226-227).

(7) L564-565: has the anti-human MX antibody been shown to cross-react with chicken Mx?

We thank the reviewer for this valuable comment. Yes, the cross-reactivity of the anti-human MxA monoclonal antibody (clone M143, mouse IgGκ; Merck, Germany) with chicken Mx protein has been previously demonstrated. This antibody has been used successfully to detect chicken Mx in several published studies, including Schusser et al., Journal of Virology (2011). Accordingly, supporting references have been added to the revised manuscript (L584-586).

(8) L608: how were PBMC and splenocytes (mononuclear spleen cells?) isolated -Line 647 on page 14 mentions their isolation using Histopaque-1077 density gradient centrifugation

We thank the reviewer for this helpful comment. A detailed description of the isolation procedure for PBMCs and mononuclear spleen cells has now been added to the Materials and Methods section under the new subsection titled “Isolation of peripheral blood and splenic mononuclear cells” In this section, we specify that both PBMCs and splenic mononuclear cells were isolated using Histopaque-1077 density gradient centrifugation as described on page (14), lines (668-676)

**Reviewer #2 (Public review):**

Summary:

This study attempts to dissect the contributions of type I and type III IFNs to the antiviral response in chickens. The first part of the study characterises the generation of IFNAR and IFNLR KO chicken strains and describes basic differences. Four different viruses are then tested in chicken embryos, while the subsequent analysis of the antiviral response in vivo is performed with one influenza H3N1 strain.

Strengths:

Having these two KO chicken strains as a tool is a great achievement. The initial analysis is solid. Clear effect of IFNAR deficiency in in vivo infection, less so for IFNLR deficiency.

Weaknesses:

(1) The antibody induction by KLH immunisation: No data indicated whether or not this vaccination induces IFN responses in wt mice, so the effects observed may be due to steady-state differences or to differential effects of IFN induced during the vaccination phase. No pre-immune results are shown. The differences are relatively small and often found at only one plasma dilution - the whole of Figure 4 could be condensed into one or two panels by proper calculation of Ab titers - would these titres be significantly different? This, as all of the other in vivo experiments, has not been repeated, if I understand the methods section correctly.

We thank the reviewer for the valuable comments and helpful suggestions.

Regarding interferon induction by KLH immunisation, we agree that KLH is not known to strongly induce type I or type III interferon responses. Importantly, the goal of this experiment was not to quantify IFN induction per se, but to assess how the absence of IFN receptors affects adaptive antibody responses under standard immunisation conditions. KLH is a highly immunogenic, copper‑containing extracellular oxygen‑carrier protein derived from the marine gastropod *Megathura crenulata* and is widely used as a T cell–dependent model antigen to study B‑cell activation, antibody production, and class switching in vivo (Harris & Markl, Micron 1999, doi: 10.1016/s0968-4328(99)00036-0; Schusser et al., 2016, doi: 10.1002/eji.201546171). Because chickens are extremely unlikely to encounter KLH under natural conditions, KLH behaves as a neo‑antigen, and anti‑KLH antibodies can be considered to arise from de novo adaptive responses rather than pre‑existing antigen experience. Owing to its structural complexity and unusual glycosylation, KLH provides broad antigenic stimulation and engages adaptive immune mechanisms largely independently of pathogen‑specific innate pattern recognition, while still supporting robust T helper cell responses (Swaminathan et al., 2014, doi: 10.1111/bcp.12422; Geyer et al., 2004, doi: 10.1016/j.micron.2003.10.033). This makes KLH particularly suitable for dissecting intrinsic differences in adaptive immune responses between genotypes.

We have now included pre-immune plasma controls (Figure 4 c, d), demonstrating that baseline antibody levels did not differ statistically between groups and were negligible prior to immunisation.

As for the use of different plasma dilutions, this was necessary to ensure that all samples were measured within the linear detection range of our in-house ELISA. For example, after the primary immunisation, IgY concentrations were relatively low (e.g., day 5 post-immunisation), and plasma samples had to be diluted only 1:100 to detect measurable differences between groups. In contrast, after the booster immunisation, IgY concentrations increased substantially, and lower dilutions such as 1:100 led to signal saturation. Therefore, higher dilutions (up to 1:1600) were required to keep the values within the measurable range.

Following the reviewer’s recommendation, we have now unified the presentation of results by showing data at a single representative dilution for each isotype: 1:100 for IgM (Figure 4C) and 1:1600 for IgY (Figure 4D). These dilutions fall within the linear part of the standard curve to distinguish between groups. We also calculated endpoint antibody titers, which confirmed that the observed differences remain statistically significant (p < 0.05).

Regarding experimental replication, the study design already incorporated sufficient biological replication and longitudinal sampling to ensure robustness of the findings. Each experimental group consisted of ten animals, including three animals that served as negative controls. In addition, animals were sampled at multiple time points following immunisation, allowing the dynamics of the antibody response to be monitored over time. This longitudinal design provides repeated biological measurements within the same experimental cohort and allows confirmation of consistent response patterns across time points. All ELISA measurements were performed in technical triplicates. Together, the combination of adequate group size, appropriate controls, repeated sampling over time, and technical replication provides sufficient statistical power and internal validation of the observed effects. Furthermore, all animal experiments were conducted under strict approval of the Government of Upper Bavaria and in accordance with German animal welfare regulations, which limit unnecessary repetition of in vivo experiments beyond the approved experimental design.

(2) The basic conundrum here and in later figures is never addressed by the authors: Situations where IFN type 1 and 3 signalling deficiency each have an independent effect (i.e., Figure 4d) suggest that they act by separate, unrelated mechanisms. However, all the literature about these IFN families suggests that they show almost identical signalling and gene induction downstream of their respective receptors. How can the same signalling, clearly active here downstream of the receptors for IFN type 1 or type 3, be non-redundant, i.e., why does the unaffected IFN family not stand in? This is a major difference from the mouse studies, which showed a rather subtle phenotype when only one of the two IFN systems was missing, but a massive reduction in virus control in double KO mice (the correct primary paper should be quoted here, not only the review by McNab). Reasons could be a direct effect of IFNab on B cells and an indirect effect of IFNL through non-B cells, timing issues, and many other scenarios can be envisaged. The authors do not address this question, which limits the depth of analysis.

We thank the reviewer for this insightful comment. Indeed, this represents one of the most interesting and novel findings of our study. Unlike in mice, where both type I and type III interferon systems need to be disrupted to observe clear susceptibility to influenza infection, in our chicken model the loss of IFNAR1 alone was sufficient to render the animals highly susceptible. This highlights a key difference between mammalian and avian interferon biology and supports the main goal of our work, to investigate the specific biological activities of avian interferons rather than directly transferring conclusions from mammalian systems.

In relation to Figure 4d (anti-KLH IgY), we observed that both IFNAR1^-/-^ and IFNLR1^-/-^ animals reduced IgY levels compared to wild type at day 3 after the booster immunisation. However, by day 5 post-booster, IgY levels in IFNLR1^-/-^ animals had returned to wild-type levels, while IFNAR1-/- animals still showed significantly lower IgY. This indicates that type III IFN contributes to the early phase of the IgY response but that its absence can later be compensated by type I IFN signalling. In contrast, loss of type I IFN cannot be compensated by type III IFN, suggesting that type I IFN plays a more dominant or sustained role in antibody induction.

Although type I and type III IFNs share overlapping signaling pathways and induce similar sets of ISGs, their effects are not entirely redundant in chickens. A likely explanation is the difference in receptor distribution: IFNAR1 is broadly expressed across most cell types, while IFNLR1 expression is mainly confined to epithelial cells (Reuter et al. 2014, doi: 10.1128/jvi.02764-13; Santhakumar et al., 2017, doi: 10.3389/fimmu.2017.00049). This systemic versus localized receptor pattern likely determines the range of responsive cells and may account for the differential outcomes observed when either receptor is absent.

Taken together, our findings indicate that while type I and type III IFNs share overlapping signaling mechanisms, they maintain distinct biological functions in chickens, consistent with their differing receptor expression and cellular responsiveness. This contrasts with mammalian models, where redundancy between these systems is more apparent and only double knockouts show strong phenotypes especially during influenza infection (Mordstein et al., 2008, doi: 10.1371/journal.ppat.1000151; Mordstein et al., 2010, doi: 10.1128/jvi.00272-10). We have now cited this primary study instead of the McNab review and expanded the Discussion to reflect this interpretation (Page 10, Line 463-467).

(3) In the one in vivo experiment performed with chickens, only one virus was tested; more influenza strains should be included, as well as non-influenza viruses.

We thank the reviewer for this valuable suggestion. The main objective of the present study was to generate and characterize novel chicken models lacking type I and type III interferon receptors in order to investigate their physiological relevance and to obtain the first insights into their roles during viral infection with more emphasis on avian influenza. As part of this manuscript, we performed detailed in ovo experiments using both influenza and non-influenza viruses (Figure 6). These included three influenza strains: H1N1, a mammalian-adapted strain; H3N1, a low pathogenic avian strain showing features of high pathogenicity; and H9N2, a low pathogenic avian strain, as well as a non-influenza virus, the infectious bronchitis virus (IBV). The in ovo analyses revealed clear strain-dependent modulation of interferon responses, and have provided a comprehensive overview of virus-specific interferon activity in chickens. The subsequent in vivo experiment was therefore designed as a proof of concept using the most suitable viral strain to robustly challenge the immune system and to identify the distinct functions of chicken interferons.

(4) The basic conundrum of point 2 applies equally to Figure 6a; both KOs have a phenotype. Again in 6d, both IFNs appear to be separately required for Mx induction. An explanation is needed.

We thank the reviewer for raising this important point. We have revised the Discussion (page 10, lines 442-454) and provided supporting references to clarify how the composition of the chorioallantoic membrane (CAM) and virus tropism together determine the apparent requirement for type I and type III interferons. The CAM contains both epithelial and mesodermal–vascular layers, which support complementary interferon functions: type I IFN acts mainly in systemic and vascular compartments, while type III IFN provides localized protection at the epithelial surface. Consequently, viruses that replicate in both compartments (e.g., WSN33, H3N1) require both IFN pathways for maximal Mx induction (Figures 6a, 6d), whereas viruses with a predominant or prolonged epithelial phase (e.g., H9N2, IBV) at the time point analyzed are effectively controlled by type I IFN signaling alone.

These differences likely reflect virus-specific factors, including cell tropism, replication kinetics, and the spatial–temporal dynamics of receptor expression and signaling. Notably, our measurement of Mx expression at 24 hours post infection (hpi) may represent a phase when type I IFN signaling is dominant and can compensate for the absence of type III IFN. It remains possible that IFN-λ plays a more critical, non-redundant role at earlier stages post infection, when rapid antiviral protection is first required at the epithelial surface. Thus, the apparent redundancy observed at 24 hpi likely reflects temporal compensation and crosstalk between the IFN pathways rather than a lack of biological relevance for type III IFN.

(5) Line 308, where are the viral titers you refer to in the text? The statement that the results demonstrate that excessive IFNab has a negative impact is overstretched, as no IFN measurements of the infected embryos are shown here.

We thank the reviewer for this comment and would like to clarify that measurements of type I IFN (IFN-α/β) concentrations were indeed performed. The data are presented in Figure 6b and cited in the Results section (“Knockout of IFNAR1 and IFNLR1 did not affect IFN-α/β secretion in ovo”). To avoid misunderstanding, the Results section has been revised to explicitly reference the IFN-α/β measurements supporting this conclusion (line 302-309).

These data indicate that all genotypes produced comparable IFN-α/β levels upon viral infection, with the IBV infection inducing approximately tenfold higher IFN-α/β secretion than the influenza strains tested (Figure 6b). The interpretation that an excessive type I IFN response can negatively affect host fitness is based on the combination of quantified IFN-α/β data (Figure 6b) and survival probability results (Supplementary Figure 10), where embryos exhibiting the highest IFN-α/β levels (embryos of all genotypes infected with IBV and embryos infected with IFNLR1^-/-^ H9N2) showed the poorest survival despite moderate or low viral titers.

(6) The in vivo infection is the most interesting experiment, and the key outcome here is that IFN type 1 is crucial for anti-H3N1 protection in chickens, while type 3 is less impactful. However, this experiment suffers from the different time points when chickens were culled, so many parameters are impossible to compare (e.g., weight loss, histopathology, IFN measurements, and more). Many of these phenomena are highly dynamic in acute virus infections, so disparate time points do not allow a meaningful comparison between different genotypes. What are the stats in 7b? Is the median rather than the mean indicated by the line? Otherwise, the lines appear in surprising places. SD must be shown, and I find it difficult to believe that there is a significant difference in weight, for e.g., IFNAR KO, unless maybe with a paired t test. What is the statistical test?

We thank the reviewer for these thoughtful comments and agree that disease progression and sampling time can influence comparisons in acute infection studies. Hens were euthanized upon reaching predefined humane endpoint scores in full compliance with the Bavarian animal welfare regulations. Because the infection produced markedly different clinical kinetics among genotypes, all data were interpreted with reference to matched disease stages rather than absolute days post-infection.

For matched comparisons: Viral titers in the trachea and cloaca, as well as plasma IFN-α/β concentrations, were compared between day 2 in IFNAR1^-/-^ hens and day 3 in WT and IFNLR1^-/-^ hens, which represent equivalent clinical stages before the sharp viral rise seen later in WT and IFNLR1^-/-^ birds. At these comparable stages, viral titers were still low and IFN-α/β concentrations remained significantly lower in WT and IFNLR1^-/-^ than in IFNAR1^-/-^ hens (Figure 7c, d, f), indicating that uncontrolled viral replication and IFN-α/β secretion in the absence of type I signaling occur earlier and more intensely.

For Figure 7b: Because chickens reached humane endpoints at different days post infection (2 dpi for IFNAR1^-/-^ and 5–7 dpi for WT and IFNLR1^-/-^), statistical comparisons were performed within each genotype using paired t-tests and all data were shown together as mean ± SD.

We acknowledge that unequal survival times limit direct temporal comparison. However, the consistent pattern across all parameters including early severe disease, high viral load, and excessive IFN-α/β secretion in IFNAR1^-/-^ hens versus delayed onset in WT and IFNLR1^-/-^, supports the conclusion that type I IFN signaling is essential for early viral restriction and host survival, while type III IFN contributes mainly to localized inflammatory responses. The experiment cannot be repeated under the current animal welfare authorization.

(7) Figures 7e,f: these comparisons are very difficult to interpret as the virus loads at these time points already differ significantly, so any difference could be secondary to virus load differences.

We thank the reviewer for this valuable comment. We agree that viral load can influence interferon induction; however, our comparisons in Figures 7e and 7f were designed to reflect equivalent stages of disease progression rather than identical time points post-infection. For IFN-λ mRNA expression (Fig. 7e), spleens from IFNAR1^-/-^ hens were sampled on day 2 post-infection, when viral titers were maximal, and compared to WT and IFNLR1^-/-^ hens sampled on day 5 post-infection, at which point viral titers reached comparable levels. Thus, this comparison represents the phase of peak infection and systemic immune activation across all genotypes rather than an absolute temporal comparison.

Similarly, for IFN-α/β concentrations (Fig. 7f), two levels of comparison were made: between IFNAR1^-/-^ hens at day 2 post-infection (high viral titer) and WT and IFNLR1^-/-^ hens at day 3 (low viral titer), and between WT and IFNLR1^-/-^ hens at day 5 post-infection (high viral titer). In both cases, IFN-α/β levels remained disproportionately elevated in IFNAR1^-/-^ hens, indicating that the excessive type I IFN response is primarily due to the loss of receptor-mediated feedback regulation rather than viral load alone.

We have clarified this rationale in the legend of figure 7 and in the results (Line 338-345). We believe these results are valuable as they provide important insight into the temporal dynamics and regulatory interplay between type I and type III interferons during avian influenza infection.

**Reviewer #2 (Recommendations for the authors):**

Experiments need to be repeated. Comparisons in infection experiments must be done on the same day. More viruses need to be tested.

We thank the reviewer for these constructive recommendations. All infection experiments were conducted under approved animal welfare regulations, which limited the number of replicates and prevented repeating in vivo challenges beyond the authorized design, in line with the 3R principles, particularly Reduction, to avoid unnecessary animal use. To ensure comparability, samples were analyzed at matched disease stages rather than identical time points, as clarified in the revised figure legends (figure 7) and Results (Line 338-345). The study already includes multiple influenza and non-influenza viruses (H1N1, H3N1, H9N2, and IBV) tested in ovo to capture virus-specific interferon responses, while the in vivo H3N1 infection served as a proof-of-concept to dissect genotype-specific immune dynamics.
